# A Comprehensive Biophysical Model of Ion and Water Transport in Plant Roots. I. Clarifying the Roles of Endodermal Barriers in the Salt Stress Response

**DOI:** 10.3389/fpls.2017.01326

**Published:** 2017-07-28

**Authors:** Kylie J. Foster, Stanley J. Miklavcic

**Affiliations:** Phenomics and Bioinformatics Research Centre, School of Information Technology and Mathematical Sciences, University of South Australia Mawson Lakes, SA, Australia

**Keywords:** Casparian strip, suberin lamellae, salt tolerance, apoplastic and symplastic transport, osmotic stress

## Abstract

In this paper, we present a detailed and comprehensive mathematical model of active and passive ion and water transport in plant roots. Two key features are the explicit consideration of the separate, but interconnected, apoplastic, and symplastic transport pathways for ions and water, and the inclusion of both active and passive ion transport mechanisms. The model is used to investigate the respective roles of the endodermal Casparian strip and suberin lamellae in the salt stress response of plant roots. While it is thought that these barriers influence different transport pathways, it has proven difficult to distinguish their separate functions experimentally. In particular, the specific role of the suberin lamellae has been unclear. A key finding based on our simulations was that the Casparian strip is essential in preventing excessive uptake of Na^+^ into the plant via apoplastic bypass, with a barrier efficiency that is reflected by a sharp gradient in the steady-state radial distribution of apoplastic Na^+^ across the barrier. Even more significantly, this function cannot be replaced by the action of membrane transporters. The simulations also demonstrated that the positive effect of the Casparian strip of controlling Na^+^ uptake, was somewhat offset by its contribution to the osmotic stress component: a more effective barrier increased the detrimental osmotic stress effect. In contrast, the suberin lamellae were found to play a relatively minor, even non-essential, role in the overall response to salt stress, with the presence of the suberin lamellae resulting in only a slight reduction in Na^+^ uptake. However, perhaps more significantly, the simulations identified a possible role of suberin lamellae in reducing plant energy requirements by acting as a physical barrier to preventing the passive leakage of Na^+^ into endodermal cells. The model results suggest that more and particular experimental attention should be paid to the properties of the Casparian strip when assessing the salt tolerance of different plant varieties and species. Indeed, the Casparian strip appears to be a more promising target for plant breeding and plant genetic engineering efforts than the suberin lamellae for the goal of improving salt tolerance.

## 1. Introduction

In this paper, we present and implement a detailed theoretical model of the uptake and transport of ions and water through a plant root that captures a large set of the anatomical and biophysical features that have been identified through experiments to play an important role in the salt stress response of plants. The model builds on our earlier efforts to quantify the passive transport of ions and water in a root (Foster and Miklavcic, 2013, 2014, 2016) and the active and passive transport of ions and water across the membranes of an individual plant cell (Foster and Miklavcic, 2015). The innovative approach presented in this paper combines these two critical aspects into a comprehensive model that features: explicit modeling of the apoplastic and symplastic transport of ions and water; active and passive membrane transport of ions and water across the tonoplast and plasma membranes of all cells in the root; the effect of the electric field influencing ion movement through the root; and, the individual effects of endodermal barriers. With the level of detail encompassed by this model we are able to quantify the relative influences of individual transporters as well as quantify the interactions between these features under different conditions. In this paper, we utilize the model to address some specific points of uncertainty on the respective roles played by the two dominant endodermal barriers.

The detrimental effects of salinity, particularly high levels of Na^+^ in the soil, manifest themselves in osmotic and ionic form. Osmotic stress is the more immediate challenge faced by plants, and is associated with a plant's reduced ability to take up water due to the higher osmotic pressure in saline soils. The slower action of ionic stress involves the accumulation of Na^+^ (and possibly Cl^−^) to toxic concentrations in plant tissues, especially in leaves (Munns and Tester, 2008). Since the first major anatomical study of plants, over 300 years ago by Grew (1965), it has been known that many plant species develop barriers in their root tissues. The two key barriers, namely the Casparian strip (CS) and suberin lamellae (SL), develop in the endodermis, and in some plant species also in the hypodermis (Enstone et al., 2002; Geldner, 2013). These barriers are now understood to restrict the transport of toxic ions (but also water) into root vascular tissues, which in turn limits the amount that enters leaves. Thus, these barriers play potentially significant roles in the salt stress response (Enstone et al., 2002).

The CS is a belt-like structure, consisting primarily of lignin (Naseer et al., 2012), that forms in the middle of the primary cell wall between adjacent endodermal cells (Barberon and Geldner, 2014; Franke, 2015). The CS blocks the non-selective, apoplastic transport of solutes and water, dividing the root into inner and outer regions, with the inner region containing the protected stele and xylem transpiration stream (Barberon et al., 2016). In contrast, the SL form on the inner surface of primary cell walls in the secondary stage of endodermal development. It is believed that the lamellae block the selective transport of ions and water across the plasma membranes of affected endodermal cells (Enstone et al., 2002; Geldner, 2013; Barberon and Geldner, 2014; Barberon et al., 2016), disrupting transport between the apoplastic and symplastic domains (as well as the transcellular transport pathway). On the other hand, the SL barrier does not disrupt transport via the plasmodesmata and hence would not interfere with the transport of ions and water *within* the symplastic pathway (Enstone et al., 2002; Geldner, 2013).

In general, there is a lack of clarity surrounding the differing roles of these endodermal barriers. One contributing factor is the often held belief that the CS is suberized (Krishnamurthy et al., 2009, 2011; Ranathunge and Schreiber, 2011). A second is the misconception that the SL acts as an apoplastic barrier (Das et al., 2015; Rossi et al., 2015). These views, which are in conflict with more recent understanding of the endodermal barrier composition and function, have led to some erroneous interpretations of experimental observations and flawed conclusions of the processes at work. In addition, conclusive experimental studies of individual barrier contributions have been hindered by the fact that SL develop only after the CS has already formed. Even the recent promising discovery of mutants displaying enhanced suberin deposition, *esb1* (Baxter et al., 2009; Hosmani et al., 2013) has not overcome this difficulty, as it has been observed that these mutants have both disrupted CSs as well as enhanced suberin deposition (Hosmani et al., 2013).

The role of the CS in preventing Na^+^ uptake is more clearly recognized than is that of the SL. However, the influence of the CS on water transport, particularly under salt stress, is not clear (Geldner, 2013). The evidence linking suberin deposition to salt stress includes enhanced suberin deposition in response to salt stress in rice (Krishnamurthy et al., 2009, 2011), *Arabidopsis thaliana* (Barberon et al., 2016), and castor bean (Schreiber et al., 2005), as well as the correlation between reduced suberin deposition in some *Arabidopsis* genotypes and reduced salt tolerance (Barberon et al., 2016). However, despite these experimental observations, how the SL contribute to the salt stress response is still not clear.

To clarify the respective roles of the CS and SL in the salt stress response of plant roots we here apply our comprehensive model, which is well-suited to the task as it includes the explicit separation of the symplastic and apoplastic pathways, allowing the individual effects of the SL and CS to be explored. With the aim of presenting a clear and quantitative picture we consider the passive and active membrane transport of those ions that are most often implicated or involved in the salt stress response of plants (i.e., Na^+^, Cl^−^, K^+^, and H^+^). Water transport is also included, allowing us to investigate the osmotic contributions to salt stress.

In the next section (Section 2), the physical model and the key assumptions are described. The corresponding mass-balance equations for water and ions are outlined in Section 3, along with an explanation of the numerical solution method, the boundary conditions, and the choice of parameters. Further model details, including the water flow and ion flux equations, as well as an additional description of the parameters, are provided in the Supplementary Material. Section 4 presents results of simulations which explore the separate influences of the CS and SL on ion and water transport, particularly in response to salt stress. The implications of these results are discussed in Section 5. The paper concludes in Section 6 with a summary and a comment on possible future work.

## 2. Model root geometry and transport mechanisms

**Figure 1:**
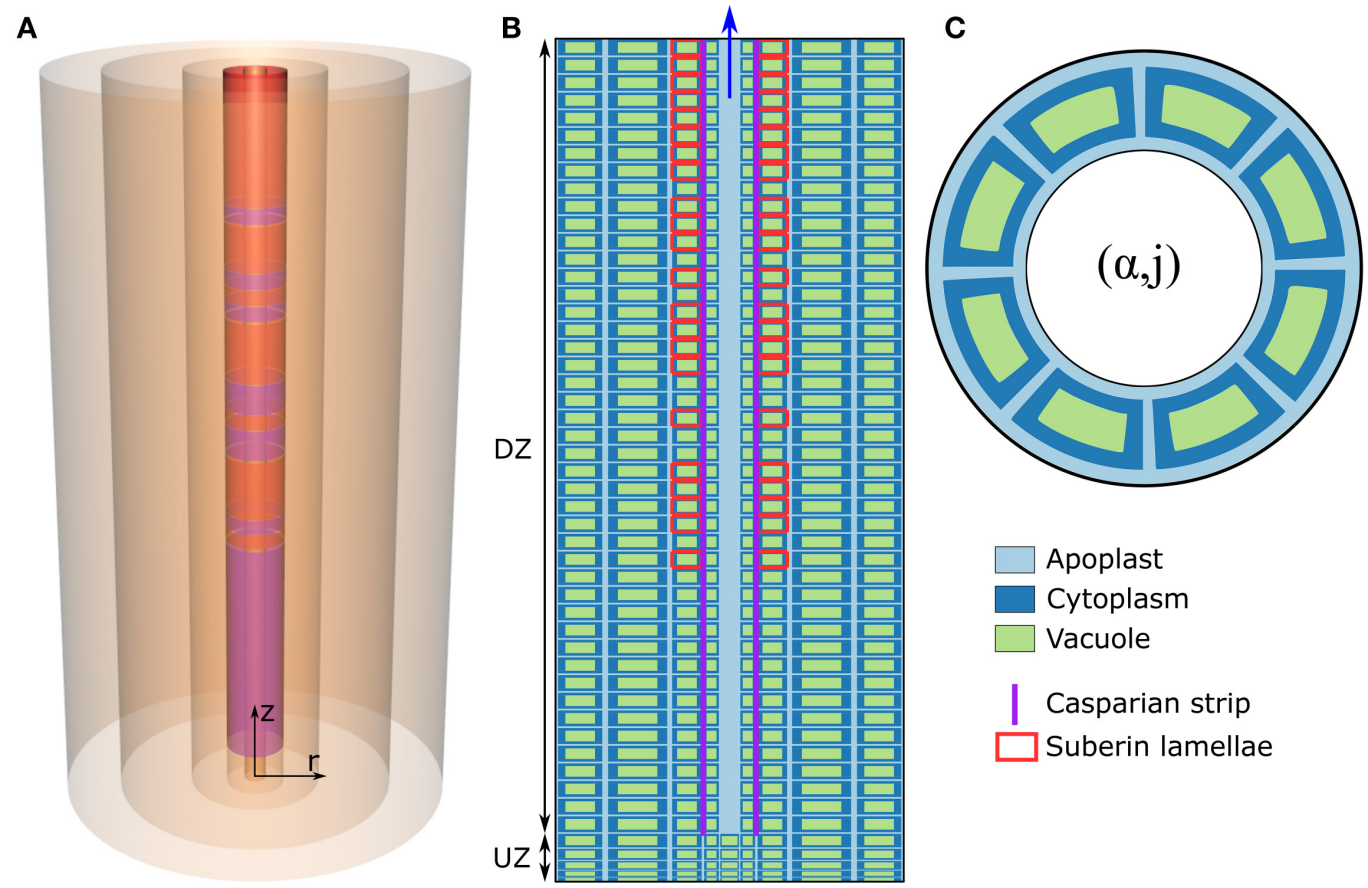
**(A)** Schematic of the cylindrical model root, showing the radial discretization and the endodermal barriers. The purple (red) cylinders represent the CS (SL). **(B)** Schematic longitudinal cross-section of the root. Different cell compartments are highlighted by different colors: apoplast (light blue); cytoplasms (dark blue); the vacuoles (green). For clarity, these compartments are not drawn to scale and the *z* and *r* axes are scaled differently. The extent of the different developmental zones—the differentiation zone (DZ) and undifferentiated zone (UZ)—are indicated by arrows. Purple (red) lines represent the location of the CS (SL). **(C)** Top view of the root showing the angular distribution of cells in a given (α, *j*) element.

The model of ion and water transport in a plant root presented here is based on the models described in previous works (Foster and Miklavcic, 2013, 2014, 2016), particularly the model presented in Foster and Miklavcic (2016). The root structure (Figure 1) is modeled as a discrete set of co-axial, annular cylinders, with each cylinder containing cells which represent an individual tissue type. Limiting the numerical implementation to model an *Arabidopsis* primary root, five distinct, single-cell, tissue types are considered: the epidermis (α = 1), the cortex (α = 2), the endodermis (α = 3), the pericycle (α = 4), and the xylem (α = 5). Note that other plant species can be represented by increasing either the size or number of tissue regions. The axial dimension of the root is discretized into *M* sections (*j* = 1, …, *M*), with each discrete section representing a single plant cell layer. For convenience, the height of these cell layers is assumed uniform across all tissue types, but variable between developmental zones. For all simulations, the total root length is *M* = 50 cell layers long. As in Foster and Miklavcic (2016), the root is further partitioned axially into two developmental zones: a zone of undifferentiated cells extending longitudinally from the root tip (the undifferentiated zone, UZ), and a differentiation zone (DZ) of mature cells (see Figure 1B). The distribution of membrane transport proteins in cells in the undifferentiated zone models the function of cells of both the meristematic and elongation zones, which would be infeasible to include explicitly given the small number of axial sections considered here. As done previously (Foster and Miklavcic, 2016), the two root developmental zones are distinguished by axial variations in transport parameters as well as cell height.

The present model extends the authors' earlier models (Foster and Miklavcic, 2013, 2014, 2016) in a number of important ways.

First, each tissue, and hence each (α, *j*) element, contains a fixed number of cells distributed angularly around the root axis (see Figure 1C) according to the cellular organization of tissues within *Arabidopsis* primary roots (see Table S3 in the Supplementary Material). Second, each discrete (α, *j*) element (with the exception of mature xylem elements) consists of an apoplastic, a cytoplasmic, and a vacuolar compartment. Discrete elements representing functional xylem (i.e., xylem elements in the DZ) consist only of an apoplast compartment as this tissue is assumed not to contain living cells. In this paper, the term “element” will be used to refer to a discrete (α, *j*) component of the root which contains all of the above mentioned cell regions (see Figure 1C), while “compartment” is a generic reference to the sub-components contained within each (α, *j*) element (i.e., the apoplastic, cytoplasmic, and vacuolar compartments). Note that, with the multiple identical cells distributed angularly, each (α, *j*) element contains multiple, but identical, cytoplasmic and vacuolar compartments but only a single apoplast compartment, which is assumed to be the space between the cells in that element (see Figure 1C).

A third significant development is the explicit consideration of ion and water transport through the apoplastic and symplastic pathways, as well as transport between the two pathways. The symplastic pathway includes passive transport of ions and water through plasmodesmata. A fourth new feature is the inclusion of both active and passive ion transport across tonoplast and plasma membranes via membrane transport proteins as described in Foster and Miklavcic (2015). Transport parameters are assumed uniform within each cell compartment, but not necessarily across tissue types or developmental zones. Given the assumption of axisymmetry and the consequent fact that the cells *within* each (α, *j*) annular element are identical, the explicit transport of ions and water between the cells within a given (α, *j*) element is not required.

As in Foster and Miklavcic (2013, 2014, 2015, 2016), ion and water transport are modeled interdependently thus allowing both convection and the osmotic effects of salt stress to be studied. Radial and axial apoplastic water flow is driven by hydraulic pressure gradients in all tissue regions, simulating the effects of transpiration. The flow of water into cell cytoplasms, both between the apoplast and symplast and via the plasmodesmata, is also driven by hydraulic pressure gradients, as well as osmotic pressure gradients. Within each cell, water flow across the tonoplast is due to osmotic pressure differentials between the cytoplasm and the vacuole.

**Figure 2:**
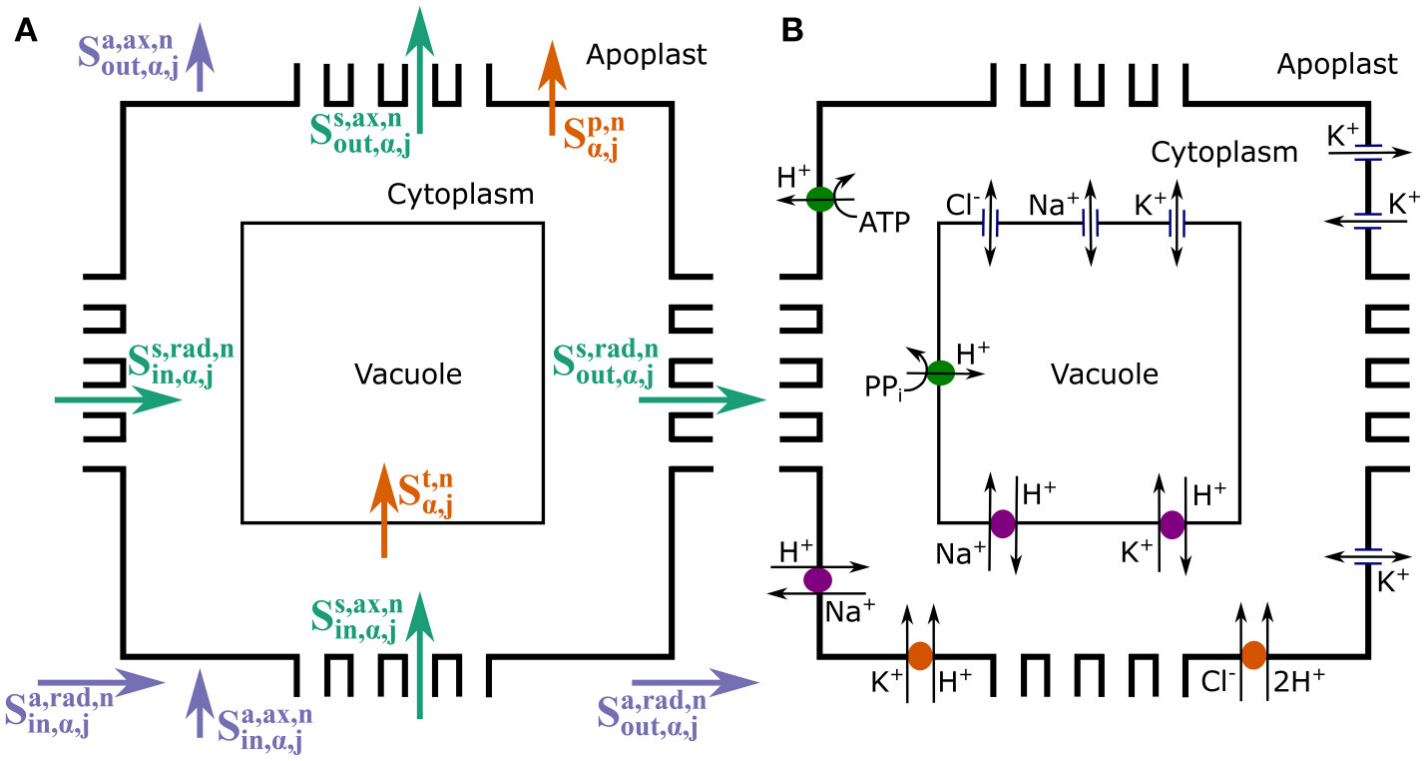
Schematic longitudinal cross-sections of an (α, *j*) element, showing the three compartments (apoplast, cytoplasm, and vacuole) and the different ion fluxes included in the model. The cross-sections illustrate: **(A)** the symplastic (green), apoplastic (purple), and transmembrane (orange) ion fluxes; and **(B)** the membrane transport proteins (pumps, antiporters, symporters, and channels) present on the tonoplast and plasma membranes responsible for the transmembrane fluxes. For reaction processes see Figure S1.

As already mentioned, four mobile ion species are explicitly considered: *n* = Na^+^, Cl^−^, K^+^, and H^+^, which are either the ions most often implicated in salt stress or those directly affected. Figure 2 summarizes the types of solute transport considered in the model. Radial and axial transport of ions through the apoplast ($S_{in/out,\alpha ,j}^{a,rad,n}$ and $S_{in/out,\alpha ,j}^{a,ax,n}$, respectively) and within the symplast, via plasmodesmata ($S_{in/out,\alpha ,j}^{s,rad,n}$ and $S_{in/out,\alpha ,j}^{s,ax,n}$, respectively) is passive and is assumed to be driven by electrochemical diffusion and convection. On the other hand, both active and passive processes are involved in the transport of ions across the plasma membrane and the tonoplast ($S_{\alpha ,j}^{p,n}$ and $S_{\alpha ,j}^{t,n}$, respectively), via the membrane transport proteins studied in Foster and Miklavcic (2015). As described in Foster and Miklavcic (2015), the plasma membrane transport proteins include: H^+^ pumps, which power Na^+^/H^+^ antiporters, K^+^/H^+^ symporters, and Cl^−^/H^+^ symporters; inward rectifying channels (IRCs) and outward rectifying channels (ORCs), which passively transport K^+^; as well as voltage insensitive channels (VICs), which passively transport Na^+^, K^+^, and Cl^−^. The transport proteins acting on the tonoplast membrane are: H^+^ pumps, which power Na^+^/H^+^ and K^+^/H^+^ antiporters; as well as VICs. A detailed description of these membrane transport processes, including justifications for the model assumptions, is provided in Foster and Miklavcic (2015) and its associated Supplementary Data. A synopsis of this information is included in the Supplementary Material accompanying the present paper. Diffusion and convection effects *within* the cytoplasm and within the vacuole of each cell are not included as these would require an extension to a multiscale level. Many of the spatially dependent effects would be dominated by steep gradients very close to membrane surfaces that must still be matched to relatively slowly varying functional behavior in interior regions of the compartments. The working assumption of constant compartment values is discussed further in Section 5.

As mentioned previously (Foster and Miklavcic, 2013), the suggestion that the stele of the root for a plant such as *Arabidopsis* is axisymmetric is simplistic. In reality, the center of the root is a more complex arrangement consisting of protoxylem, metaxylem, phloem, xylem parenchyma, and phloem parenchyma cells (Dolan et al., 1993). However, as the function of the vascular cylinder is assumed here to be dominated by the xylem, the simplified treatment of the stele nevertheless captures the key processes that are of interest in this study. The assumptions thus lead to the α = 5 elements in the DZ being considered as consisting entirely of a cylindrical apoplast. As a result, the cells in tissue region α = 4 (labeled pericycle) also encompass many of the functions of xylem parenchyma cells in terms of the loading and unloading of ions and water to and from the xylem.

The CS is assumed to either completely or partially block the *apoplastic* transport of water and ions (Geldner, 2013) at the endodermis-pericycle interface. In contrast, the SL are assumed to block the transport of ions and water across the *plasma membrane* of affected endodermal cells, without impacting on transport via plasmodesmata (Eshel and Beeckman, 2013; Geldner, 2013).

The volumes of each cell and of each (α, *j*) element are assumed constant. As a result, the volume of the apoplast is also constant. However, the vacuole volume is free to vary in response to osmotically driven water flows. Hence, the vacuole volumes, tonoplast surface areas, and cytoplasm volumes will vary over time and across the root. This degree of volumetric freedom allows the osmotic component of salt stress to be investigated. A more detailed description of the root geometry and motivation for the various assumptions is provided in Section S.3 of the Supplementary Material.

Plant cell walls contain fixed anionic groups; in particular, carboxylic acid groups are present due to the existence of pectins in the cell walls (Hodson and Bryant, 2012). These fixed negatively charged groups attract and bind cations while repelling anions and thus can significantly influence the apoplastic transport pathway. The effects of these fixed anions in the apoplast are included through separate binding reactions for each individual cation species and their influence is included in the calculation of the electric potential.

As in Foster and Miklavcic (2015), buffering of H^+^ is included in the cytoplasmic and vacuolar compartments and is modeled by assuming constant buffering capacities in these compartments. The dissociated buffering anions and undissociated buffering compounds are assumed to be immobile across the plasmodesmata and cell membranes. These buffering components contribute to the osmotic pressure in the cytoplasm and vacuole. In addition, the buffering anions contribute to the electric potential.

With a focus on charged solutes, ion fluxes will depend on the electric potential differences between the different compartments. Based on findings in Foster and Miklavcic (2013, 2014, 2016) and in correspondence with the model adopted in Foster and Miklavcic (2015), the electric potentials across the root can be approximately determined by assuming electroneutrality in each apoplastic, cytoplasmic and vacuolar compartment. The details of the calculation of the electric potential values are provided in Section S.1.6 of the Supplementary Material. In brief, the assumption of electroneutrality, combined with the expressions for the ion fluxes, leads to a set of zero net current conditions for the apoplast, symplast, and tonoplast for each (α, *j*). This system of non-linear equations is solved to find the electric potential in each compartment for all (α, *j*).

## 3. Mathematical formulation

### 3.1. Water transport

The mass-balance equation for the apoplastic fluid in each (α, *j*) element is given by,

(1) $$\begin{array}{r} \frac{dw_{\alpha ,j}^{a}}{dt}=\rho Q_{in,\alpha ,j}^{a,rad}-\rho Q_{out,\alpha ,j}^{a,rad}+\rho Q_{in,\alpha ,j}^{a,ax}-\rho Q_{out,\alpha ,j}^{a,ax}\\ +\;N_{\alpha ,j}^{cells}\rho Q_{\alpha ,j}^{p}, \end{array}$$

where *w* is the mass of the solution, ρ is the solution density, $Q_{in,\alpha ,j}^{a,rad}$ ($Q_{out,\alpha ,j}^{a,rad}$) is the *radial* volume flow rate into (out of) the apoplast of element (α, *j*) from the apoplast of the surrounding tissues, $Q_{in,\alpha ,j}^{a,ax}$ ($Q_{out,\alpha ,j}^{a,ax}$) is the *axial* volume flow rate into (out of) the apoplast of element (α, *j*) from the apoplast of the surrounding tissues, $N_{\alpha ,j}^{cells}$ is the number of cells in element (α, *j*), $Q_{\alpha ,j}^{p}$ is the volume flow rate into the apoplast across the plasma membrane of each individual cell in element (α, *j*). *Q*^*p*^ represents transport between the symplastic and apoplastic transport pathways and hence this term is only present in model elements where living cells are present (i.e., *Q*^*p*^ = 0 in the mature xylem). As in our previous work (Foster and Miklavcic, 2013), the assumption of point particles implies that the solutes do not contribute to volume.

For a constant solution density, Equation (1) can be reduced to a conservation equation for the apoplastic water volume in each (α, *j*) element, $V_{\alpha ,j}^{a}$,

(2) $$\begin{array}{r} \frac{dV_{\alpha ,j}^{a}}{dt}=Q_{in,\alpha ,j}^{a,rad}-Q_{out,\alpha ,j}^{a,rad}+Q_{in,\alpha ,j}^{a,ax}-Q_{out,\alpha ,j}^{a,ax}+N_{\alpha ,j}^{cells}Q_{\alpha ,j}^{p}. \end{array}$$

Assuming that each (α, *j*) element is full of liquid and that the cells in each element are of fixed size, leads to a constant apoplastic volume. Therefore, $dV_{\alpha ,j}^{a}/dt=0$ and Equation (2) simplifies to the condition,

(3) $$\begin{array}{r} Q_{in,\alpha ,j}^{a,rad}-Q_{out,\alpha ,j}^{a,rad}+Q_{in,\alpha ,j}^{a,ax}-Q_{out,\alpha ,j}^{a,ax}+N_{\alpha ,j}^{cells}Q_{\alpha ,j}^{p}=0. \end{array}$$

The mass-balance equation for the fluid in the vacuole of each cell in each (α, *j*) element similarly reduces to a conservation of volume condition,

(4) $$\begin{array}{r} \frac{dV_{\alpha ,j}^{v}}{dt}=Q_{\alpha ,j}^{t}, \end{array}$$

where $V_{\alpha ,j}^{v}$ is the volume of the vacuole of each cell in each (α, *j*) element and $Q_{\alpha ,j}^{t}$ is the volume flow rate across the tonoplast into the vacuole of each cell. Since the functional portion of the xylem is assumed to not contain any living cells, Equation (4) applies only to all other tissue regions.

Similarly, the conservation condition for the fluid in the cytoplasm of each cell in each (α, *j*) element is given by,

(5) $$\begin{array}{r} \frac{dV_{\alpha ,j}^{c}}{dt}=\frac{1}{N_{\alpha ,j}^{cells}}\left(Q_{in,\alpha ,j}^{s,rad}-Q_{out,\alpha ,j}^{s,rad}+Q_{in,\alpha ,j}^{s,ax}-Q_{out,\alpha ,j}^{s,ax}\right)\\ -Q_{\alpha ,j}^{t}-Q_{\alpha ,j}^{p}, \end{array}$$

where $V_{\alpha ,j}^{c}$ is the volume of the cytoplasm of each individual cell in each (α, *j*) element and the *Q*^*s*^ terms refer to symplastic water flow rates. Specifically, $Q_{in,\alpha ,j}^{s,rad}$ ($Q_{out,\alpha ,j}^{s,rad}$) is the total radial volume flow rate into (out of) the cytoplasms of all of the cells in a given annular (α, *j*) element via plasmodesmata from the cytoplasms of radially adjoining cells, and $Q_{in,\alpha ,j}^{s,ax}$ ($Q_{out,\alpha ,j}^{s,ax}$) is the total axial volume flow rate into (out of) the cytoplasms of all of the cells in a given annular (α, *j*) element via plasmodesmata from the cytoplasms of axially adjoining cells. Assuming that the overall size of each cell is constant (due to cell wall constraints), any changes in the vacuole volume must result in corresponding changes in the cytoplasm volume,

(6) $$\begin{array}{r} \frac{dV_{\alpha ,j}^{c}}{dt}=-\frac{dV_{\alpha ,j}^{v}}{dt}. \end{array}$$

Using Equation (4),

(7) $$\begin{array}{r} \frac{dV_{\alpha ,j}^{c}}{dt}=-Q_{\alpha ,j}^{t}. \end{array}$$

Combining Equations (5) and (7), the assumptions of variable vacuole volume but fixed total cell volume lead to the condition,

(8) $$\begin{array}{r} Q_{in,\alpha ,j}^{s,rad}-Q_{out,\alpha ,j}^{s,rad}+Q_{in,\alpha ,j}^{s,ax}-Q_{out,\alpha ,j}^{s,ax}-N_{\alpha ,j}^{cells}Q_{\alpha ,j}^{p}=0. \end{array}$$

Equations (3) and (8) represent a system of linear equations which are solved simultaneously to determine the hydraulic pressure distribution throughout the apoplastic and cytoplasmic compartments of the root.

The details of the water flow rate calculations are provided in the Supplementary Material (Section S.1.1).

### 3.2. Solute transport

The mole balance equation for mobile ions, *n*, (where, *n* = Na^+^, Cl^−^, K^+^, H^+^) in the apoplastic compartment of each (α, *j*) element is given by,

(9) $$\begin{array}{rc} \frac{d\left(V_{\alpha ,j}^{a}C_{\alpha ,j}^{a,n}\right)}{dt}= & S_{in,\alpha ,j}^{a,rad,n}-S_{out,\alpha ,j}^{a,rad,n}+S_{in,\alpha ,j}^{a,ax,n}-S_{out,\alpha ,j}^{a,ax,n}+N_{\alpha ,j}^{cells}S_{\alpha ,j}^{p,n}\\ +R_{\alpha ,j}^{bind,n}, \end{array}$$

where $C_{\alpha ,j}^{a,n}$ is the concentration of species *n* in the apoplast of element (α, *j*), $S_{in,\alpha ,j}^{a,rad,n}$ ($S_{out,\alpha ,j}^{a,rad,n}$) is the *radial* flux of solute *n* into (out of) the apoplast of element (α, *j*) from the apoplast of the surrounding tissues, $S_{in,\alpha ,j}^{a,ax,n}$ ($S_{out,\alpha ,j}^{a,ax,n}$) is the *axial* flux of solute *n* into (out of) the apoplast of element (α, *j*) from the apoplast of the surrounding tissues, and $R_{\alpha ,j}^{bind,n}$ is the net rate of ion dissociation from the binding reaction with fixed negative groups which are present in the apoplast. The $S_{\alpha ,j}^{p,n}$ function represents the net flux of species *n* from the cytoplasm to the apoplast across the plasma membrane of each *individual* cell in the element (α, *j*) via membrane transport proteins,

(10) $$\begin{array}{r} S_{\alpha ,j}^{p,n}=\{\begin{array}{rr} J_{H}^{A_{a}}+J_{H}^{XNa_{a}}+J_{H}^{SK_{a}}+J_{H}^{SCl_{a}} & \text{if }n={\text{H}}^{+},\\ J_{Na}^{C_{a}}+J_{Na}^{XNa_{a}} & \text{if }n={\text{Na}}^{+},\\ J_{K}^{C_{a}}+J_{K}^{ORC_{a}}+J_{K}^{IRC_{a}}+J_{K}^{S_{a}} & \text{if }n={\text{K}}^{+},\\ J_{Cl}^{C_{a}}+J_{Cl}^{SCl_{a}} & \text{if }n={\text{Cl}}^{-}. \end{array} \end{array}$$

Each *J* in Equation (10) represents the ion flux through each different type of plasma membrane transport protein, with a positive *J* referring to flux out of the cytoplasm. The subscripts denote the relevant ion (H^+^, Na^+^, K^+^, Cl^−^), while the superscripts refer to the plasma membrane transport protein carrying the flux: *A*_*a*_ refers to the H^+^ pump, *XNa*_*a*_ the Na^+^/H^+^ antiporter, *SK*_*a*_ the K^+^/H^+^ symporter, *SCl*_*a*_ the Cl^−^/H^+^ symporter, *C*_*a*_ the VICs, *ORC*_*a*_ the outward rectifying channels and *IRC*_*a*_ the IRCs.

In addition to the mobile ions, the apoplastic compartments also contain cations which are bound to fixed negative charges (as outlined in Section 2). The mole balance equation for these bound cations in the apoplastic compartment of each (α, *j*) element is given by,

(11) $$\begin{array}{r} \frac{d\left(V_{\alpha ,j}^{a}C_{\alpha ,j}^{a,nB}\right)}{dt}=-R_{\alpha ,j}^{bind,n}, \end{array}$$

where $C_{\alpha ,j}^{a,nB}$ refers to the concentration of bound cations (*HB*, *NaB*, *KB*).

The conservation equation for mobile ions in the cytoplasmic compartment of each cell in each annular (α, *j*) element is given by,

(12) $$\begin{array}{rc} \frac{d}{dt}\left(V_{\alpha ,j}^{c}C_{\alpha ,j}^{c,n}\right) & =\frac{1}{N_{\alpha ,j}^{cells}}\left(S_{in,\alpha ,j}^{s,rad,n}-S_{out,\alpha ,j}^{s,rad,n}+S_{in,\alpha ,j}^{s,ax,n}-S_{out,\alpha ,j}^{s,ax,n}\right)\\ \;-S_{\alpha ,j}^{pm,n}-S_{\alpha ,j}^{t,n}, \end{array}$$

where $C_{\alpha ,j}^{c,n}$, is the concentration of species *n* in the cytoplasm of each *individual*, identical cell in the annular element (α, *j*), $S_{in,\alpha ,j}^{s,rad,n}$ ($S_{out,\alpha ,j}^{s,rad,n}$) is the total radial flux of ion *n* into (out of) the cytoplasms of all of the cells in (α, *j*) via plasmodesmata and $S_{in,\alpha ,j}^{s,ax,n}$ ($S_{out,\alpha ,j}^{s,ax,n}$) is the total axial flux of ion *n* into (out of) the cytoplasms of all of the cells in (α, *j*) via plasmodesmata. $S_{\alpha ,j}^{t,n}$ represents the net flux of ion *n* across the tonoplast of each individual cell via membrane transport proteins,

(13) $$\begin{array}{r} S_{\alpha ,j}^{t,n}=\{\begin{array}{rr} J_{H}^{A_{v}}+J_{H}^{XNa_{v}}+J_{H}^{XK_{v}} & \text{if }n=H^{+},\\ J_{Na}^{C_{v}}+J_{Na}^{XNa_{v}} & \text{if }n=Na^{+},\\ J_{K}^{C_{v}}+J_{K}^{XK_{v}} & \text{if }n=K^{+},\\ J_{Cl}^{C_{v}} & \text{if }n=Cl^{-}. \end{array} \end{array}$$

Each *J* in Equation (13) represents an ion flux through a different tonoplast membrane transport protein, with each *J* positive out of the cytoplasm (into the vacuole). Again, the subscripts refer to the ion being transported (H^+^, Na^+^, K^+^, Cl^−^), while the superscripts refer to the type of tonoplast transport protein through which the flux is occurring: *A*_*v*_ refers to the H^+^ pump, *XNa*_*v*_ the Na^+^/H^+^ antiporter, *XK*_*v*_ the K^+^/H^+^ antiporter, and *C*_*v*_ the various channels.

Similarly, the conservation equation for mobile ions in the vacuolar compartment of each cell in each annular (α, *j*) element is given by,

(14) $$\begin{array}{r} \frac{d}{dt}\left(V_{\alpha ,j}^{v}C_{\alpha ,j}^{v,n}\right)=S_{\alpha ,j}^{t,n}, \end{array}$$

where $C_{\alpha ,j}^{v,n}$ is the concentration of species *n* in the vacuole of each individual, identical cell in element (α, *j*).

Further details relating to the computation of solute transport processes are provided in the Supplementary Material. Specifically, the apoplastic and symplastic ion flux calculations are provided in Section S.1.2, while the formulation of the equations for the transmembrane fluxes (Equations 10 and 13) are provided in Section S.1.3. The equations for the transmembrane fluxes are as described previously in Foster and Miklavcic (2015), although in this paper the membrane transport parameters can vary with location and hence are dependent on (α, *j*). A description of the modeled binding processes in the apoplast is provided in Section S.1.4. The expressions for the rate of change of buffering anions and free H^+^ concentrations are provided in Section S.1.5.

### 3.3. Computational details

The non-linear, coupled, system of ODEs with respect to time represented by Equations (4), (9), (11), (12), (14), and the buffering conservation equations (see Section S.1.5), combined with the non-linear system of algebraic equations represented by Equations (S59)–(S61) and the linear system of algebraic equations represented by Equations (3) and (8) were solved numerically in MATLAB. The system of ODEs and the non-linear zero net current constraint (Equations S59–S61) were solved using the ode15s package which is capable of solving such a system of differential algebraic equations. The hydraulic pressure was determined at each time step by solving the linear system of equations represented by Equations (3) and (8). Due to the large number of variables solved for in this system of equations (a total of 101 M + 56 equations in 101 M + 56 unknowns), the model root was assumed to consist of M = 50 elements in the axial direction, allowing us to explore the effects of variations in transport properties along the length of the root.

As in our single cell model (Foster and Miklavcic, 2015), a two-stage simulation process was used to mimic salt stress experiments. In the first, pre-salt stage all ions except Na^+^ were present in the external medium and root; the external medium featured a pH of 5.9 and a KCl concentration of 10 mM (note that [K^+^] in the external medium was very slightly <10 mM to compensate for the extra positive charge due to the pH, and hence to ensure electroneutrality). The initial state of this stage was assumed electroneutral and the MATLAB package fsolve was used to solve the non-linear system of algebraic equations represented by Equations (S59)–(S61), with the solution used to determine a consistent set of initial conditions for the electric potentials. The steady-state results of this first stage simulation were used as initial conditions for the second, salt stress stage (with fsolve again used to determine consistent initial conditions for the electric potentials). At the start of this salt stress stage 100 mM of NaCl was introduced in the external medium. This two-stage process, representative of many salt stress experiments, allows for a transient response study.

The boundary conditions for this model are very similar to those imposed in previous work (Foster and Miklavcic, 2016), with a few additional conditions to cover transport in the symplast. As in our earlier studies (Foster and Miklavcic, 2013, 2014, 2016) there is a linear, hydrostatic, pressure gradient in the external medium and constant bulk concentrations of ions.

There is zero apoplastic and symplastic ion and water flow across the bottom boundary of the root, reflecting the impermeable nature of the root cap. The boundary conditions at the top boundary of the root are based on those adopted in Foster and Miklavcic (2016), which assumed that flow of water and ions occurred across only the xylem boundary. Hence, in this model there is zero symplastic and apoplastic ion and water flow across the top boundary of the root for all tissue regions except the xylem. Since the functional xylem does not contain a symplastic component, there is in no symplastic transport across the top boundary of the root. It is assumed that there is a constant apoplastic hydraulic pressure (*P*_*b*_) and zero apoplastic concentration gradients across the boundary at the top of the xylem.

The boundary conditions at the top of the root represent its connection to the rest of the plant. In particular, the imposed constant, *P*_*b*_, represents the effect of plant transpiration and is the driving force for root water flow. The effects of varying *P*_*b*_ (more negative/positive *P*_*b*_ equates to higher/lower transpiration) will be explored in Section 4. That *P*_*b*_ is assigned a constant value in a given simulation implies the absence of a self-consistent link between transpiration, root permeability and soil osmotic pressure, a link which has been observed in experiments (Rodriguez et al., 1997; Maggio et al., 2007). A possible future extension of the model would be to include the interplay between leaf, stem and root transport processes to model feedback effects on leaf transpiration. For the present, we apply a constant apoplastic hydraulic pressure at the top of the root, but allow the pressure elsewhere to respond self-consistently to hydraulic and osmotic conditions.

There are two additional boundary conditions applied to the symplast. Firstly, there is zero symplastic flow of water and ions across the external medium-epidermis interface; this represents the absence of plasmodesmata at this boundary. Secondly, there is zero symplastic flow of water and ions across the pericycle-functional xylem interface, which reflects the lack of plasmodesmatal connections to the functional, purely apoplastic, non-living xylem.

To assist the solver, particularly due to comparatively small vacuolar volumes, all variables were scaled. The concentrations of Na^+^, K^+^, and Cl^−^ were scaled by the relevant concentrations in the external medium, with Cl^−^ concentrations scaled using the pre-salt external medium concentration. The buffering anion concentrations for each cell compartment were scaled by the average of the initial buffering anion concentrations in each compartment, and similarly the vacuolar volumes, $V_{\alpha ,j}^{v}$, were scaled by the average of the initial vacuolar volumes in all of the cells. All electric potentials were scaled by $\frac{R_{g}T}{F}$, where *R*_*g*_ is the Universal gas constant (8.314 J mol^−1^ K^−1^), *T* is the temperature and *F* is Faraday's constant (96 485 C mol^−1^).

### 3.4. Parameter selection: membrane transport protein distributions and endodermal barriers

All membrane transport parameter values implemented in this model were based on values (particularly the salt tolerant values) adopted in Foster and Miklavcic (2015) (summarized in Table S2 in the Supplementary Material). Note that, where relevant, the membrane transport parameters (${\text{P}}_{a}^{VIC}$, ${\text{N}}_{a}^{A}$, ${\text{k}}_{a}^{XNa}$, etc.) are expressed per unit of initial membrane area in recognition of the ability of larger cell membranes to carry a greater number of transport proteins. In addition, due to the compartmental nature of the model, all transport proteins are assumed to be uniformly distributed across the relevant cell membranes.

It has been experimentally observed that the genes associated with many of the membrane transport proteins relevant to salt stress are expressed non-uniformly across the different root tissues and root developmental zones. As a consequence, the transport parameter values adopted here were adjusted in the transport scenarios in Section 4.2 to represent the known spatial distribution patterns of gene expression, with functional transport proteins only present in the relevant tissues and developmental regions. The known and assumed pattern of distribution of K^+^ transport proteins is provided in Table 1, while the VICs, which transport both cations and anions, are assumed to be present in all tissue types (representing a base level of leakage out of the cells and vacuoles). Cl^−^/H^+^ symporters are assumed to be present in the same tissues as K^+^/H^+^ symporters. Tonoplast Na^+^/H^+^ antiporters are assumed to be present in all cells in the DZ and are absent from all cells in the UZ (Shi and Zhu, 2002). Plasma membrane Na^+^/H^+^ antiporter genes have been identified to be preferentially expressed in the xylem parenchyma and pericycle in the DZ, as well as in the epidermis in the root tip (Shi et al., 2002). In addition, flux measurements conducted by Shabala et al. (2005) suggest that plasma membrane Na^+^/H^+^ antiporters are functional along the full length of the root. Hence, we assume the level of plasma membrane antiporter activity (represented by ${\text{k}}_{\alpha ,j}^{XNa_{a}}$) is equal to the fitted value obtained in Foster and Miklavcic (2015) in the epidermis for the full length of the root and in the pericycle in the DZ. In addition, a lower level of activity (${\text{k}}_{\alpha ,j}^{XNa_{a}}$ reduced by one order of magnitude) is assumed for the remaining tissues to represent the experimentally observed background level of plasma membrane antiporter expression in all root tissues (Shi et al., 2002). Note that there is some uncertainty around this distribution of plasma membrane Na^+^/H^+^ antiporters, which will be explored in a future study. H^+^ pumps are assumed to be present in all cell types in which secondary active transporters are present, as these transporters require the operation of H^+^ pumps to be functional.

**Table 1:** Summary of the distribution of plasma membrane K^+^ membrane transport proteins and associated literature references.

**Transport protein**	**Spatial distribution**	**Source**
IRCs	DZ: epidermis, cortex, endodermis	Desbrosses et al., 2003
	UZ: epidermis	Lagarde et al., 1996
ORCs	DZ: epidermis, pericycle	Gaymard et al., 1998
		Ivashikina et al., 2001
K^+^/H^+^ symporters	DZ: epidermis, pericycle	Gierth et al., 2005

*IRCs, inward rectifying channels; ORCs, outward rectifying channels; DZ, differentiation zone; UZ, undifferentiated zone*.

The effects of the endodermal barriers were explored by changing relevant transport parameters in the endodermal elements. A completely impermeable CS was simulated by reducing the apoplastic ion permeabilities and water permeability to zero at the endodermis-pericycle interface of the affected cells (i.e., $k_{\alpha =4,j_{CS}}^{a,rad,n}=0$ and $L_{p:\alpha =4,j_{CS}}^{a,rad}=0$, where *j*_*CS*_ refers to all *j* for which the CS is present). This set of parameters simulates the complete blockage, at the endodermal-pericycle interface, of water and ion transport through the apoplast. In a similar manner, the effect of a partially permeable, or leaky, CS was simulated by assuming values of $k_{\alpha =4,j_{CS}}^{a,rad,n}$ and $L_{p:\alpha =4,j_{CS}}^{a,rad}$ intermediate between zero and their respective values in the absence of a CS. Note that while the CS is represented by altering the apoplastic transport properties at the endodermal-pericycle interface, in reality the CS develops in the apoplast at the center of the endodermis (Geldner, 2013). Hence, the effect of simulating the CS by instead altering the apoplastic transport parameters at the cortical-endodermal interface was also investigated (see Section S.4 in the Supplementary Material).

The SL interferes with the uptake of water and ions across the plasma membranes of endodermal cells (Geldner, 2013). Hence, the action of a completely impermeable SL was modeled by turning off all plasma membrane transport proteins on affected endodermal cells (i.e., by setting ${\text{N}}_{\alpha =3,j_{SL}}^{A_{a}}=0$, ${\text{k}}_{\alpha =3,j_{SL}}^{XNa_{a}}=0$, ${\text{P}}_{\alpha =3,j_{SL}}^{VIC_{a}}=0$, ${\text{P}}_{\alpha =3,j_{SL}}^{Cl_{a}}=0$, $P_{\alpha =3,j_{SL}}^{IRC_{a}}=0$, $P_{\alpha =3,j_{SL}}^{ORC_{a}}=0$, $N_{\alpha =3,j_{SL}}^{SK_{a}}=0$, and $N_{\alpha =3,j_{SL}}^{SCl_{a}}=0$) and setting the plasma membrane water permeability to zero ($L_{p:\alpha =3,j_{SL}}^{p}=0$, where *j*_*SL*_ refers to all *j* for which SL are present). The DZ was assumed to start at *j* = 6, the CS was assumed to begin at the start of the DZ (*j*_*CS*_ = 6, …, 50) and the SL was assumed to be present from *j* = 19. The role of passage cells was also explored. Their distribution, where relevant, was based for illustrative purposes on the distribution introduced in Foster and Miklavcic (2016), as illustrated in Figures 1A,B.

The remaining parameter values and their sources are discussed in Section S.2 and listed in Table S1 in the Supplementary Material.

## 4. Results

Not surprisingly, in solving the governing system of equations presented in Section 3, an enormous wealth of information becomes available for scrutiny. Features of greatest interest include the spatio-temporal variation of: apoplastic, cytoplasmic and vacuolar concentrations of mobile ions (H^+^, Na^+^, K^+^, Cl^−^, and B^−^); apoplastic, symplastic, and transmembrane ion and water fluxes; hydraulic and osmotic pressures; and electric potentials. At the same time, the many competing influences and interacting processes operating within this complex system makes a deep interpretive analysis of results quite challenging. To simplify matters we shall first consider a few basic systems before exploring more complex scenarios. In particular, two different model scenarios are considered. First, the relatively simple scenario of a uniform spatial distribution of all membrane transport proteins across the root is explored in Section 4.1. In this case all cells across the root are assumed to have the same transport proteins operating uniformly at the same level as in our single cell model (Foster and Miklavcic, 2015). That is, the membrane transport parameters, expressed per unit of membrane area where appropriate, are identical for all cells. Second, a more realistic, but significantly more complex, scenario of a non-uniform distribution of membrane transport proteins, across the root tissues and across the root zones is examined in Section 4.2. In particular, in Section 4.2, not all types of membrane transport proteins will be operating in all cells. The spatial distribution of transport proteins is based on experimental observations and is as described in Section 3.4.

The results presented here focus predominantly on steady-state Na^+^ concentrations and fluxes within the root. However, another important set of quantities are the steady-state fluxes of Na^+^ and water out of the top boundary of the root in the xylem. The latter quantities are particularly important response measures, as they represent the amount of Na^+^ and volume of water being transported from the root to the shoot via the xylem transpiration stream.

### 4.1. The effects of endodermal barriers in a root with a uniform distribution of membrane transport proteins

**Figure 3:**
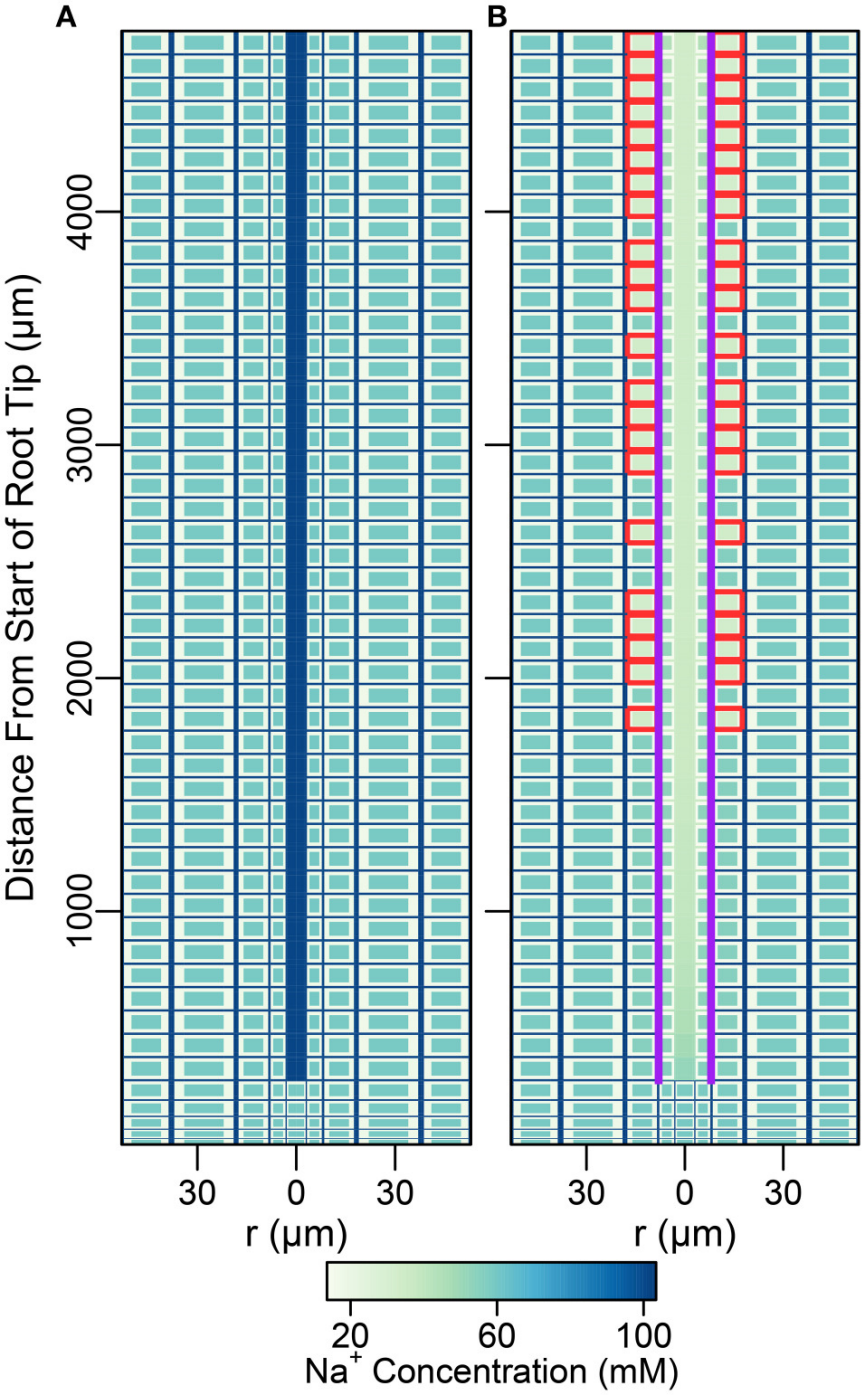
Color maps of steady-state Na^+^ concentrations assuming a uniform distribution of membrane transport proteins and two different root structures: **(A)** no endodermal barriers present; **(B)** CS and SL with passage cells present. Purple (red) lines show the location of the CS (SL). Results are shown for all three compartments (apoplast, cytoplasm, and vacuole, not drawn to scale). *P*_*b*_ = −0.5 MPa, remaining boundary conditions and transport parameters are as described in Sections 3.3 and 3.4, as well as Tables S1, S2. Compare the results of **(B)** with results shown in Figure S2B for the CS located at the cortical-endodermal interface.

**Figure 4:**
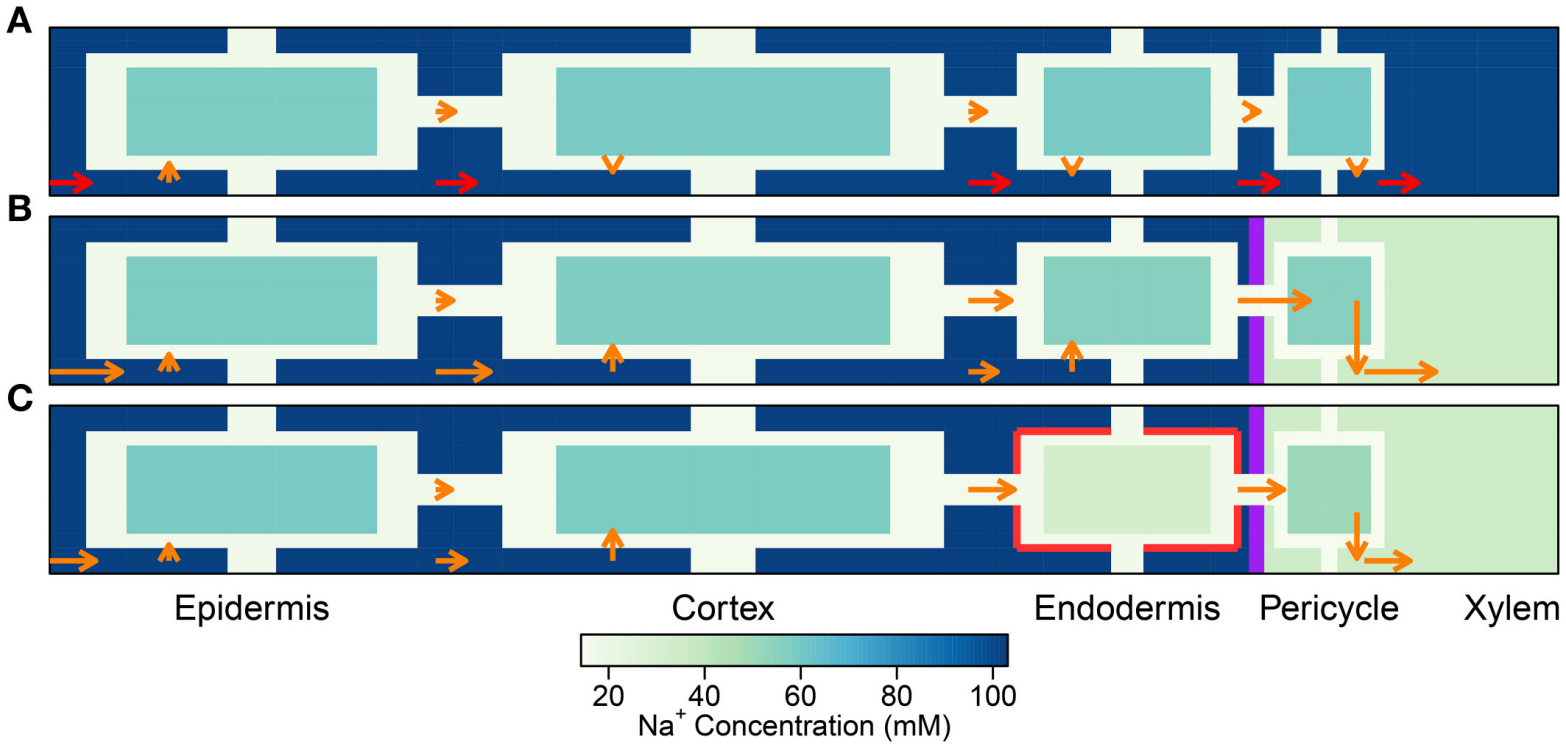
Plots of steady-state Na^+^ fluxes (arrows) and Na^+^ concentrations (color maps) at a point halfway along the differentiation zone (*j* = 28), for a uniform distribution of transporters and three different root structures: **(A)** no endodermal barriers present, **(B)** CS present, and **(C)** CS and SL present. Purple (red) lines show the location of the CS (SL). Arrows indicate relative flux magnitudes via the apoplast, symplast, and across the cell plasma membranes (for clarity, axial fluxes are not displayed). Orange arrows are drawn to the same scale across all three subfigures. Red arrows represent ***100 times larger*** fluxes relative to the orange arrows. Na^+^ concentrations are shown for all three compartments (apoplast, cytoplasm, and vacuole), although the latter are not drawn to scale (different scale to Figure 3). The simulation conditions are as described in Figure 3.

**Figure 5:**
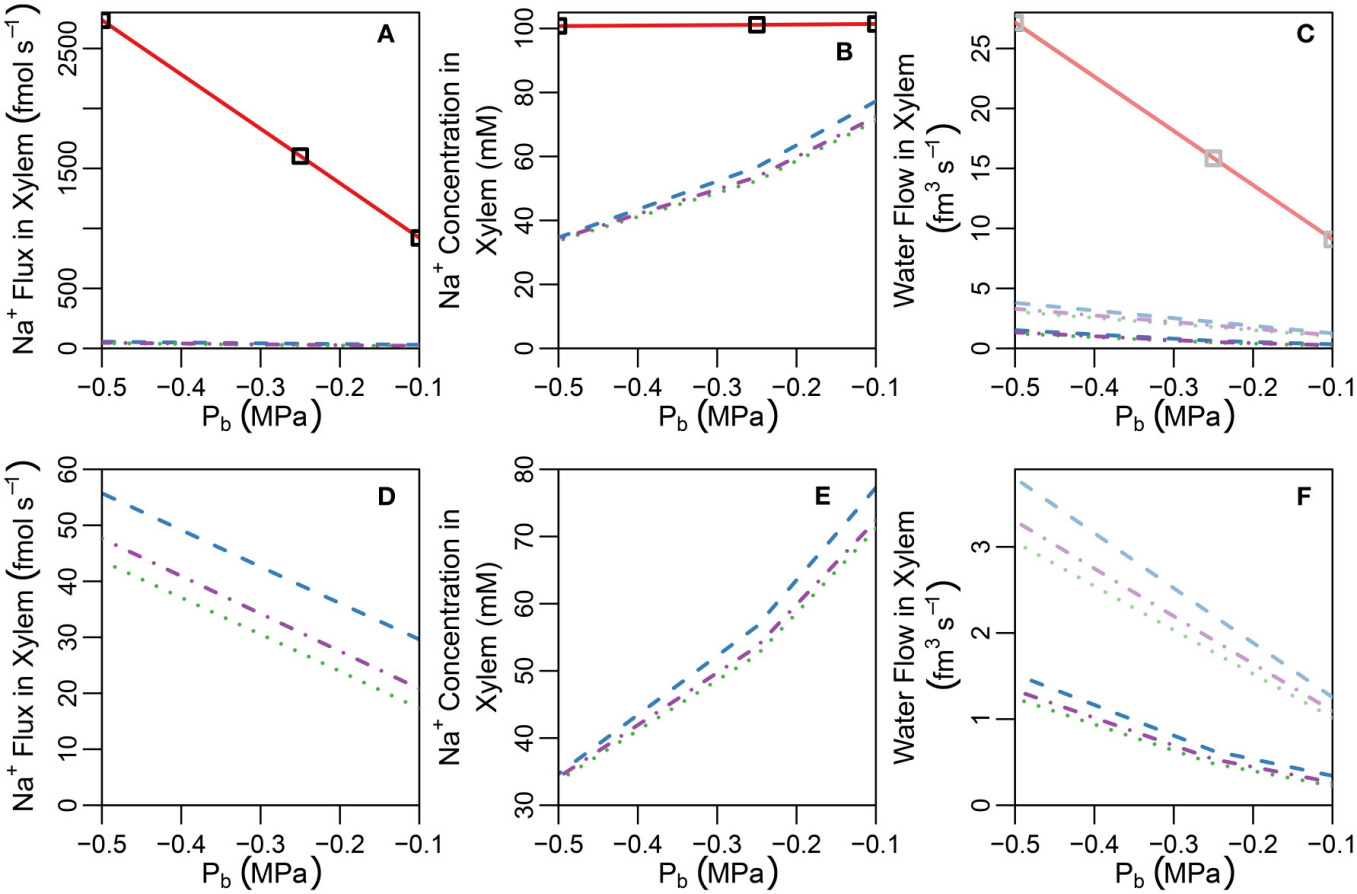
Plots of steady-state **(A)** Na^+^ concentrations, **(B)** Na^+^ fluxes, and **(C)** water flow rates, in the transpiration stream at the top of the root under pre-salt (light lines) and salt stress (dark lines) conditions, as a function of *P*_*b*_ for a uniform distribution of transporters and five different root structures (see also Figure S3). Panels **(D–F)** show the same information as **(A–C)**, respectively, excluding the no endodermal barrier scenario to highlight the effects of the SL. Line types refer to root structure: solid red lines ≡ no endodermal barriers (as shown in Figure 3A); dashed blue lines ≡ CS only; dotted green lines ≡ CS and a continuous, uninterrupted SL; and dot-dashed purple lines ≡ CS and SL with passage cells (as shown in Figure 3B). Squares represent the SL-only case (corresponding to the root structure shown in Figure S10B). The simulation conditions are as described in Sections 3.3 and 3.4, as well as Tables S1, S2.

In this section, the least complicated case of all cells having identical membrane transport properties, irrespective of tissue type and developmental zone, is used to explore the basic roles of the endodermal barriers. Figure 3 shows the steady-state Na^+^ concentrations for two different root structures under a high transpiration condition of *P*_*b*_ = −0.5 MPa (Figure S4 in the Supplementary Material shows the corresponding results for a lower transpiration condition of *P*_*b*_ = −0.1 MPa). In Figure 3A, the root is assumed to be unprotected by endodermal barriers, while Figure 3B represents the more realistic scenario in which both a completely impermeable CS and SL are present in the differentiation zone, and the SL appear in a gradual, patchy manner giving rise to passage cells, where nonetheless the CS is still present (Peterson and Enstone, 1996). The CS blocks all *apoplastic* transport of water and ions at the endodermal-pericycle interface, while the SL prevent the transport of water and ions across the *plasma membranes* of affected endodermal cells, affecting transport between the symplastic and apoplastic pathways. Intermediate between the two cases shown in Figure 3, we have also considered the cases of: a root with a completely impermeable CS only; a root with completely impermeable SL only; and a root with a completely impermeable CS and an uninterrupted, completely impermeable SL barrier present. The corresponding concentration maps for these intermediate cases do not provide any additional information to that shown by the profile in Figure 3B and therefore are not reproduced here. The steady-state symplastic, apoplastic and transmembrane Na^+^ fluxes across the different root tissues are shown in Figure 4 for a root with no endodermal barriers, the CS only, and both the CS and SL present. Although the results shown in Figures 3, 4 are for high transpiration conditions, the discussion to follow is also relevant to lower transpiration conditions. Any points of distinction arising from differences in the level of transpiration are shown in Figure 5 and are discussed as appropriate.

As found previously (Foster and Miklavcic, 2016), the influence of the endodermal barriers does not extend into the undifferentiated zone (see Figure 3). The analysis below therefore focuses on the differentiation zone only.

Despite the unrealistic scenario of a uniform distribution of identical membrane transport properties across the root, the partitioning of ions between the different cell compartments at steady state appears reasonable irrespective of the presence of endodermal barriers. For example, cytoplasmic Na^+^ concentrations are low while vacuolar Na^+^ concentrations are high (see Figures 3A,B), which is as expected from experimental observations. In addition, the transient behavior in the five different root structures appears reasonable, as the root responds in a manner that is qualitatively consistent with several key expected transient responses to salt stress (Shabala and Cuin, 2008). In particular, upon exposure to salt stress there is an immediate influx of Na^+^ into the root, leading to a very rapid depolarization of the plasma membrane (see Figure S5 in the Supplementary Material), which results in the efflux of K^+^ out of the cytoplasms into the apoplast. In addition, there is also a reduction in the concentration of bound K^+^ ions to fixed anionic groups in the porous apoplast, which may be an overestimated effect (as may also be the original extent of binding) due to the exclusion of Ca^2+^ from the model. These events in combination induce an initial rapid efflux of K^+^ out of the root into the external medium, resulting in reduced apoplastic, cytoplasmic, and vacuolar K^+^ concentrations.

The preceding discussion referred to results that were common to all five root structures. The remainder of this section focuses on their differences.

#### 4.1.1. The reference case: unrestricted transport in a root with no endodermal barriers

With no barrier to interrupt the apoplastic pathway (Figure 3A), the steady-state concentrations of the different mobile ions within the entire, continuous apoplast (including the xylem) are approximately equal to their respective concentrations in the external medium (see Figures 3A, 5B). Any small deviations are due to binding of cations to the negative charges in the apoplast as well as to the effect of the electric potential contribution from these negative charges. For a continuous, uninterrupted apoplast and with a uniform distribution of transport proteins, the steady-state cytoplasmic and vacuolar ion concentrations are also approximately constant across the root (see Figure 3A).

A key feature of note, evident in the absence of any endodermal barriers, is the very large radial apoplastic fluxes of Na^+^ toward the center of the root (see Figure 4A), allowing Na^+^ uptake to bypass the selective (symplastic) transport pathway. These large apoplastic Na^+^ fluxes lead to extremely high fluxes of Na^+^ out of the top of the root to the shoot (see Figure 5A). Such an uncontrolled uptake of Na^+^ would be tantamount to premature plant death. It is not surprising that the magnitude of Na^+^ flux (and water flow) in the xylem is strongly dependent on the level of transpiration in the absence of any endodermal barriers (Figures 5A,C), since the hydraulic pressure is a key driving force for water flow and hence solute convection in the apoplast.

The results in Figures 3A, 4A, 5A,B highlight the important fact that active membrane transport proteins cannot produce a comparable effect to, let alone substitute for, a completely impermeable Casparian strip.

#### 4.1.2. Restricted apoplastic transport in a root with a Casparian strip

The introduction of the CS leads to significant reductions in the apoplastic ion concentrations within the boundary established by the barrier (see the lower part of Figure 3B). In particular, the transient and steady-state ion concentrations in the xylem (see Figure 5B) and pericycle apoplast are significantly less than the soil concentrations, producing large step gradients in apoplastic concentrations across the barrier, which are larger for greater transpiration rates (Figure 5B). This is consistent with our previous findings based on our model assuming composite apoplastic and symplastic pathways (Foster and Miklavcic, 2016) and is a consequence of the lower axial flux of Na^+^ in the xylem at lower levels of transpiration (see Figure 5D) which leads to less Na^+^ being swept to the xylem. There is some leakage of Na^+^ (and other ions) into the xylem from the undifferentiated zone which is unprotected by the CS. However, this leakage extends a distance of only a few cells up into the xylem. The reduced levels of apoplastic Na^+^ in the tissue regions inside the CS lead on to lower cytoplasmic and vacuolar Na^+^ concentrations in the pericycle. With the interconnection of cytoplasms of adjacent cells by plasmodesmata, this also leads to slightly reduced Na^+^ concentrations in the cytoplasms and vacuoles in tissue regions *external* to the CS (particularly the endodermis). These effects are more evident in the vacuoles than in the cytoplasm compartments.

With the introduction of the completely impermeable CS, access to the xylem can only be achieved via the selective symplastic pathway (see Figure 4B). The fluxes of Na^+^ across the cell plasma membranes are substantially lower than the non-selective, unrestricted apoplastic Na^+^ fluxes found in the absence of endodermal barriers (compare Figures 4A,B). The Na^+^ fluxes and concentrations in the xylem are therefore much lower (see Figures 5A,B), representing significantly reduced transport of Na^+^ from the root to the shoot (a reduction by approximately two orders of magnitude compared with the system with no CS, see Figure 5A). While the CS has the potentially desirable effect of reducing Na^+^ uptake into the stem, its effect is non-selective and hence the uptake of K^+^ is similarly reduced (also by approximately two orders of magnitude, as shown in Figure S6 in the Supplementary Material).

For a given hydraulic pressure difference between the top of the root (the *P*_*b*_ boundary condition) and the pressure in the external medium, the CS also significantly reduces the volume flow of water in the transpiration stream (see Figure 5C). In other words, in order to maintain the same level of water uptake and thus the same water flow up into the shoot, as that observed in the absence of an apoplastic barrier, the plant would need to increase the driving force for water out of the top of the root (for example, by lowering the hydraulic pressure *P*_*b*_). This is already evident under stress free conditions (see dashed light lines in Figure 5C), but is even more pronounced following the introduction of salt (dashed dark lines in Figure 5C) due to osmotic effects. The blockage of the apoplastic pathway by the CS ensures that water must cross at least two plasma membranes to reach the xylem (see Figure S7B in the Supplementary Material). This water flow across the plasma membranes is lower than the apoplastic water flows which occur in the absence of the CS (compare Figures S7A,B in the Supplementary Material) and is driven by osmotic pressure gradients in addition to hydraulic pressure gradients. Introducing salt into the external medium increases the osmotic pressure outside the root, reducing the driving force for water uptake via the symplast (see Figure S8 in the Supplementary Material). In contrast, this osmotic effect is not significant in a root lacking a CS (see solid lines in Figure 5C, in which the pre-salt and salt-stressed results are indistinguishable in the absence of the CS), as water flow into the xylem transpiration stream under transpiring conditions, in such a root, occurs almost exclusively via the unrestricted apoplastic pathway (see Figure S7A in the Supplementary Material) and hence is driven by the larger hydraulic pressure gradients rather than osmotic pressure gradients.

These results highlight the compromise inherent in the function of the CS. While it clearly plays a key role in minimizing the non-selective uptake of toxic ions such as Na^+^, its presence also inhibits the uptake of beneficial ions such as K^+^, limits the flow of water under stress free conditions, and can have further counterproductive effects on water flow under salt stress conditions.

#### 4.1.3. Restricted transport in a root with a Casparian strip and suberin lamellae

In contrast to the dramatic difference observed with the introduction of the CS, the introduction of SL has only a minor (additional) effect on ion and water fluxes (see Figures 5D,F), and ion concentrations (see Figures 3B, 5E). The SL affect ion and water transport by reducing the plasma membrane area available for symplastic uptake. Hence, introducing the SL leads to slightly lower Na^+^ concentrations in the xylem (see Figure 5E), as well as slightly lower Na^+^ fluxes and water flow out of the top of the xylem (see Figures 5D,F). In addition, the vacuolar Na^+^ concentrations of endodermal cells affected by the SL are significantly reduced (see Figure 3B).

Only minor reductions in ion fluxes and water flow occur due to compensating effects taking place elsewhere in the symplast network. An increase in the uptake of ions and water across the plasma membranes of cells in the outer tissues (especially across the cortical cell plasma membranes) leads to increased, inwardly directed, symplastic fluxes into the endodermal cells, which help to partially offset the eliminated uptake across the plasma membranes of endodermal cells affected by SL (see Figure 4C and Figure S7C in the Supplementary Material). Hence, the local disruption to membrane transport induced by the SL at the endodermis is bypassed by an increased uptake into the symplast in the outer tissue regions. The ions and water then proceed via plasmodesmata connections through the symplast toward the xylem.

The introduction of passage cells (shown in Figure 3B) increases the endodermal plasma membrane surface area available for uptake, leading to slightly increased ion and water uptake (see dot-dashed lines in Figures 5D,F). This is reminiscent of our previous simulation results (Foster and Miklavcic, 2016), again suggesting that passage cells serve to enhance the uptake of water and ions (Peterson and Enstone, 1996).

#### 4.1.4. Relatively unrestricted transport in a root with only suberin lamellae

Interestingly, any impact of the SL on water and ion transport out of the top of the xylem is only evident if the CS is also simultaneously present. In the absence of the CS, the large apoplastic ion fluxes and water flows completely swamp the minor influence of the SL on uptake across the endodermal cell plasma membranes. Indeed, when the SL is the only endodermal barrier present, the Na^+^ fluxes and concentrations, as well as water flow rates, in the xylem are indistinguishable from the case with no endodermal barriers (see squares in Figures 5A–C). For such a root structure, the main influence of the SL on ion concentrations is a reduction in vacuolar Na^+^ concentrations in the affected endodermal cells (see Figure S10 in the Supplementary Material).

#### 4.1.5. The effect on transport of a partially permeable Casparian strip

**Figure 6:**
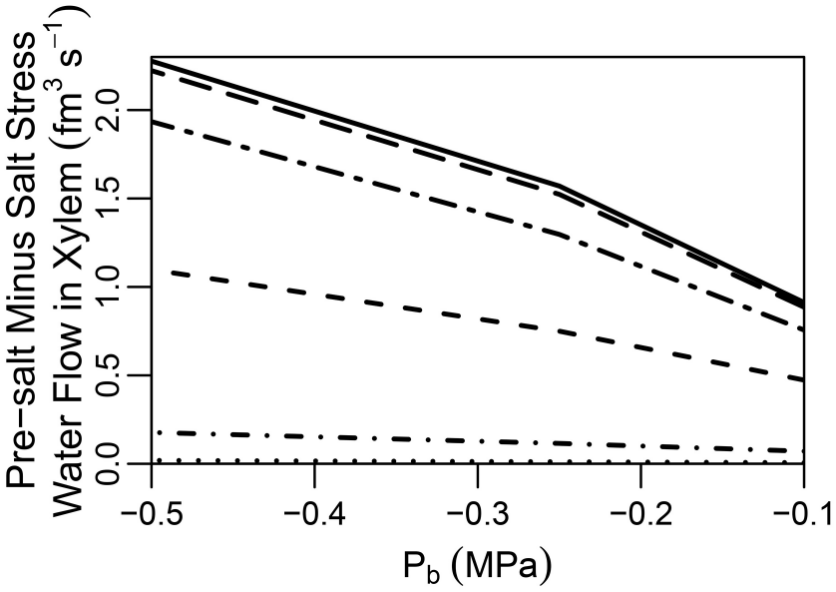
Plot of steady-state pre-salt minus salt stress water flow rates in the transpiration stream at the top of the root, as a function of *P*_*b*_ for CSs with a range of permeabilities. Line types indicate CS effectiveness: no CS present (dotted lines); $k_{\alpha \;=\;4,j_{CS}}^{a,rad,n}$ and $L_{p:\alpha \;=\;4,j_{CS}}^{a,rad}$ reduced by one order of magnitude (short dot-dashed lines); $k_{\alpha \;=\;4,j_{CS}}^{a,rad,n}$ and $L_{p:\alpha \;=\;4,j_{CS}}^{a,rad}$ reduced by two orders of magnitude (short dashed lines); $k_{\alpha \;=\;4,j_{CS}}^{a,rad,n}$ and $L_{p:\alpha \;=\;4,j_{CS}}^{a,rad}$ reduced by three orders of magnitude (long dot-dashed lines); $k_{\alpha \;=\;4,j_{CS}}^{a,rad,n}$ and $L_{p:\alpha \;=\;4,j_{CS}}^{a,rad}$ reduced by four orders of magnitude (long dashed lines); a completely impermeable CS (solid lines). The SL were excluded from all simulations. The remaining transport parameters and boundary conditions are as described in Sections 3.3 and 3.4, as well as Tables S1, S2.

It has been suggested that the CS does not completely block apoplastic transport (Ranathunge et al., 2005). Indeed, some plant species, such as rice, are thought to have a “leaky” CS which allows for non-negligible apoplastic bypass of Na^+^ to occur (Yeo et al., 1987; Yadav et al., 1996; Garcia et al., 1997). In addition, maize has been found to develop a thicker CS in response to exposure to salt stress, indicating an effectiveness when exposed to salt (Karahara et al., 2004). With these facts as motivation we have looked into the effect of a partially permeable CS. In particular, we explore the effects of reducing the transport parameters $k_{\alpha =4,j_{CS}}^{a,rad,n}$ and $L_{p:\alpha =4,j_{CS}}^{a,rad}$ at the location of the CS by one to four orders of magnitude. It should invite no surprise that a partially permeable CS produces results that are (proportionally) intermediate between the cases of a root with an impermeable CS and a root lacking a CS (see Figure S9 in the Supplementary Material). A feature of particular interest, however, is the difference between the pre-salt and the salt-stressed water flows, which becomes more evident as the CS becomes more effective (see Figure 6). As the CS is strengthened, the amount of apoplastic water flow decreases, and more water must flow through the symplast to reach the stele. As a result, the osmotic effect of high external salt then becomes more important.

**Figure 7:**
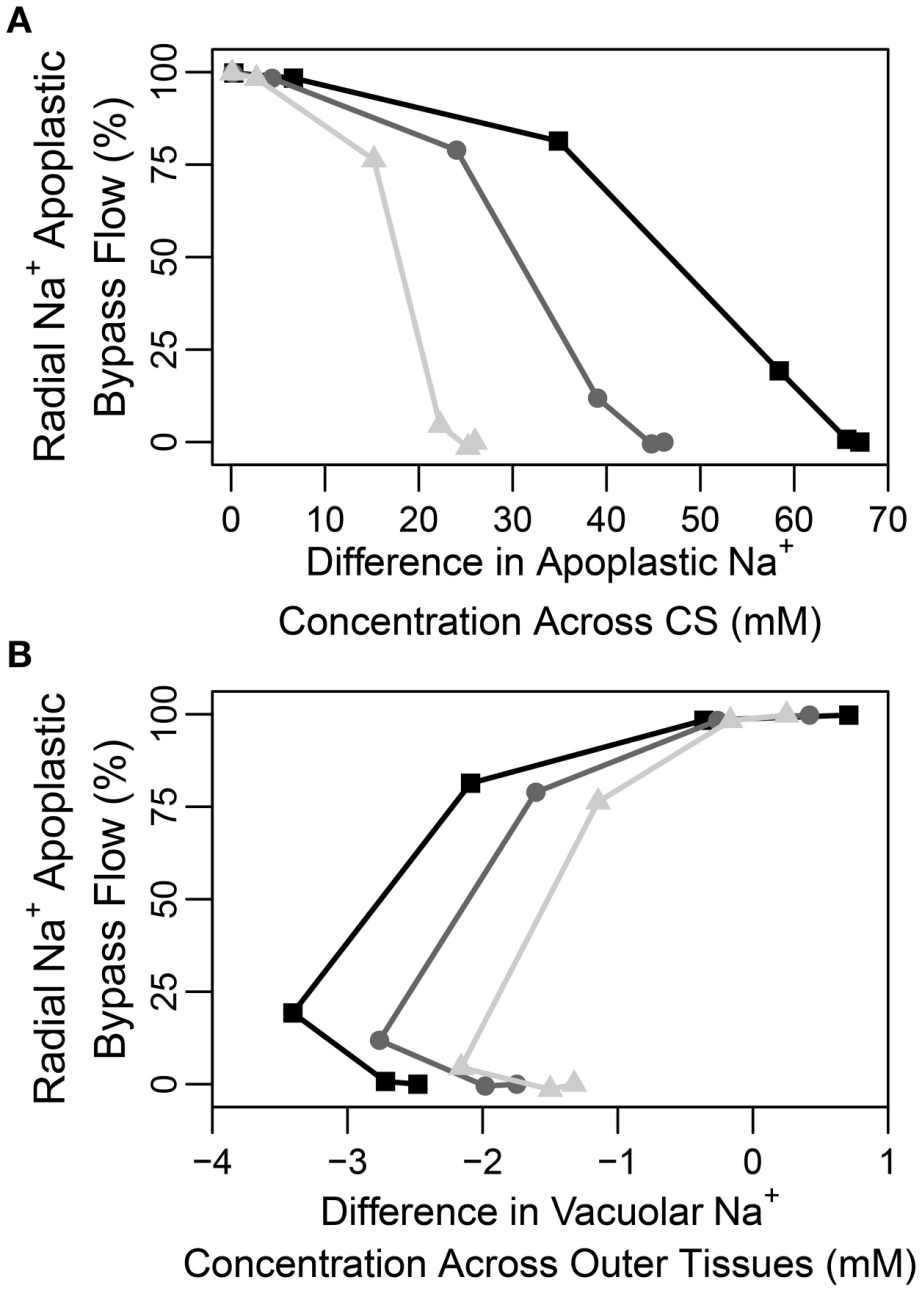
Plots of steady-state percentage of Na^+^ flux across the apoplastic endodermis-pericycle interface (apoplastic bypass flow) vs. **(A)** the difference in apoplastic Na^+^ concentration across the CS (the apoplastic Na^+^ concentration in the endodermis minus the apoplastic Na^+^ concentration in the pericycle), and **(B)** the difference in vacuolar Na^+^ concentration across the outer tissues (the vacuolar Na^+^ concentration in the endodermis minus the vacuolar Na^+^ concentration in the epidermis), based on the range of partially permeable CSs shown in Figure 6. The results are shown for three different levels of transpiration: *P*_*b*_ = −0.1 MPa (light gray triangles), *P*_*b*_ = −0.25 MPa (dark gray circles), and *P*_*b*_ = −0.5 MPa (black squares). The percentage of apoplastic bypass flow of Na^+^ was determined using: 100×(radial apoplastic flux of Na^+^ across the endodermis − pericycle interface)/total radial Na^+^ flux across the endodermis-pericycle interface. The total flux is the sum of the symplastic and apoplastic fluxes. All concentrations were taken at a point halfway along the DZ (*j* = 28). The simulation conditions are as described in Figure 6.

A question that naturally arises here is how best to quantify the CS's effectiveness in a given experimental situation. Figure 7A illustrates that the difference in apoplastic Na^+^ concentration across the barrier may be one representative measure of effectiveness as there is a correlation between this concentration gradient and the apoplastic bypass of Na^+^. This correlation is evident under all transpiration conditions but is clearer under higher levels of transpiration. In contrast, there is no clear direct relationship between the change in vacuolar Na^+^ concentration across the CS and its effectiveness (see Figure S11A in the Supplementary Material).

In none of the simulations (irrespective of the effectiveness of the CS) did we find any large radial concentration gradients across the apoplast external to the CS. We conclude from this that there is no clear relationship between an apoplastic concentration gradient across the outer tissues and CS effectiveness (see Figure S11B in the Supplementary Material). This is reminiscent of our previous findings (Foster and Miklavcic, 2016) and likely reflects the contribution of diffusion to transport in the apoplast which will act to eliminate any concentration gradients. Similarly, there is no clear direct relationship between vacuolar concentration gradients across the tissues external to the CS and the effectiveness of the barrier (see Figure 7B).

### 4.2. Modified transport in a root with endodermal barriers and a non-uniform distribution of membrane transport proteins

It is not typical nor expected that membrane transport proteins are uniformly active across the length and breadth of a root. Indeed, a significant number of transporters are known to be active in specific tissue and developmental regions only. It thus warrants considering how the influences of the CS and SL might alter in a scenario where membrane transport proteins are no longer uniformly active, as described in Section 3.4 and Table 1.

The steady-state Na^+^ concentration results for the selected distribution of membrane transport proteins, in the presence and absence of the endodermal barriers, is shown in Figure S12 in the Supplementary Material. In agreement with the findings of the previous section (Section 4.1), the CS has a much greater effect on both the transient and steady-state ion and water behavior than do the SL (see Figure S13 in the Supplementary Material). Again, the CS plays the leading role in preventing excessive amounts of Na^+^ from reaching the xylem transpiration stream via the apoplast (see Figure S13A). Here too, the CS has the disadvantage of reducing the uptake of water and increasing the osmotic stress experienced by the root (see Figure S13B).

**Figure 8:**
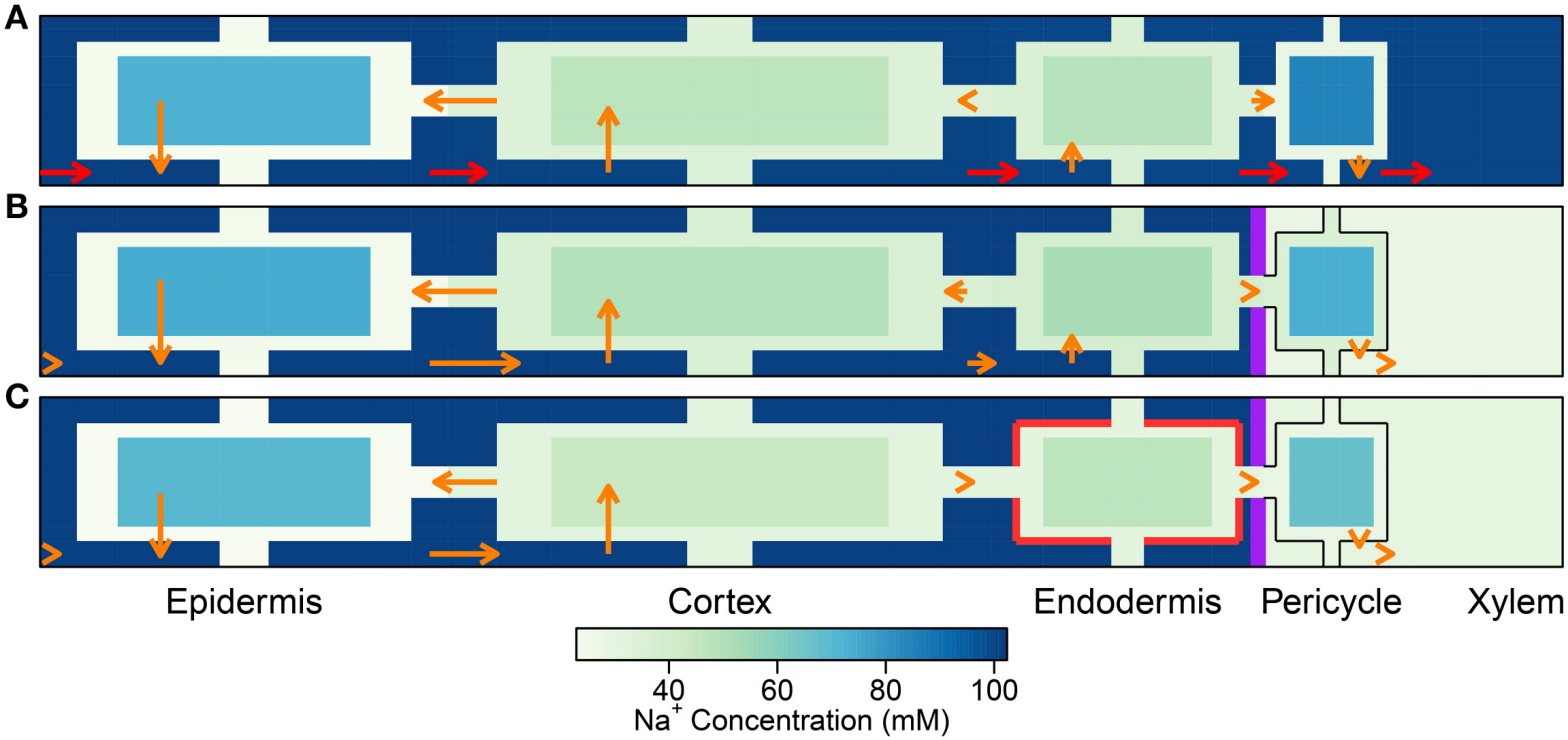
As per Figure 4 except for the non-uniform distribution of membrane transport proteins as described in Section 3.4. Simulations were conducted using *P*_*b*_ = −0.5 MPa and conditions and parameters as described in Sections 3.3 and 3.4, as well as Table 1 and Tables S1, S2.

**Figure 9:**
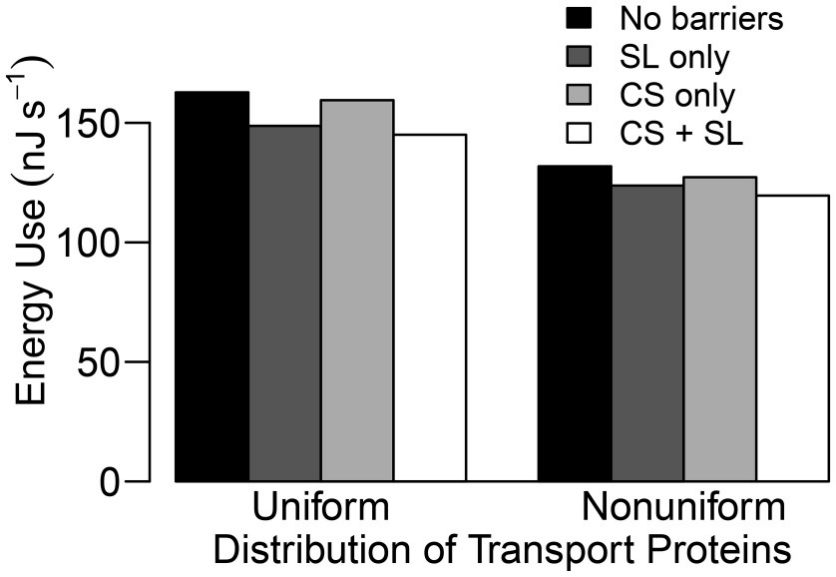
Steady state energy use histograms for a uniform and a non-uniform distribution of transporters as described in Section 3.4 for four different root structures: no endodermal barriers (black); SL only (dark gray); CS only (light gray); CS and SL present (white). Energy use is estimated by the energy released from hydrolysis of ATP (approximately 54.8 kJ per mole of ATP hydrolyzed (Schmidt and Briskin, 1993) and hence, 54.8 kJ per mole of H^+^ pumped across the plasma membranes) and hydrolysis of PP_*i*_ (≈24.9 kJ per mole of H^+^ pumped across the tonoplasts, Schmidt and Briskin, 1993). This is a measure of the energy used to pump H^+^ which cannot then be used by the plant for other processes. Qualitatively identical results are obtained if the total steady state flux of H^+^ via all pumps is used as a proxy for energy use. Simulations were conducted using *P*_*b*_ = −0.5 MPa with remaining conditions and parameters as described in Sections 3.3 and 3.4, as well as Table 1 and Tables S1, S2.

While the SL still have a minor effect on the transport of Na^+^ and water, with a more realistic distribution of transport proteins (see Figure S13 in the Supplementary Material), a potentially important role becomes evident when the Na^+^ fluxes are examined in detail. Since the SL prevent passive leakage of ions into the symplast, across the plasma membranes of affected endodermal cells, they reduce the futile cycling of Na^+^ between cells of the outer tissues (see Figure 8). Here, use of the term “futile cycling” (of Na^+^) refers to the passive influx of Na^+^ into a cell in one tissue region which offsets the active efflux occurring in a cell in a different tissue region. For example, in the absence of SL, there is passive leakage of Na^+^ across the plasma membranes of both endodermal and cortical cells into the symplast (see Figures 8A,B). These Na^+^ ions are carried via symplastic fluxes from endodermal cells toward epidermal cells as the action of the epidermal plasma membrane Na^+^/H^+^ antiporters draw Na^+^ out of the symplast, actively ejecting Na^+^ back out into the apoplast. In the presence of the SL, however, this futile cycling is restricted to only the cortical cells (see Figure 8C). The greater the amount of Na^+^ that leaks passively into the symplast, the greater is the amount of Na^+^ required to be actively transported out of the symplast and hence the greater is the energy required in order to maintain a low level of cytoplasmic Na^+^. Consequently, there is a reduction in total energy use at steady state when the SL are present (see Figure 9).

It is important to point out that the level of influence of the SL depends on the tissue-specific location of membrane transport proteins. For example, the futile cycling event described above is not apparent in the case of a uniform distribution of transport proteins (see Figure 4). However, regardless of the location of the membrane transport proteins, the presence of SL means that affected cells do not need to expend energy in order to prevent Na^+^ from passively leaking from the high Na^+^ environment in the apoplast to the low Na^+^ environment in the symplast. This is reflected in the reduced energy used in all instances where the SL are present (see Figure 9). Indeed, energy usage is the only measure of the influence of the endodermal barriers for which the SL have a greater effect than does the CS.

## 5. Discussion

In this paper, we have used a mathematical model to differentiate and clarify the distinct roles played by the CS and SL in the salt stress response of plants. Based on current hypotheses about the specific transport pathways these barriers affect (Enstone et al., 2002; Geldner, 2013; Barberon and Geldner, 2014; Barberon et al., 2016), our simulations highlight the much more vital function of the CS in the salt stress response, compared to the SL.

The CS is essential for the control Na^+^ uptake into the shoot (regardless of the distribution of membrane transport proteins), by preventing excessive levels of Na^+^ from reaching the xylem transpiration stream directly via the apoplast. Our findings emphasize the potential magnitude of apoplastic bypass flows (see Figures 4A, 5A), supporting the idea that apoplastic bypass of Na^+^ could be a significant pathway by which Na^+^ could reach the xylem and hence be a key contributor to salt sensitivity in some plant species (Yeo et al., 1987; Yadav et al., 1996; Garcia et al., 1997; Kronzucker and Britto, 2011). Our study highlights the need to quantify the function of the CS when investigating the salt tolerance mechanisms utilized by all plant species. In particular, there is a need to specifically quantify the role of the CS in the salt stress response of plant species, such as *Arabidopsis*, for which apoplastic bypass has not yet been fully investigated (Kronzucker and Britto, 2011). In addition, the effect of growth conditions on the development of the CS should be considered in salt stress experiments as changes to this barrier could result in significant changes in the plant's salt stress response. For example, plants grown in hydroponic conditions may develop a less effective CS compared to those grown in soil (Kronzucker and Britto, 2011). Our study has also drawn attention to the potential to improve salinity tolerance (in terms of enhanced Na^+^ exclusion from the shoot) by modification of genes that control or influence the formation of the CS. Such genetic manipulation may be possible in the future as indicated by the recent identification of *sgn3*, an *Arabidopsis* mutant with a non-functional CS (Pfister et al., 2014). Given our results and the suspected importance of the CS (Enstone et al., 2002), the effect of the non-functional CS on ion homeostasis was surprisingly small (Pfister et al., 2014). On the other hand, these experiments were not conducted under salt-stressed conditions.

Our findings are in conflict with those of Lauchli et al. (2008), who concluded that the CS was not an important control point for Na^+^ and Cl^−^ uptake in wheat roots. However, their conclusion was based on the observation that there was no build up of Na^+^ and Cl^−^ ions in the vacuoles of cortical cells, which was assumed to reflect a lack of build up of these ions in the apoplast outside the CS. The results presented here show that while an effective CS does create large concentration gradients (for all ions) between *apoplastic* regions of the inner and outer tissues (see Figure 7A), only a minor concentration difference appears between cell vacuoles on either side of the barrier (see Figure S11A in the Supplementary Material). In addition, although the CS clearly acts as a control point for ion uptake, it does not create a large gradient in apoplastic ion concentrations from the cortex to the epidermis and this gradient does not have a clear correlation with the effectiveness of the CS (see Figure S11B in the Supplementary Material). Hence, no large difference is observed in the vacuolar concentrations and there is no clear relationship between these vacuolar concentration differences and the effectiveness of the barrier (see Figure 7B). In addition, ion concentrations in the vacuoles of different tissues are also affected by the action of membrane transport proteins which may be localized to specific tissues, further complicating the use of vacuolar concentrations as a measure of the influence of the CS. In general, it can be concluded that a large difference in apoplastic concentrations between inner and outer regions, and not vacuolar ion concentration gradient across the outer tissues, could be used as a measure of CS functionality (compare Figures 7A,B).

An important effect of the CS that is highlighted by our simulations is that the exclusion of Na^+^ achieved by the CS occurs at the cost of reduced water flow under salt stress conditions (see Figures 5, 6 and Figure S13B in the Supplementary Material) as it brings the osmotic effect of salt stress into play. Hence, the inherent compromise between control of ion uptake and reduced water uptake by the CS leads to a compromise between ionic and osmotic salt tolerance. This suggests that a partially permeable CS may have the advantage of increased water uptake under salt-stressed conditions at the cost of an increase in non-selective transport of Na^+^ (see Figure 6 and Figure S9A in the Supplementary Material), indicating that plant species with a leaky CS may have opted for improved osmotic stress tolerance at the cost of reduced ionic stress tolerance. Efforts to engineer more salt tolerant plants by altering the formation of the CS would need to take into account the fact that altering this barrier is likely to impact on water uptake.

In contrast to the essential role played by the CS, the impact of the SL was found to be much more subtle (see Figure 5). These findings support the suggestion by Enstone et al. (2002) that the SL, unlike the CS, have a non-essential function. Our simulations have shown that the relatively minor impact of the SL is a consequence of the continuous and connected nature of the symplast. In particular, our results support the proposal (Enstone et al., 2002; Barberon et al., 2016) that the impact of the SL can be at least partially offset by increased uptake across plasma membranes in the outer tissue regions (see Figure 4 and Figure S7 in the Supplementary Material). Our simulations (Figure 5D) match the experimental findings that increased suberization leads to reduced Na^+^ uptake in the shoot and leaves (Krishnamurthy et al., 2009, 2011), presumably leading to an improved salt stress response. However, our results also indicate that the magnitude of this effect is small compared to the influence of the CS (Figure 5A). Interestingly, even this small influence is only apparent if the CS is also present (see Figure 5A). The apparently minor impact of the SL raises the question of why increased suberization is a commonly observed response to salt stress (Schreiber et al., 2005; Krishnamurthy et al., 2009, 2011; Barberon et al., 2016). However, our results do suggest that the SL may play a role in preventing futile cycling of Na^+^ between cells and across the endodermal cell plasma membranes, hence reducing the energy cost of active efflux of Na^+^ out of the root (see Figures 8, 9), although this specific effect depends on where the plasma membrane transport proteins are found. For example, if a membrane transport protein involved in the salt stress response was found to operate *only* in the endodermis, the SL would likely induce a greater effect.

The results presented here highlight the importance of including the continuity of the symplast when modeling solute and water transport in roots. Indeed, the inclusion of plasmodesmata connections in the model has allowed the influence of the SL to be explored. However, plasmodesmata are typically not included in existing models (Grieneisen et al., 2007; Sakurai et al., 2015; Shimotohno et al., 2015), with a key exception being the zinc model developed by Claus et al. (2013).

For reasons of space we have not presented a direct comparison of the results of this model with those of the composite-pathway, passive transport model of Foster and Miklavcic (2016). However, in many instances the results of the two models are qualitatively comparable. For example, simulations using both models have highlighted the compromise inherent in the function of the CS in that it restricts toxic ion uptake but also limits water uptake. In addition, in the simulations of both models the concentration of ions in the xylem was higher under lower transpiration conditions. Indeed, in simulations *with* the CS present (with or without the SL), the xylem ion flux results obtained with the two models are quite comparable, quantitatively (compare the present Figure 5A with Figure 3 in Foster and Miklavcic, 2016), which gives some confidence in the relevance of the results using the passive composite model. On the other hand, these two models predict significantly different xylem ion fluxes in the *absence* of the CS, being an order of magnitude larger in the current model which distinguishes the apoplast from the symplast. Although the results will depend somewhat on the choice of parameters, the difference suggests that the composite model lends itself to favor the symplastic pathway in scenarios in which the CS is absent.

The model formulated here included a higher degree of spatial discretization, compared to our previous root models (Foster and Miklavcic, 2013, 2014, 2016), due to the explicit simulation of the apoplastic and symplastic pathways. Several existing root models (e.g., Grieneisen et al., 2007; Sakurai et al., 2015; Shimotohno et al., 2015) have used more detailed spatial discretization to represent each cell cytoplasm by multiple grid points, allowing concentration gradients across cell cytoplasms to be modeled. However, the appropriate degree of discretization depends on the intended aim (Krupinski and Jönsson, 2010). For example, a detailed spatial discretization which uses multiple grid points (or volume compartments) to represent the cytoplasm of a single cell is necessary when investigating transport scenarios which are anticipated to create concentration gradients across the cytoplasm of each cell. Such detail has been used to explore the effects of membrane transport proteins which are non-uniformly distributed across the individual cell plasma membranes (e.g., Grieneisen et al., 2007; Sakurai et al., 2015; Shimotohno et al., 2015). However, for our purposes, it is far more important to model the partitioning of ions between the cytoplasm and vacuole, because of the anticipated large concentration differences between these compartments and particularly because of the importance of ion storage in the vacuole as a salt tolerance mechanism. If any of the key membrane transport proteins involved in the salt stress response were purported to be polarly distributed, then a more detailed spatial discretization could be incorporated into the model to explore the phenomenological implications. A more refined spatial discretization would also allow the location of the CS to be modeled with greater accuracy.

In this paper, we have focused on steady state behavior as this is likely to be of greatest interest to plant researchers. Only sparing references have been made to transient behavior. However, it is worth noting that the steady state results conceal a great deal of information about the immediate reactions of plants to salt exposure. Some of these aspects will be described in a separate report. Of particular relevance to the present paper, however, are the differences in times taken for the different systems we have studied to reach steady state. For the majority of the systems we have studied, the pre-salt stage achieved steady state over the course of two simulated days. Similarly, steady state for the salt stress stage in the case of no endodermal barriers is achieved in one-to-two simulated days. On the other hand, depending somewhat on the strengths of active transporters, the CS and SL substantially extend by an order of magnitude or more the time it takes to attain steady state in the tissues within the barriers. Presumably, this arises from the gradual but continual leakage of the salt from the apoplastic region of high concentration external to the barriers, into the symplastic region of low concentration. The results that appear in this paper for the latter case are thus chosen once qualitatively time-independent behavior across all variables is found. The results are thus more appropriately described as quasi-steady state. Notwithstanding this qualifying description, the relative behavior and function of the barriers are correctly represented here. A study of the effects of different transporters on the approach to steady state will be covered in a separate report.

Clearly, the root model presented here involves a vast number of transport proteins and a diverse range of locations for activity of these proteins. The inherent scale of the model opens up the possibility to explore an enormous, but still finite, number of interactions between different transport mechanisms. However, this also introduces a large number of model parameters to consider, and vary. The membrane transport parameters used here were chosen based on experimentally measured behavior of rice protoplasts using the parameter fitting process described in Foster and Miklavcic (2015). However, there is some uncertainty about how well the function of protoplasts reflect the function of cells in living plants (Anil et al., 2007). In addition, by its nature the parameter fitting procedure could not take into account the properties of cells in different tissue types. These shortcomings have been partially offset through the use of literature data on the tissue-specific expression of transport genes and would be further addressed by a systematic parameter sensitivity analysis. However, greater confidence in the model results (and greater certainty in its ability to represent a specific plant species) could be achieved by using independent, whole-of-root experimental data to determine any unknown model parameters. Various experimental techniques are available which could be used to determine these parameters including: energy dispersive X-ray microanalysis and cryo-scanning electron microscopy imaging (e.g., Lauchli et al., 2008) which could be used to quantitatively determine the concentration of Na^+^, Cl^−^, and K^+^ in the vacuoles of cells in a cross-section of the root; microelectrodes to measure ion fluxes across the root surface, although there are difficulties in measuring Na^+^ fluxes specifically (Shabala et al., 2005); fluorescent dyes to determine the subcellular distribution of ions (e.g., Park et al., 2009; Wu et al., 2015), although in planta concentrations have not yet been quantitatively determined using this technique; and analysis of xylem exudate to determine ion content. Moreover, several different experimental techniques could be combined. In addition, the experimental results from wild type and genetically modified plants could be used to fit the model parameters in order to provide an in depth analysis of the effects of the specific genetic modification under consideration.

## 6. Conclusion and future direction

A functional CS is essential to restrict the amount of Na^+^ being transported from the root to the shoot. In contrast, functional SL appear to be useful but non-essential in the salt stress response of plants in that they provide a relatively minor reduction in Na^+^ uptake. Hence, attempts to engineer or breed plants with improved salinity tolerance by influencing the formation of the endodermal barriers are likely to achieve more success by focusing on the CS rather than the SL.

In this paper, we have presented only a small sample of the vast spectrum of scenarios which can be explored with this comprehensive model. A wealth of additional information is yet to be uncovered about the response of roots to salt stress. Further simulations using this root model have yet to be conducted to explore the effects of the full gamut of membrane transport proteins that play a role in the salt stress response. Indeed, comparisons between our simulations and experimental observations could be used to verify suspected transport mechanisms or even identify unknown mechanisms of transport. As a particular example, in the next stage of studies we will apply this model to explore the function(s) of plasma membrane Na^+^/H^+^ antiporters in roots. The results of these efforts will be reported in a separate publication.

## Author contributions

The authors were equal contributors to the design of simulations, analysis of results, and drafting of the paper. KF performed the simulations.

### Conflict of interest statement

The authors declare that the research was conducted in the absence of any commercial or financial relationships that could be construed as a potential conflict of interest.
