# Supplementary material for: A Comprehensive Biophysical Model of Ion and Water Transport in Plant Roots. I. Clarifying the Roles of Endodermal Barriers in the Salt Stress Response
Source: Front Plant Sci. 2017 Jul 28;8:1326. doi: 10.3389/fpls.2017.01326 (PMC5532442; doi:10.3389/fpls.2017.01326)
Supplement: Supplementary file 1 [file DataSheet2.PDF]

Supplementary Material: A comprehensive biophysical model of ion and water transport in plant roots. I. Clarifying the roles of endodermal barriers in the salt stress response

Kylie J. Foster and Stanley J. Miklavcic*
*Correspondence:
Stanley J. Miklavcic:
Stan.Miklavcic@unisa.edu.au

S.1. WATER AND ION TRANSPORT

S.1.1 Governing equations of water transport

As in our single cell model (Foster and Miklavcic, 2015), it is assumed that the flow rate of water across the tonoplast is driven by osmotic pressure differences across the membrane. Hydraulic pressure gradients across this membrane are not considered as the tonoplast membrane (unlike the plasma membrane) is unconstrained by cell walls and hence is incapable of supporting hydraulic pressure gradients (Evans, 2009). Hence, the flow rate of water into the vacuole of each cell in each (α, j) element, $Q^t_{\alpha,j}$, is given by,

$$Q^t_{\alpha,j} = A^t_{\alpha,j} L^t_p \left(-\Pi^c_{\alpha,j} + \Pi^v_{\alpha,j}\right), \tag{S1}$$

where $A^t_{\alpha,j}$ is the surface area of the tonoplast in each individual cell in element (α, j), L^t_p is the water permeability of the tonoplast and Π^c (Π^v) refers to the osmotic pressure in the cytoplasm (vacuole). The van't Hoff relation for osmotic pressure ($\Delta\Pi^n = R_g T \Delta C^n$), which is valid for dilute solutions (Katchalsky and Curran, 1965), is used to give,

$$Q^t_{\alpha,j} = A^t_{\alpha,j} L^t_p R_g T \left(-\sum_{n=1}^{N^c_{osm}} C^{c,n}_{\alpha,j} + \sum_{n=1}^{N^v_{osm}} C^{v,n}_{\alpha,j}\right), \tag{S2}$$

where $C^{c,n}_{\alpha,j}$ ($C^{v,n}_{\alpha,j}$) is the concentration of ion n in the cytoplasm (vacuole) in element (α, j), R_g is the Universal gas constant (8.314 J mol^{-1} K^{-1}), T is the temperature and N_{osm} is the total number of solute species contributing to the osmotic pressure (which may vary between cellular compartments). Refer to Foster and Miklavcic (2014) for a discussion on the validity of the use of the van't Hoff relation.

The flow rate of water from the symplast into the apoplast across the plasma membrane of each individual cell in element (α, j), $Q^p_{\alpha,j}$, is driven by both hydrostatic and osmotic pressure gradients. Using non-equilibrium thermodynamics (Katchalsky and Curran, 1965),

$$Q^p_{\alpha,j} = A^p_{\alpha,j} L^p_{p:\alpha,j} \left[p^c_{\alpha,j} - p^a_{\alpha,j} - \left(\Pi^c_{\alpha,j} - \Pi^a_{\alpha,j}\right)\right], \tag{S3}$$

where $A^p_{\alpha,j}$ is the surface area of the plasma membrane of each individual cell in element (α, j), $L^p_{p:\alpha,j}$ is the water permeability of the plasma membrane, $p^c_{\alpha,j}$ is the cell turgor pressure, $p^a_{\alpha,j}$ is the hydrostatic pressure in the apoplast and Π^a is the osmotic pressure in the apoplast. Again using the van't Hoff relation for osmotic pressure,

$$Q^p_{\alpha,j} = A^p_{\alpha,j} L^p_p \left[p^c_{\alpha,j} - p^a_{\alpha,j} \right. \\ \left. -R_g T \left(\sum_{n=1}^{N^c_{osm}} C^{c,n}_{\alpha,j} - \sum_{n=1}^{N^a_{osm}} C^{a,n}_{\alpha,j} \right) \right] \tag{S4}$$

where $C^{a,n}_{\alpha,j}$ is the concentration of ion n in the apoplast in element (α, j).

Note that, Eqs. (S1) to (S4) involve the assumption that the reflection coefficients of cell membranes for all solutes considered are unity (Steudle, 1994).

The radial water flow rate into the cytoplasm of a cell in (α, j) from the cytoplasm of a cell in region $(\alpha - 1, j)$ via plasmodesmata, $Q^{s,rad}_{in,\alpha,j}$, is also driven by hydraulic and osmotic pressure gradients. The plasmodesmata can be modeled as pores in a membrane (Murphy, 1989), and hence $Q^{s,rad}_{in,\alpha,j}$ is given by,

$$Q^{s,rad}_{in,\alpha,j} = A^{s,rad}_{\alpha,j} L^s_p \left[p^c_{\alpha-1,j} - p^c_{\alpha,j} \right. \\ \left. -R_g T \sum_{n=1}^{N^c_{osm}} \sigma^{s,n} \left(C^{c,n}_{\alpha-1,j} - C^{c,n}_{\alpha,j} \right) \right], \tag{S5}$$

where $A^{s,rad}_{\alpha,j}$ is the total surface area which contains plasmodesmata connecting the symplast of elements $(\alpha - 1, j)$ and (α, j), L^s_p is the symplastic water permeability, and $\sigma^{s,n}$ is the reflection coefficient of solute $n = 1, \ldots, N^c_{osm}$. Note that in the outermost tissue region ($\alpha = 1$, the epidermis) and the innermost tissue region at the location of functional xylem ($\alpha = 5$, for j in the differentiation zone), $Q^{s,rad}_{in,\alpha,j} = 0$ since there are no connecting cells.

Similarly, the axial water flow rate into the cytoplasm of a cell in (α, j) from the cytoplasm of a cell in region $(\alpha, j - 1)$ via plasmodesmata, $Q^{s,ax}_{in,\alpha,j}$, is given by,

$$Q^{s,ax}_{in,\alpha,j} = A^{s,ax}_{\alpha,j-1} L^s_p \left[p^c_{\alpha,j-1} - p^c_{\alpha,j} - \rho_w g l^{ax}_{\alpha,j} \right. \\ \left. -R_g T \sum_{n=1}^{N^c_{osm}} \sigma^{s,n} \left(C^{c,n}_{\alpha,j-1} - C^{c,n}_{\alpha,j} \right) \right], \tag{S6}$$

where $A^{s,ax}_{\alpha,j-1}$ is the total cross-sectional area which contains plasmodesmata connecting the symplast of elements $(\alpha, j - 1)$ and (α, j), ρ_w is the density of water, g is the acceleration due to gravity and $l^{ax}_{\alpha,j}$ is the height of element (α, j). Note that for the model elements at the bottom of the root, $Q^{s,ax}_{in,\alpha,j=1} = 0$ since there are no connecting cells.

Eqs. (S1) to (S6) apply only in those (α, j) elements in which cells are present. In contrast, in the non-living xylem, cells are not present and $Q^t_{\alpha=5,j_{DZ}} = Q^p_{\alpha=5,j_{DZ}} = Q^{s,rad}_{\alpha=5,j_{DZ}} = Q^{s,ax}_{\alpha=5,j_{DZ}} = 0$ (where j_{DZ} refers to all j in the differentiation zone).

The radial apoplastic water flow rate into element (α, j), $Q^{a,rad}_{in,\alpha,j}$, is driven by hydraulic pressure gradients only, and hence is given by,

$$Q^{a,rad}_{in,\alpha,j} = A^{a,rad}_{\alpha,j} L^{a,rad}_{p:\alpha,j} \left(p^a_{\alpha-1,j} - p^a_{\alpha,j} \right), \tag{S7}$$

where $A^{a,rad}_{\alpha,j}$ is the surface area of the apoplast separating elements $(\alpha - 1, j)$ and (α, j) and $L^{a,rad}_{p:\alpha,j}$ is the water permeability of the apoplast across the $(\alpha - 1)$-to-α interface.

Similarly, the axial water flow rate into element (α, j) via the apoplast, $Q^{a,ax}_{in,\alpha,j}$, is given by,

$$Q^{a,ax}_{in,\alpha,j} = L^{a,ax}_{p:\alpha,j-1} A^{a,ax}_{\alpha,j-1} \left(p^a_{\alpha,j-1} - p^a_{\alpha,j} - \rho_w g l^{ax}_{\alpha,j} \right). \tag{S8}$$

where $A^{a,ax}_{\alpha,j-1}$ is the cross-sectional area of the apoplast separating elements $(\alpha, j-1)$ and (α, j) and $L^{a,ax}_{p:\alpha,j-1}$ is the water permeability of the apoplast across the $(j - 1)$-to-j interface.

Eqs. (S7) and (S8) involve the assumption that the reflection coefficient of all of the solutes considered in the apoplast is zero (Steudle, 1994).

The radial outflow from the apoplast of an element is equal to the radial apoplastic inflow into its neighbor element,

$$Q^{a,rad}_{out,\alpha,j} = Q^{a,rad}_{in,\alpha+1,j} \tag{S9}$$

and similarly the radial outflow from the symplast of the cytoplasms in an element is equal to the radial symplastic inflow into the cytoplasms in its neighbor element,

$$Q^{s,rad}_{out,\alpha,j} = Q^{s,rad}_{in,\alpha+1,j}. \tag{S10}$$

The axial outflow from the apoplast of an element is equal to the axial apoplastic inflow into its neighbor,

$$Q^{a,ax}_{out,\alpha,j} = Q^{a,ax}_{in,\alpha,j+1}, \tag{S11}$$

and similarly the axial outflow from the symplast of the cytoplasms in an element is equal to the axial symplastic inflow into the cytoplasms in its neighbor element,

$$Q^{s,ax}_{out,\alpha,j} = Q^{s,ax}_{in,\alpha,j+1}. \tag{S12}$$

S.1.2 Governing equations of apoplastic and symplastic ion transport

Similar to the ion flux equations applied in our earlier root models (Foster and Miklavcic, 2013, 2014, 2016), the flux of ions through the apoplast can be expressed by an extended Nernst-Planck equation (van der Horst et al., 1995), with the radial apoplastic flux of ion n, $S^{a,rad,n}_{in,\alpha,j}$, given by,

$$S^{a,rad,n}_{in,\alpha,j} = -k^{a,rad,n}_{\alpha,j} A^{a,rad}_{\alpha,j} \left[C^{a,n}_{\alpha,j} - C^{a,n}_{\alpha-1,j} + \frac{Z^n C^{a,n}_{\alpha-1,j} F}{R_g T} \left(\psi^a_{\alpha,j} - \psi^a_{\alpha-1,j} \right) \right] + C^{a,n}_{\alpha-1,j} Q^{a,rad}_{in,\alpha,j}, \tag{S13}$$

and the axial apoplastic flux of ion n, $S^{a,ax,n}_{in,\alpha,j}$, given by,

$$\begin{aligned} S^{a,ax,n}_{in,\alpha,j} &= -k^{a,ax,n}_{\alpha,j-1} A^{a,ax}_{\alpha,j-1} \Big[C^{a,n}_{\alpha,j} - C^{a,n}_{\alpha,j-1} \\ &\quad + \frac{Z^n C^{a,n}_{\alpha,j-1} F}{R_g T} \left(\psi^a_{\alpha,j} - \psi^a_{\alpha,j-1} \right) \Big] \\ &\quad + C^{a,n}_{\alpha,j-1} Q^{a,ax}_{in,\alpha,j}. \end{aligned} \tag{S14}$$

$k^{a,rad,n}$ ($k^{a,ax,n}$) is the radial (axial) diffusive permeability of ion n in the apoplast, Z^n is the valence of ion n, ψ^a is the electric potential in the apoplast and F is Faraday's constant (96 485 C mol^{-1}).

In a similar manner, the radial flux of ion n through the symplast via the plasmodesmata, $S^{s,rad,n}_{in,\alpha,j}$, is given by,

$$\begin{aligned} S^{s,rad,n}_{in,\alpha,j} &= -k^{s,n} A^{s,rad}_{\alpha,j} \Big[C^{c,n}_{\alpha,j} - C^{c,n}_{\alpha-1,j} \\ &\quad + \frac{Z^n C^{c,n}_{\alpha-1,j} F}{R_g T} \left(\psi^c_{\alpha,j} - \psi^c_{\alpha-1,j} \right) \Big] \\ &\quad + \left(1 - \sigma^{s,n}\right) C^{c,n}_{\alpha-1,j} Q^{s,rad}_{in,\alpha,j}, \end{aligned} \tag{S15}$$

while the axial flux of ion n through the symplast via the plasmodesmata, $S^{s,ax,n}_{in,\alpha,j}$, is given by,

$$\begin{aligned} S^{s,ax,n}_{in,\alpha,j} &= -k^{s,n} A^{s,ax}_{\alpha,j-1} \Big[C^{c,n}_{\alpha,j} - C^{c,n}_{\alpha,j-1} \\ &\quad + \frac{Z^n C^{c,n}_{\alpha,j-1} F}{R_g T} \left(\psi^c_{\alpha,j} - \psi^c_{\alpha,j-1} \right) \Big] \\ &\quad + \left(1 - \sigma^{s,n}\right) C^{c,n}_{\alpha,j-1} Q^{s,ax}_{in,\alpha,j}, \end{aligned} \tag{S16}$$

where $k^{s,n}$ is the diffusive permeability of ion n in the symplast and ψ^c is the cytoplasmic electric potential.

Note that similar to the symplastic water flows, $S^{s,rad,n}_{in,\alpha,j} = 0$ in the outermost tissue region and the innermost tissue region at the location of functional xylem and $S^{s,ax,n}_{in,\alpha,j} = 0$ for the model elements at the bottom of the root since there are no connecting cells.

The radial outflow of ion n from the apoplast of an element is equal to the radial apoplastic inflow into its neighbor element,

$$S^{a,rad,n}_{out,\alpha,j} = S^{a,rad,n}_{in,\alpha+1,j} \tag{S17}$$

and similarly the radial outflow of ion n from the symplast of the cytoplasms in an element is equal to the radial symplastic inflow into the cytoplasms in its neighbor element,

$$S^{s,rad,n}_{out,\alpha,j} = S^{s,rad,n}_{in,\alpha+1,j}. \tag{S18}$$

The axial outflow of ion n from the apoplast of an element is equal to the axial apoplastic inflow into its neighbor,

$$S^{a,ax,n}_{out,\alpha,j} = S^{a,ax,n}_{in,\alpha,j+1}, \tag{S19}$$

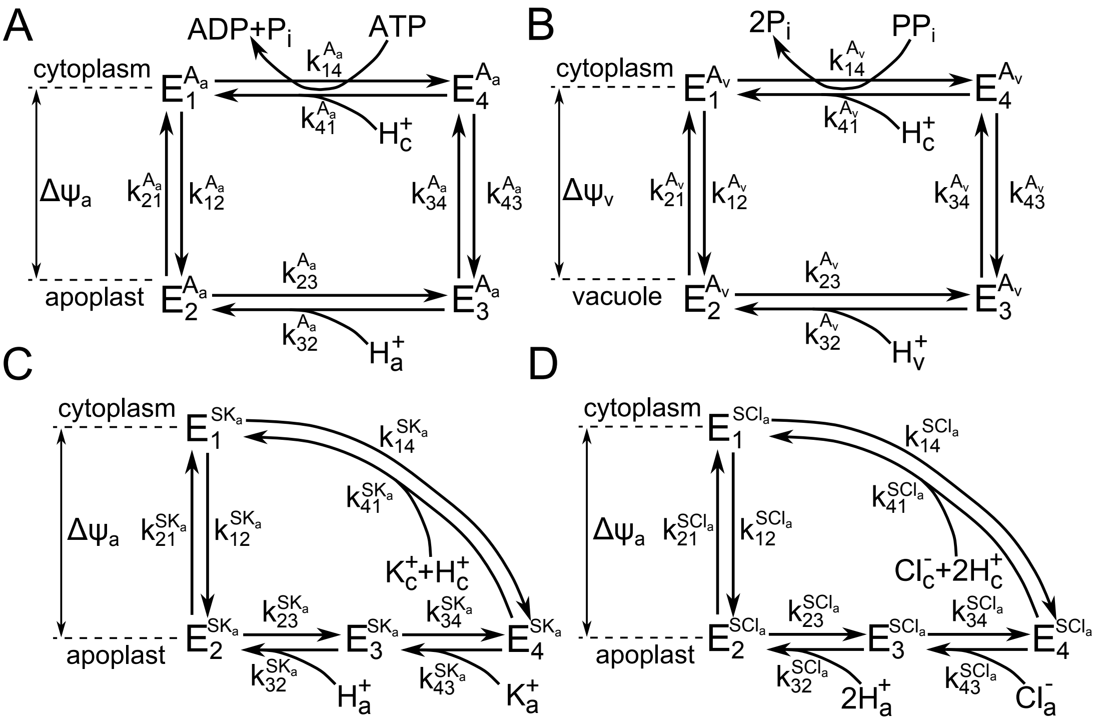

Figure S1. A schematic of the four state carrier cycles used to model (A) the plasma membrane H^+ pump, (B) the tonoplast H^+ pump, (C) the K^+/H^+ plasma membrane symporter, and (D) the Cl^-/H^+ plasma membrane symporter. E's refer to carrier states, while k's refer to rate constants. Adapted from Hills et al. (2012) Supplementary Material Figure 1.

and similarly the axial outflow of ion n from the symplast of the cytoplasms in an element is equal to the axial symplastic inflow into the cytoplasms in its neighbor element,

$$S^{s,ax,n}_{out,\alpha,j} = S^{s,ax,n}_{in,\alpha,j+1}. \tag{S20}$$

S.1.3 Governing equations of membrane transport of ions

The equations representing the membrane transport of ions in this model are identical to those developed for our single cell model (see Foster and Miklavcic (2015), Supplementary Data) with the exception that in this model several of the transport parameters can vary with cell type and location. For clarity and convenience the relevant equations are briefly described here, showing the parameters which may depend on (α, j). For a more detailed description of model assumptions see Foster and Miklavcic (2015), Supplementary Data.

The plasma membrane and tonoplast H^+ pumps, K^+/H^+ symporter and Cl^-/H^+ symporter are modeled by four state carrier cycles (Hills et al., 2012) as shown in Figure S1.

S.1.3.1 Primary active transport: H^+ pumps

The flux of each H^+ through the plasma membrane (tonoplast) pump, $J^{A_a}_H$ ($J^{A_v}_H$), is given by,

$$J^{A_{a/v}}_{H:\alpha,j} = \frac{N^{A_{a/v}}_{\alpha,j} A^{i,p/t}_{\alpha,j}}{N_A D^{A_{a/v}}_{\alpha,j}} \Big(k^{A_{a/v}}_{12:\alpha,j} k^{A_{a/v}}_{23} k^{A_{a/v}}_{34} k^{A_{a/v}}_{41:\alpha,j}$$
$$- k^{A_{a/v}}_{14} k^{A_{a/v}}_{21:\alpha,j} k^{A_{a/v}}_{32:\alpha,j} k^{A_{a/v}}_{43} \Big) \tag{S21}$$

where the denominator term, $D^{A_{a/v}}$ is given by,

$$\begin{aligned} D^{A_{a/v}}_{\alpha,j} &= k^{A_{a/v}}_{14} k^{A_{a/v}}_{21:\alpha,j} k^{A_{a/v}}_{32:\alpha,j} + k^{A_{a/v}}_{12:\alpha,j} k^{A_{a/v}}_{23} k^{A_{a/v}}_{34} \\ &+ k^{A_{a/v}}_{14} k^{A_{a/v}}_{21:\alpha,j} k^{A_{a/v}}_{34} + k^{A_{a/v}}_{14} k^{A_{a/v}}_{23} k^{A_{a/v}}_{34} \\ &+ k^{A_{a/v}}_{12:\alpha,j} k^{A_{a/v}}_{23} k^{A_{a/v}}_{41:\alpha,j} + k^{A_{a/v}}_{12:\alpha,j} k^{A_{a/v}}_{23} k^{A_{a/v}}_{43} \\ &+ k^{A_{a/v}}_{14} k^{A_{a/v}}_{21:\alpha,j} k^{A_{a/v}}_{43} + k^{A_{a/v}}_{14} k^{A_{a/v}}_{23} k^{A_{a/v}}_{43} \\ &+ k^{A_{a/v}}_{12:\alpha,j} k^{A_{a/v}}_{32:\alpha,j} k^{A_{a/v}}_{41:\alpha,j} + k^{A_{a/v}}_{12:\alpha,j} k^{A_{a/v}}_{32:\alpha,j} k^{A_{a/v}}_{43} \\ &+ k^{A_{a/v}}_{12:\alpha,j} k^{A_{a/v}}_{34} k^{A_{a/v}}_{41:\alpha,j} + k^{A_{a/v}}_{14} k^{A_{a/v}}_{32:\alpha,j} k^{A_{a/v}}_{43} \\ &+ k^{A_{a/v}}_{21:\alpha,j} k^{A_{a/v}}_{32:\alpha,j} k^{A_{a/v}}_{41:\alpha,j} + k^{A_{a/v}}_{21:\alpha,j} k^{A_{a/v}}_{32:\alpha,j} k^{A_{a/v}}_{43} \\ &+ k^{A_{a/v}}_{21:\alpha,j} k^{A_{a/v}}_{34} k^{A_{a/v}}_{41:\alpha,j} + k^{A_{a/v}}_{23} k^{A_{a/v}}_{34} k^{A_{a/v}}_{41:\alpha,j}. \end{aligned} \tag{S22}$$

N_A is the Avogadro constant (6.022×10^{23} mol^{-1}), $A^{i,p/t}$ is the initial area of the plasma/tonoplast membrane (assuming that shrinkage and expansion of the membranes due to changes in osmotic pressure gradients does not change the number of transporters present in the membranes), $N^{A_{a/v}}$ is the pump density and the k's are the reaction rates as shown in Figures S1A and S1B. The influence of $C^{c,H}$ and $C^{a/v,H}$ on the binding and unbinding are incorporated into $k^{A_{a/v}}_{41}$ and $k^{A_{a/v}}_{32}$, respectively,

$$k^{A_{a/v}}_{41:\alpha,j} = k^{A_{a/v},0}_{41} C^{c,H}_{\alpha,j} \tag{S23}$$

$$k^{A_{a/v}}_{32:\alpha,j} = k^{A_{a/v},0}_{32} C^{a/v,H}_{\alpha,j} \tag{S24}$$

where $k^{A_{a/v},0}_{41}$ ($k^{A_{a/v},0}_{32}$) is the value of $k^{A_{a/v}}_{41}$ ($k^{A_{a/v}}_{32}$) when $C^{c,H}$ ($C^{a/v,H}$) is 1mM. In addition, assuming that the transport across the membrane is the part of the cycle that depends on the electric potential (Hills et al., 2012), $k^{A_{a/v}}_{12}$ and $k^{A_{a/v}}_{21}$ are functions of the plasma/tonoplast transmembrane potential difference, $\Delta\psi^{p/t} = \psi^c - \psi^{a/v}$. Assuming a symmetric Eyring barrier and uncharged empty carrier (Hansen et al., 1981),

$$k^{A_{a/v}}_{12:\alpha,j} = k^{A_{a/v},0}_{12} \exp\left(\frac{F\Delta\psi^{p/t}_{\alpha,j}}{2R_gT}\right) \tag{S25}$$

$$k^{A_{a/v}}_{21:\alpha,j} = k^{A_{a/v},0}_{21} \exp\left(\frac{-F\Delta\psi^{p/t}_{\alpha,j}}{2R_gT}\right) \tag{S26}$$

where $k^{A_{a/v},0}_{12}$ and $k^{A_{a/v},0}_{21}$ are the values of the rate constants at zero transmembrane potential.

Only one tonoplast H^+ pump has been assumed even though we acknowledge that both a H^+-PPase and a V-type H^+-ATPase pump operate on the tonoplast of plant cell vacuoles. Regrettably, the available experimental data used to fit the model parameters cannot discriminate between the contributions of these two vacuolar H^+ pumps. Hence, since both pumps operate in the same direction, the explicit inclusion of a single

pump represents the function of both, with parameter values being representative of the combined effect of the two. We have chosen to refer to the pump as the H$^+$-PPase rather than the V-type H$^+$-ATPase because the former type has been shown to be important for salinity tolerance. To be specific, the overexpresssion of the H$^+$-PPase has been shown to increase salinity tolerance Brini et al. (2007), whereas this has not been demonstrated for the V-type H$^+$-ATPase. We do acknowledge that by keeping with a simplified description and not explicitly including both pumps we are unable to study the interaction between the tonoplast H$^+$-ATPase and the H$^+$-PPase.

S.1.3.2 Secondary active transport: symporters

In plant cells, the K$^+$/H$^+$ symporter has a transport ratio of approximately one K$^+$ per H$^+$ transported (Maathuis et al., 1997). Hence, the fluxes of K$^+$ ($J_K^{SK_a}$) and H$^+$ ($J_H^{SK_a}$) through the symporter are equal, $J_H^{SK_a} = J_K^{SK_a} = J^{SK_a}$. Similarly to the flux through H$^+$ pumps, the flux of each ion through the plasma membrane K$^+$/H$^+$ symporter, J^{SK_a}, is given by,

$$\begin{aligned} J_{\alpha,j}^{SK_a} = \frac{N_{\alpha,j}^{SK_a} A_{\alpha,j}^{i,p}}{N_A D_{\alpha,j}^{SK_a}} \Big(& k_{12:\alpha,j}^{SK_a} k_{23}^{SK_a} k_{34}^{SK_a} k_{41:\alpha,j}^{SK_a} \\ & - k_{14}^{SK_a} k_{21:\alpha,j}^{SK_a} k_{32:\alpha,j}^{SK_a} k_{43:\alpha,j}^{SK_a} \Big) \end{aligned} \tag{S27}$$

where the denominator term, D^{SK_a} is given by,

$$\begin{aligned} D_{\alpha,j}^{SK_a} = & k_{14}^{SK_a} k_{21:\alpha,j}^{SK_a} k_{32:\alpha,j}^{SK_a} + k_{12:\alpha,j}^{SK_a} k_{23}^{SK_a} k_{34}^{SK_a} \\ & + k_{14}^{SK_a} k_{21:\alpha,j}^{SK_a} k_{34}^{SK_a} + k_{14}^{SK_a} k_{23}^{SK_a} k_{34}^{SK_a} \\ & + k_{12:\alpha,j}^{SK_a} k_{23}^{SK_a} k_{41:\alpha,j}^{SK_a} + k_{12:\alpha,j}^{SK_a} k_{23}^{SK_a} k_{43:\alpha,j}^{SK_a} \\ & + k_{14}^{SK_a} k_{21:\alpha,j}^{SK_a} k_{43:\alpha,j}^{SK_a} + k_{14}^{SK_a} k_{23}^{SK_a} k_{43:\alpha,j}^{SK_a} \\ & + k_{12:\alpha,j}^{SK_a} k_{32:\alpha,j}^{SK_a} k_{41:\alpha,j}^{SK_a} + k_{12:\alpha,j}^{SK_a} k_{32:\alpha,j}^{SK_a} k_{43:\alpha,j}^{SK_a} \\ & + k_{12:\alpha,j}^{SK_a} k_{34}^{SK_a} k_{41:\alpha,j}^{SK_a} + k_{14}^{SK_a} k_{32:\alpha,j}^{SK_a} k_{43:\alpha,j}^{SK_a} \\ & + k_{21:\alpha,j}^{SK_a} k_{32:\alpha,j}^{SK_a} k_{41:\alpha,j}^{SK_a} + k_{21:\alpha,j}^{SK_a} k_{32:\alpha,j}^{SK_a} k_{43:\alpha,j}^{SK_a} \\ & + k_{21:\alpha,j}^{SK_a} k_{34}^{SK_a} k_{41:\alpha,j}^{SK_a} + k_{23}^{SK_a} k_{34}^{SK_a} k_{41:\alpha,j}^{SK_a}. \end{aligned} \tag{S28}$$

N^{SK_a} is the number of K$^+$/H$^+$ symporters per unit area of plasma membrane and the rate constants (k^{SK_a}'s) are shown in Figure S1C. In particular, the binding and unbinding rate constants are functions of the relevant ion concentrations,

$$k_{32:\alpha,j}^{SK_a} = k_{32}^{SK_a,0} C_{\alpha,j}^{a,H} \tag{S29}$$

$$k_{43:\alpha,j}^{SK_a} = k_{43}^{SK_a,0} C_{\alpha,j}^{a,K} \tag{S30}$$

$$k_{41:\alpha,j}^{SK_a} = k_{41}^{SK_a,0} C_{\alpha,j}^{c,K} C_{\alpha,j}^{c,H} \tag{S31}$$

where $k^{SK_a,0}$ is the value of k^{SK_a} when the relevant ion concentration/s are 1mM. In addition, assuming the transport of the ions across the membrane is the part of the cycle that depends on the electric potential (Blatt et al., 1987) $k_{12}^{SK_a}$ and $k_{21}^{SK_a}$ are functions of the plasma membrane electric potential difference, $\Delta\psi^p$.

Assuming a symmetric Eyring barrier and uncharged empty carrier (Hansen et al., 1981),

$$k^{SK_a}_{12:\alpha,j} = k^{SK_a,0}_{12} \exp\left(\frac{F\Delta\psi^p_{\alpha,j}}{R_gT}\right) \tag{S32}$$

$$k^{SK_a}_{21:\alpha,j} = k^{SK_a,0}_{21} \exp\left(\frac{-F\Delta\psi^p_{\alpha,j}}{R_gT}\right) \tag{S33}$$

where $k^{SK_a,0}_{12}$ and $k^{SK_a,0}_{21}$ are the values of the rate constants at zero plasma membrane electric potential difference ($\Delta\psi^p = 0$).

The Cl^-/H^+ symporter has a transport ratio of approximately one Cl^- per two H^+ transported (Beilby and Walker, 1981; Felle, 1994). Therefore the flux of H^+ through the Cl^-/H^+ symport ($J^{SCl_a}_{H}$) is twice the flux of Cl^- ($J^{SCl_a}_{Cl}$). These fluxes are calculated as above, with the binding and unbinding rate constants given by,

$$k^{SCl_a}_{32:\alpha,j} = k^{SCl_a,0}_{32}\left(C^{a,H}_{\alpha,j}\right)^2 \tag{S34}$$

$$k^{SCl_a}_{43:\alpha,j} = k^{SCl_a,0}_{43} C^{a,Cl}_{\alpha,j} \tag{S35}$$

$$k^{SCl_a}_{41:\alpha,j} = k^{SCl_a,0}_{41} C^{c,Cl}_{\alpha,j}\left(C^{c,H}_{\alpha,j}\right)^2 \tag{S36}$$

and the electric potential dependent kinetic constants given by,

$$k^{SCl_a}_{12:\alpha,j} = k^{SCl_a,0}_{12} \exp\left(\frac{F\Delta\psi^p_{\alpha,j}}{2R_gT}\right) \tag{S37}$$

$$k^{SCl_a}_{21:\alpha,j} = k^{SCl_a,0}_{21} \exp\left(\frac{-F\Delta\psi^p_{\alpha,j}}{2R_gT}\right) \tag{S38}$$

S.1.3.3 Secondary active transport: antiporters

The flux of Na^+ ions through antiporters, $J^{XNa_{a/v}}_{Na}$, is given by,

$$J^{XNa_{a/v}}_{Na:\alpha,j} = A^{i,p/t}_{\alpha,j} k^{XNa_{a/v}}_{\alpha,j}\Big(C^{c,Na}_{\alpha,j} C^{a/v,H}_{\alpha,j} - C^{a/v,Na}_{\alpha,j} C^{c,H}_{\alpha,j}\Big) \tag{S39}$$

Similarly, the flux of K^+ through the tonoplast K^+/H^+ antiporter ($J^{XK_v}_{K}$) is given by,

$$J^{XK_v}_{K:\alpha,j} = A^{i,t} k^{XK_v}_{\alpha,j}\left(C^{c,K}_{\alpha,j} C^{v,H}_{\alpha,j} - C^{v,K}_{\alpha,j} C^{c,H}_{\alpha,j}\right) \tag{S40}$$

Note that Eqs. (S39) and (S40) cannot be used to model processes such as carrier saturation as they do not consider binding and unbinding processes (Sukhov and Vodeneev, 2009).

The fluxes of Na^+ and H^+ ions due to the Na^+/H^+ antiporter are equal in magnitude, but opposite in direction. That is,

$$J^{XNa}_{H:\alpha,j} = -J^{XNa}_{Na:\alpha,j} \tag{S41}$$

Similarly for the K^+/H^+ antiporter,

$$J^{XK_v}_{H:\alpha,j} = -J^{XK_v}_{K:\alpha,j} \tag{S42}$$

S.1.3.4 Passive transport

The passive flux of ions ($S = Na^+$, K^+ and Cl^-) through voltage insensitive channels (VICs) is modeled using the Goldman-Hodgkin-Katz (GHK) current equation (Keener and Sneyd, 2009),

$$\begin{aligned} J^{C_{a/v}}_{S:\alpha,j} =& \frac{A^{i,p/t}_{\alpha,j} P^{S_{a/v}}_{\alpha,j} Z^S F \Delta\psi^{p/t}_{\alpha,j}}{R_g T} \\ & \times \left[\frac{C^{c,S}_{\alpha,j} - C^{a/v,S}_{\alpha,j} \exp\left(\frac{-Z^S \Delta\psi^{p/t}_{\alpha,j} F}{R_g T} \right)}{1 - \exp\left(\frac{-Z^S \Delta\psi^{p/t}_{\alpha,j} F}{R_g T} \right)} \right] \end{aligned} \tag{S43}$$

where P^S is the permeability of the channel for each specific ion S expressed as per unit area of membrane.

The fluxes of K^+ through voltage dependent channels are also modeled using the GHK current equation, with the inclusion of a term representing the voltage dependence of the probability of the channels being open. Voltage gating of Na^+ and Cl^- is not modeled as uptake of Na^+ into plant cells via plasma membrane ion channels is thought to be predominantly via voltage insensitive non-selective cation channels (Kavitha et al., 2012; Kronzucker and Britto, 2011). With limited experimental data available for tonoplast channels it is difficult to properly model voltage gating. This is discussed further in Foster and Miklavcic (2015), Supplementary Data.

Hence, the fluxes of K^+ through IRCs, $J^{IRC_a}_K$, and ORCs, $J^{ORC_a}_K$, are given by,

$$\begin{aligned} J^{IRC_a/ORC_a}_{K:\alpha,j} =& \frac{A^{i,p}_{\alpha,j} P^{IRC/ORC}_{o:\alpha,j} P^{IRC/ORC_a}_{\alpha,j} F \Delta\psi^{p}_{\alpha,j}}{R_g T} \\ & \times \left[\frac{C^{c,K}_{\alpha,j} - C^{a,K}_{\alpha,j} \exp\left(\frac{-\Delta\psi^{p}_{\alpha,j} F}{R_g T} \right)}{1 - \exp\left(\frac{-\Delta\psi^{p}_{\alpha,j} F}{R_g T} \right)} \right] \end{aligned} \tag{S44}$$

where $P^{IRC/ORC}_o$ is the IRC/ORC open probability (or fraction of IRCs/ORCs that are open) and $P^{IRC/ORC}_a$ is the maximal IRC/ORC permeability. Assuming two state channels (open/closed) the open probabilities are

given by (Chowdhury and Chanda, 2012),

$$P^{IRC}_{o:\alpha,j} = \frac{1}{1 + \exp\left[\frac{FZ^{IRC}_g}{R_g T}\left(\Delta\psi^p_{\alpha,j} - \Delta\psi^{IRC_a}_{50}\right)\right]} \tag{S45}$$

$$P^{ORC}_{o:\alpha,j} = \frac{1}{1 + \exp\left[-\frac{FZ^{ORC}_g}{R_g T}\left(\Delta\psi^p_{\alpha,j} - \Delta\psi^{ORC_a}_{50}\right)\right]} \tag{S46}$$

where Z_g is the IRC/ORC gating charge and $\Delta\psi^{IRC_a/ORC_a}_{50}$ is the plasma membrane transmembrane potential at which half the inward/outward rectifying channels are open.

S.1.4 Apoplastic binding to fixed charges

The binding reactions representing the binding of cations to fixed anionic charges in the apoplast can be represented by,

$$H^+_a + B^-_a \underset{k^H_{dn}}{\overset{k^H_{an}}{\rightleftarrows}} HB_a$$

$$Na^+_a + B^-_a \underset{k^{Na}_{dn}}{\overset{k^{Na}_{an}}{\rightleftarrows}} NaB_a$$

$$K^+_a + B^-_a \underset{k^K_{dn}}{\overset{k^K_{an}}{\rightleftarrows}} KB_a, \tag{S47}$$

where k^n_{an} (k^n_{dn}) represent the association (dissociation) reaction rates. Note that the extent of monovalent cation may be overestimated by the exclusion of divalent cations such as Ca^{2+} from the model. The net rate of cation dissociation, $R^{bind,n}_{\alpha,j}$, is given by,

$$R^{bind,H}_{\alpha,j} = k^H_{dn} C^{a,HB}_{\alpha,j} - k^H_{an} C^{a,H}_{\alpha,j} C^{a,B}_{\alpha,j} \tag{S48}$$

$$R^{bind,Na}_{\alpha,j} = k^{Na}_{dn} C^{a,NaB}_{\alpha,j} - k^{Na}_{an} C^{a,Na}_{\alpha,j} C^{a,B}_{\alpha,j} \tag{S49}$$

$$R^{bind,K}_{\alpha,j} = k^K_{dn} C^{a,KB}_{\alpha,j} - k^K_{an} C^{a,K}_{\alpha,j} C^{a,B}_{\alpha,j} \tag{S50}$$

$$R^{bind,Cl}_{\alpha,j} = 0 \tag{S51}$$

where $C^{a,B}_{\alpha,j}$ is the concentration of anionic charges which have not bound cations. Note that this approach does not incorporate competitive binding (Miklavic and Ninham, 1990; Miklavcic et al., 2014). The total number of binding sites in the apoplast of each (α, j) element remains fixed. Due to the assumption of constant apoplastic volume the total amount of binding sites expressed as mol m^{-3} ($B^T_{\alpha,j}$) is constant and is given by,

$$C^{a,HB}_{\alpha,j} + C^{a,NaB}_{\alpha,j} + C^{a,KB}_{\alpha,j} + C^{a,B}_{\alpha,j} = B^T_{\alpha,j} = \text{constant} \tag{S52}$$

S.1.5 Buffering

Due to the presence of buffering ions in the cytoplasm and in the vacuole, the change in *free* H^+ ion concentration in the cytoplasm and vacuole is not given just by Eqs. (12) and (14), respectively. Instead, when H^+ is transported into or out of a cell compartment the actual change in free concentration of H^+

is less than the amount transported due to chemical interactions with buffer anions (B^-) leading to the formation/dissociation of a weak acid (HB). This buffering reaction is represented by

$$H^+_{c/v} + B^-_{c/v} \rightleftharpoons HB_{c/v} \tag{S53}$$

where $B^-_{c/v}$ models the combined contribution of all (univalent) buffering anions in the cytoplasm/vacuole. As in Foster and Miklavcic (2015), the concept of a constant buffering capacity ($\beta_{c/v}$) is used to model all buffering effects in the cytoplasms and vacuoles (irrespective of pK_a value) (Harris, 2007),

$$\beta^{c/v}_{\alpha,j} = -\frac{dC^{c/v,H}_{\alpha,j}}{dpH^{c/v}_{\alpha,j}} \Rightarrow \frac{dpH^{c/v}_{\alpha,j}}{dt} = -\frac{1}{\beta^{c/v}_{\alpha,j}}\frac{dC^{c/v,H}_{\alpha,j}}{dt}. \tag{S54}$$

Here, $dC^{c/v,H}_{\alpha,j}/dt$ refers to the (unbuffered) change in H^+ concentration due to transmembrane and symplastic fluxes, found from Eqs. (12) and (14), and $pH^{c/v}_{\alpha,j}$ is the buffered pH which can be used to calculate the concentration of free H^+, $C^{c/v,H_f}_{\alpha,j} = 1000 \times 10^{-pH^{c/v}_{\alpha,j}}$ mM. Assuming that the buffering anions are unable to be transported between cell compartments or via the plasmodesmata, the calculation of the concentration of the buffering anions in each cell cytoplasm/vacuole ($C^{c/v,B}_{\alpha,j}$) is unchanged from our earlier single cell model (Foster and Miklavcic, 2015). Hence, $C^{c/v,B}_{\alpha,j}$ is calculated using,

$$\frac{d}{dt}\left(V^{c/v}_{\alpha,j}C^{c/v,B}_{\alpha,j}\right) = \frac{d}{dt}\left(V^{c/v}_{\alpha,j}C^{c/v,H_f}_{\alpha,j}\right) - \frac{d}{dt}\left(V^{c/v}_{\alpha,j}C^{c/v,H}_{\alpha,j}\right). \tag{S55}$$

In each cytoplasm and vacuole compartment the total amount of buffering anion and weak acid contributing to the osmotic pressure is constant and hence determined by initial conditions.

S.1.6 Electric potential calculations

The assumption of electroneutrality in each apoplastic, cytoplasmic and vacuolar compartment can be represented by the following expression for all (α, j),

$$C^{a,H}_{\alpha,j} + C^{a,Na}_{\alpha,j} + C^{a,K}_{\alpha,j} - C^{a,Cl}_{\alpha,j} - C^{a,B}_{\alpha,j} = 0, \tag{S56}$$

$$C^{c,H}_{\alpha,j} + C^{c,Na}_{\alpha,j} + C^{c,K}_{\alpha,j} - C^{c,Cl}_{\alpha,j} - C^{c,B}_{\alpha,j} = 0, \tag{S57}$$

$$C^{v,H}_{\alpha,j} + C^{v,Na}_{\alpha,j} + C^{v,K}_{\alpha,j} - C^{v,Cl}_{\alpha,j} - C^{v,B}_{\alpha,j} = 0. \tag{S58}$$

Multiplying each of the above equations by its corresponding compartment volume ($V^a_{\alpha,j}$, $V^c_{\alpha,j}$ and $V^v_{\alpha,j}$, respectively), differentiating with respect to time, substituting in conservation equations (Eqs. (9), (12) and (14), as well as the buffering anion conservation equations), converting fluxes to currents and using the fact that fluxes through antiporters are equal and opposite (Eqs. (S41) and (S42)), leads to the following set of

zero net current conditions,

$$
\begin{aligned}
I^{a,tot}_{\alpha,j} &= I^{a,rad}_{in,\alpha,j} - I^{a,rad}_{out,\alpha,j} + I^{a,ax}_{in,\alpha,j} - I^{a,ax}_{out,\alpha,j} + N^{cell}_{\alpha,j} I^{p}_{\alpha,j} \\
&= 0, \qquad \text{(S59)} \\
I^{s,tot}_{\alpha,j} &= I^{s,rad}_{in,\alpha,j} - I^{s,rad}_{out,\alpha,j} + I^{s,ax}_{in,\alpha,j} - I^{s,ax}_{out,\alpha,j} - N^{cells}_{\alpha,j} I^{p}_{\alpha,j} \\
&= 0, \qquad \text{(S60)} \\
I^{t}_{\alpha,j} &= 0. \qquad \text{(S61)}
\end{aligned}
$$

Here, $I^{a,tot}_{\alpha,j}$ is the total net current into each apoplastic compartment, which consists of a contribution from the radial apoplastic ion fluxes ($I^{a,rad}_{in,\alpha,j} = F\sum_{n=1}^{N} Z^n S^{a,rad,n}_{in,\alpha,j}$); the axial apoplastic fluxes ($I^{a,ax}_{in,\alpha,j} = F\sum_{n=1}^{N} Z^n S^{a,ax,n}_{in,\alpha,j}$); as well as the fluxes of ions across the plasma membranes of each cell in the apoplast of a given (α, j) ($I^{p}_{\alpha,j} = F\sum_{n=1}^{N} Z^n S^{p,n}_{in,\alpha,j}$). Note that N represents the total number of charged species under consideration, which may vary between compartments. Similarly, $I^{s,tot}_{\alpha,j}$ is the total net current into the cytoplasmic compartments in each (α, j), which consists of a contribution from the radial symplastic ion fluxes ($I^{s,rad}_{in,\alpha,j} = F\sum_{n=1}^{N} Z^n S^{s,rad,n}_{in,\alpha,j}$); a contribution from the axial symplastic ion fluxes ($I^{s,ax}_{in,\alpha,j} = F\sum_{n=1}^{N} Z^n S^{s,ax,n}_{in,\alpha,j}$); as well as a contribution from the plasma membrane ion fluxes ($I^{p}_{\alpha,j}$). $I^{t}_{\alpha,j}$ is the net current across each individual tonoplast in each (α, j) element, where $I^{t}_{\alpha,j} = F\sum_{n=1}^{N} Z^n S^{t,n}_{in,\alpha,j}$. Note that $I^{a,rad}_{out,\alpha,j} = I^{a,rad}_{in,\alpha+1,j}$; $I^{a,ax}_{out,\alpha,j} = I^{a,ax}_{in,\alpha,j+1}$ and similarly $I^{s,rad}_{out,\alpha,j} = I^{s,rad}_{in,\alpha+1,j}$; $I^{s,ax}_{out,\alpha,j} = I^{s,ax}_{in,\alpha,j+1}$. The fluxes represented in Eqs. (S59) to (S61) are nonlinear functions of the electric potential differences between the different model compartments (see Section S.1.2 and S.1.3). Hence, Eqs. (S59) to (S61) represent a system of nonlinear equations, which is solved to find $\psi^{a}_{\alpha,j}$, $\psi^{c}_{\alpha,j}$ and $\psi^{v}_{\alpha,j}$.

S.2. ADDITIONAL DETAILS OF PARAMETER SELECTION

In addition to the membrane transport parameters discussed in Section 3.4, there are several water and solute transport parameters that are required to describe transport in the symplast and apoplast. These are summarized in Table S1 and include solute permeabilities ($k^{a,rad/ax,n}$, $k^{s,n}$), solute reflection coefficients ($\sigma^{s,n}$) and water permeabilities ($L^{a,rad/ax}_{p}$, L^{s}_{p}).

The various solute permeabilities for each ion, n, were calculated using,

$$
k^{a/s,rad/ax,n} = \frac{D^{a/s,n}_{e}}{\text{Transport path length}} \qquad \text{(S62)}
$$

where $D^{a,n}_{e}$ ($D^{s,n}_{e}$) is the effective diffusion coefficient of ion n in the apoplast (symplast). As discussed below, different approaches were used to determine D^{n}_{e} in the apoplast and symplast based on the availability of relevant data. The relevant transport path lengths were: thickness of each tissue region, d_α, for $k^{a,rad}_{\alpha,j}$; height of each model element, l^{ax}_{j}, for $k^{a,ax}_{\alpha,j}$; and cell wall thickness, t_{cw}, (*i.e.*, the length of the plasmodesmata) for k^{s}.

The apoplast is a porous medium, with properties that have not been well characterized experimentally. Hence, experimentally determined values for $D^{a,n}_{e}$ were used rather than theoretically determined values based on porous medium properties. There is a lack of experimental data on the apoplastic diffusion coefficients for the solutes of interest to this model (particularly Na^+). In contrast, there has been quite

Table S1. Summary of apoplastic and symplastic transport parameters used and their source. Cell membrane transport parameters are as specified in Table S2. Effective apoplastic diffusion coefficients were calculated using bulk diffusion coefficients from Haynes (2015) and $D_e^{a,n} = \frac{D^n}{5}$. Apoplastic water permeabilities were obtained from Zhu and Steudle (1991), while the symplastic water permeability was estimated using the Hagen-Poiseuille equation. The total number of fixed negative charges was obtained from Grignon and Sentenac (1991), with the remaining binding parameters assumed.

Parameter	Value	Units
**Apoplastic parameters**		
Effective apoplastic diffusion coefficients		
$D_e^{a,H}$	9.31×10^{-9}	$m^2\ s^{-1}$
$D_e^{a,Na}$	1.33×10^{-9}	$m^2\ s^{-1}$
$D_e^{a,K}$	1.96×10^{-9}	$m^2\ s^{-1}$
$D_e^{a,Cl}$	2.03×10^{-9}	$m^2\ s^{-1}$
Axial apoplastic water permeability, $L_{p:j}^{a,ax}$	$\left(2.5 \times 10^{-10}\right)/l_j^{ax}$	$m\ s^{-1}\ MPa^{-1}$
Radial apoplastic water permeability, $L_{p:\alpha}^{a,rad}$	$\left(2.5 \times 10^{-10}\right)/d_\alpha$	$m\ s^{-1}\ MPa^{-1}$
Total number of fixed negative charges, B^T	500	$mol\ m^{-3}$
Binding equilibrium constant for H^+, K_{bind}^{H}	1×10^4	mM^{-1}
Binding equilibrium constant for Na^+ and K^+, $K_{bind}^{Na,K}$	1	mM^{-1}
Binding association rate, k_{an}	1×10^{-12}	$m^6\ s^{-1}\ mol^{-1}$
**Symplastic parameters**		
Effective symplastic diffusion coefficients		
$D_e^{s,H}$	8.47×10^{-9}	$m^2\ s^{-1}$
$D_e^{s,Na}$	7.91×10^{-10}	$m^2\ s^{-1}$
$D_e^{s,K}$	1.35×10^{-9}	$m^2\ s^{-1}$
$D_e^{s,Cl}$	1.42×10^{-9}	$m^2\ s^{-1}$
Symplastic water permeability, L_p^s	1.63×10^{-3}	$m\ s^{-1}\ MPa^{-1}$
Symplastic reflection coefficients		
$\sigma^{s,H}$	0.00	
$\sigma^{s,Na}$	0.06	
$\sigma^{s,K}$	0.03	
$\sigma^{s,Cl}$	0.03	

a few experimental studies investigating the apoplastic diffusion coefficients of small organic molecules. Unfortunately, there is an enormous range in the reported measured values of D_n^a, extending from $\frac{D}{7}$ to $\frac{D}{1000}$, where D is the bulk diffusion coefficient of the relevant solute (Kramer et al., 2007). However, the values at the extreme low end of this range include the effects of the Casparian strip which blocks transport in the apoplast (Kramer et al., 2007). Since these barriers are explicitly simulated in this model, it is inappropriate to use values from the low end of the measured range as this would understate the rate of diffusion. Values of the order of $\frac{D}{10}$ have been attributed to the apoplastic diffusion coefficient of small organic molecules in the absence of any apoplastic barriers (Kramer et al., 2007). However, the ion species of interest in this model are smaller than the organic molecules for which the value $\frac{D}{10}$ has been determined. Hence, a slightly larger effective apoplastic diffusion coefficient of $\frac{D}{5}$ was used in this model. Unfortunately, the experimentally measured values include the effects of fixed negative anions which are present in the apoplast. Hence, the effects of these anions may be double counted in the model since their presence is explicitly modeled.

$L_p^{a,ax/rad}$ was also determined using experimentally measured apoplastic water conductivities. Apoplastic water conductivities ranging from 2.5×10^{-10} to 6.1×10^{-9} m^2 s^{-1} MPa^{-1} were measured by Zhu and Steudle (1991) for the various tissue types in maize roots. However, Michael et al. (1997) measured a significantly lower apoplastic water conductivity of 4.9×10^{-11} m^2 s^{-1} MPa^{-1} in the parenchyma of a potato storage organ, which was of a similar magnitude to values determined for Characean algae (Michael et al., 1997). Hence, a value at the low end of the range measured by Zhu and Steudle (1991) was used in this model. This conductivity was divided by the relevant path length to determine $L_p^{a,ax/rad}$ (see Table S1).

In the symplast, while there is a lack of experimentally measured diffusion coefficients for solute transport via plasmodesmata, data is available describing the geometry of plasmodesmata (Ehlers and Bel, 1999; Ehlers and Kollmann, 2001). Hence, the effective symplastic diffusion coefficient for each ion n was determined using $D_e^{s,n} = H^n D^n$ where H^n is the hindrance factor for diffusion of solute n (Liesche and Schulz, 2013). For a liquid-filled cylindrical pore (Dechadilok and Deen, 2006),

$$\begin{aligned} H^n = 1 &+ \frac{9}{8}\gamma_n \ln \gamma_n - 1.56034\gamma_n + 0.528155\gamma_n^2 \\ &+ 1.91521\gamma_n^3 - 2.81903\gamma_n^4 + 0.270788\gamma_n^5 \\ &+ 1.10115\gamma_n^6 - 0.435933\gamma_n^7, \end{aligned} \tag{S63}$$

where γ_n is the relative size of the solute radius (r_n) to the pore radius (r_p), $\gamma_n = \frac{r_n}{r_p}$. This hindrance factor incorporates the effects of steric restrictions and particle-wall hydrodynamic interactions, and covers the range of γ values relevant here ($0.01 \leq \gamma_n \leq 0.1$). The hydrodynamic radius of each solute species was calculated using the bulk diffusion coefficient (Haynes, 2015) and the Stokes-Einstein equation. The resulting effective diffusion coefficients for ions in the symplast are summarized in Table S1.

Similarly, the solute reflection coefficients for transport via the plasmodesmata were calculated using $\sigma^{s,n} = 1 - W_n$ (Liesche and Schulz, 2013), where W is the hindrance factor for convection. For a cylindrical pore (Dechadilok and Deen, 2006),

$$W_n = (1 - \gamma_n)\left(\frac{1 + 3.867\gamma_n - 1.907\gamma_n^2 - 0.834\gamma_n^3}{1 + 1.867\gamma_n - 0.741\gamma_n^2}\right). \tag{S64}$$

This gives $\sigma^{s,n}$ values which are close to zero (see Table S1) as expected for small solutes in the plasmodesmata (Anderson, 1976). Note that the symplastic reflection coefficients of the buffering components are 1 ($\sigma^{s,B} = \sigma^{s,HB} = 1$) since these solutes are assumed to be immobile.

The parameters relating to the binding of ions to the fixed negative charges in the apoplast are summarized in Table S1.

S.3. ROOT GEOMETRY

Values for $A_{\alpha,j}^{a/s,rad/ax}$, $A_{\alpha,j}^{p}$ and $A_{\alpha,j}^{t}$ were required for the water flow and ion flux calculations. As shown in Figure 1C, the cells were assumed to have an annular sector cross-section. The axial and radial distance between the cells in different (α, j) elements was assumed to equal the cell wall thickness. In contrast, the distance between cells in the *same* tissue region (t_α) differed between the tissue regions to ensure (despite the differing number of cells in each α tissue region, see Table S3) that the radial apoplastic and symplastic surface areas at the interfaces of the different tissue regions were consistent. t_α was calculated using, $t_\alpha = \frac{2\pi R_\alpha \theta}{360 N_{\alpha,j}^{cells}}$, where R_α is the outer radius of tissue region α (determined using the tissue region

thicknesses) and θ is a constant angle (6°) chosen to provide reasonable thickness values and a reasonable fraction of tissue volume occupied by the apoplast. The thicknesses of the different root tissue regions (d_α) are as outlined in Table S3.

Using the setup described above and shown in Figure 1 the radial apoplastic surface area is given by,

$$A^{a,rad}_{\alpha,j} = t_\alpha N^{cells}_{\alpha,j} l^{ax}_{\alpha,j} + 2\pi R_\alpha \left(1 - \frac{\theta}{360}\right) t_{cw}, \tag{S65}$$

and the axial apoplastic cross-sectional area is given by,

$$\begin{aligned} A^{a,ax}_{\alpha,j} = & \frac{\pi\theta\left(R^2_\alpha - R^2_{\alpha+1}\right)}{360} + \pi\left[R^2_\alpha - \left(R_\alpha - \frac{t_{cw}}{2}\right)^2\right]\left(1 - \frac{\theta}{360}\right) \\ & + \pi\left[\left(R_{\alpha+1} + \frac{t_{cw}}{2}\right)^2 - R^2_{\alpha+1}\right]\left(1 - \frac{\theta}{360}\right). \end{aligned} \tag{S66}$$

Note that in $\alpha = 5$ (the xylem) when living cells are present (*i.e.*, for $j < j_{DZ}$),

$$\begin{aligned} A^{a,ax}_{5,j<j_{DZ}} = & \frac{\pi\theta R^2_{\alpha=5}}{360} \\ & + \pi\left[R^2_{\alpha=5} - \left(R_{\alpha=5} - \frac{t_{cw}}{2}\right)^2\right]\left(1 - \frac{\theta}{360}\right), \end{aligned} \tag{S67}$$

while in the non-living sections of $\alpha = 5$ (*i.e.*, for $j \geq j_{DZ}$), $A^{a,ax}_{5,j\geq j_{DZ}} = \pi R^2_{\alpha=5}$.

The values of $A^{s,rad}_{\alpha,j}$ and $A^{s,ax}_{\alpha,j}$ were calculated by assuming that a given fraction of the connecting plasma membrane surface areas were occupied by plasmodesmata (F^{rad}_α and F^{ax}_α, respectively). F^{rad}_α and F^{ax}_α were estimated by assuming a cross-sectional area of 7.47×10^{-17} m^2 per plasmodesmata (Ehlers and Bel, 1999; Ehlers and Kollmann, 2001) and using the plasmodesmata frequency data for each interface between mature root cells in *Arabidopsis* roots measured by Zhu et al. (1998) (see Table S3). Hence, the radial symplastic surface area is given by,

$$A^{s,rad}_{\alpha,j} = 2\pi R_\alpha \left(1 - \frac{\theta}{360}\right)\left(l^{ax}_{\alpha,j} - t_{cw}\right) F^{rad}_\alpha, \tag{S68}$$

and similarly the axial symplastic cross-sectional area is given by,

$$\begin{aligned} A^{s,ax}_{\alpha,j} = \pi & \left[\left(R_\alpha - \frac{t_{cw}}{2}\right)^2 - \left(R_{\alpha+1} + \frac{t_{cw}}{2}\right)^2\right] \\ & \times \left(1 - \frac{\theta}{360}\right) F^{ax}_\alpha. \end{aligned} \tag{S69}$$

Note that in $\alpha = 5$ (the xylem) when living cells are present (*i.e.*, for $j < j_{DZ}$),

$$A^{s,ax}_{5,j<j_{DZ}} = \pi\left(R_{\alpha=5} - \frac{t_{cw}}{2}\right)^2\left(1 - \frac{\theta}{360}\right) F^{ax}_{\alpha=5}, \tag{S70}$$

while in the non-living sections of $\alpha = 5$ there are no symplastic connections.

The surface area of the plasma membranes in cells in $\alpha = 1, \ldots, 4$ is given by,

$$\begin{aligned}
A^p_{\alpha,j} = {} & \frac{2\pi}{N^{cells}_{\alpha,j}} \left[\left(R_\alpha - \frac{t_{cw}}{2} \right)^2 - \left(R_{\alpha+1} + \frac{t_{cw}}{2} \right)^2 \right] \\
& \times \left(1 - \frac{\theta}{360} \right) (1 - F^{ax}_\alpha) \\
& + \frac{2\pi}{N^{cells}_{\alpha,j}} \left(R_\alpha - \frac{t_{cw}}{2} \right) \left(1 - \frac{\theta}{360} \right) \\
& \times \left(l^{ax}_{\alpha,j} - t_{cw} \right) \left(1 - F^{rad}_\alpha \right) \\
& + \frac{2\pi}{N^{cells}_{\alpha,j}} \left(R_{\alpha+1} + \frac{t_{cw}}{2} \right) \left(1 - \frac{\theta}{360} \right) \\
& \times \left(l^{ax}_{\alpha,j} - t_{cw} \right) \left(1 - F^{rad}_{\alpha+1} \right) \\
& + 2 \left[\left(R_\alpha - \frac{t_{cw}}{2} \right) - \left(R_{\alpha+1} + \frac{t_{cw}}{2} \right) \right] \left(l^{ax}_{\alpha,j} - t_{cw} \right) . \qquad \text{(S71)}
\end{aligned}$$

Table S2: Summary of membrane transport parameters used and their source. Refer to Section S.1.3 for more detail about the H^+ pump and symporter kinetic parameters. Parameters were obtained using the fitting process described in Foster and Miklavcic (2015)**[a]** or were obtained from the following sources (as described in Foster and Miklavcic (2015)): George et al. (2007)**[b]**, Amtmann et al. (1999)**[c]**, Roberts and Tester (1995)**[d]**, Roberts and Tester (1995)**[e]**, Wegner et al. (1994)**[f]**, White and Lemtiri-Chlieh (1995)**[g]**, Chen et al. (2007)**[h]**,Ivashikina et al. (2001)**[i]**, Wegner and De Boer (1997)**[j]**, Hills et al. (2012)**[k]**.

**Parameter**	**Value**	**Units**
Plasma membrane water permeability, L_p^p		
Tonoplast water permeability, L_p^t		
Cytoplasmic buffering capacity, β_c**[a]**	23	mM/pH unit
Vacuolar buffering capacity, β_v**[a]**	30	mM/pH unit
Plasma membrane VIC permeability, P^{VIC_a}**[a]**	6.2×10^{-10}	$m\ s^{-1}$
Tonoplast Na^+ channel permeability, P^{Na_v}**[a]**	1.9×10^{-9}	$m\ s^{-1}$
Plasma membrane Cl^- channel permeability, P^{Cl_a}**[a]**	4.4×10^{-10}	$m\ s^{-1}$
Tonoplast Cl^- channel permeability, P^{Cl_v}**[a]**	1.4×10^{-9}	$m\ s^{-1}$
Plasma membrane K^+ IRC parameters**[c], [d], [e], [f], [g]**:		
Channel permeability, P_a^{IRC}	1×10^{-8}	$m\ s^{-1}$
Gating charge, Z_g^{IRC}	2	
Half activation potential, $\Delta\psi_{50}^{IRC_a}$	-140	mV
Plasma membrane K^+ ORC parameters**[h], [i], [j]**:		
Channel permeability, P^{ORC_a}	8×10^{-9}	$m\ s^{-1}$
Gating charge, Z_g^{ORC}	1.2	
Half activation potential, $\Delta\psi_{50}^{ORC_a}$	10	mV
Plasma membrane Na^+/H^+ antiporter reaction rate, k^{XNa_a}**[a]**	9.6×10^{-6}	$m^4\ mol^{-1}\ s^{-1}$

Continued on next page

Table S2 – continued from previous page

Parameter	Value	Units
Tonoplast Na^+/H^+ antiporter reaction rate, k^{XNa_v}[(a)]	5.7×10^{-8}	$m^4\ mol^{-1}\ s^{-1}$
Tonoplast K^+/H^+ antiporter reaction rate, k^{XK_v}[(k)]	1×10^{-11}	$m^4\ mol^{-1}\ s^{-1}$
Plasma membrane H^+ pump density, N^{A_a}[(a)]	2.1×10^{15}	m^{-2}
Plasma membrane H^+ pump kinetic parameters[(k)]:		
$k_{12}^{A_a,0}$	2×10^3	s^{-1}
$k_{21}^{A_a,0}$	2×10^2	s^{-1}
$k_{23}^{A_a}$	5×10^4	s^{-1}
$k_{32}^{A_a,0}$	1×10^5	$m^3\ mol^{-1}\ s^{-1}$
$k_{34}^{A_a}$	500	s^{-1}
$k_{43}^{A_a}$	10	s^{-1}
$k_{14}^{A_a}$	200	s^{-1}
$k_{41}^{A_a,0}$	2×10^6	$m^3\ mol^{-1}\ s^{-1}$
Tonoplast H^+ pump density, N^{A_v}	1.3×10^{16}[(a)]	m^{-2}
Tonoplast H^+ pump kinetic parameters[(k)]:		
$k_{12}^{A_v,0}$	1×10^3	s^{-1}
$k_{21}^{A_v,0}$	100	s^{-1}
$k_{23}^{A_v}$	1×10^3	s^{-1}
$k_{32}^{A_v,0}$	5×10^6	$m^3mol^{-1}\ s^{-1}$
$k_{34}^{A_v}$	1×10^{11}	s^{-1}
$k_{43}^{A_v}$	1×10^7	s^{-1}
$k_{14}^{A_v}$	1×10^4	s^{-1}
$k_{41}^{A_v,0}$	3×10^6	$m^3\ mol^{-1}\ s^{-1}$
Plasma membrane K^+/H^+ symporter parameters[(k)]:		
N_a^{SK}	8×10^{21}	m^{-2}
$k_{12}^{SK_a,0}$	2	s^{-1}
$k_{21}^{SK_a,0}$	0.4	s^{-1}
$k_{23}^{SK_a}$	1×10^4	s^{-1}
$k_{32}^{SK_a}$	1×10^9	$m^3\ mol^{-1}\ s^{-1}$
$k_{34}^{SK_a}$	1×10^5	s^{-1}
$k_{43}^{SK_a}$	1×10^7	$m^3\ mol^{-1}\ s^{-1}$
$k_{14}^{SK_a}$	50	s^{-1}
$k_{41}^{SK_a}$	1×10^8	$m^6\ mol^{-2}\ s^{-1}$
Plasma membrane Cl^-/H^+ symporter parameters[(k)]:		
N_a^{SCl}	3×10^{24}	m^{-2} (assumed)
$k_{12}^{SCl_a,0}$	1×10^3	s^{-1}
$k_{21}^{SCl_a,0}$	50	s^{-1}
$k_{23}^{SCl_a}$	100	s^{-1}
$k_{32}^{SCl_a}$	1×10^{15}	$m^6\ mol^{-2}\ s^{-1}$
$k_{34}^{SCl_a}$	5×10^4	s^{-1}
$k_{43}^{SCl_a}$	100	$m^3\ mol^{-1}\ s^{-1}$

Continued on next page

Table S2 – continued from previous page

Parameter	Value	Units
$k_{14}^{SCl_a}$	100	s^{-1}
$k_{41}^{SCl_a}$	1×10^{12}	m^9 mol^{-3} s^{-1}

Table S3. Summary of root geometry parameters used and their source. Cell numbers were obtained from Dolan et al. (1993); tissue thicknesses were obtained from Casimiro et al. (2003), Dolan et al. (1993), Javot et al. (2003), Mattsson et al. (1999) and Scheres et al. (1995); the cell wall thickness was obtained from Kramer et al. (2007); the plasmodesmata pore radius was obtained from Ehlers and Bel (1999) and Ehlers and Kollmann (2001); and the plasmodesmata frequency was obtained from Zhu et al. (1998).

Parameter	Value
Number of cells in each tissue, $N_{\alpha,j}^{cells}$	
Epidermis ($\alpha = 1$)	19
Cortex ($\alpha = 2$)	8
Endodermis ($\alpha = 3$)	8
Pericycle ($\alpha = 4$)	12
Undifferentiated xylem ($\alpha = 5, j < j_{DZ}$)	2
Thickness of each tissue, d_α	
Epidermis	15 μm
Cortex	20 μm
Endodermis	10 μm
Pericycle	5 μm
Xylem	3 μm
Cell wall thickness, t_{cw}	200 nm
Plasmodesmata pore radius, r_p	1.6 nm
Plasmodesmata frequency for radial tissue interfaces	
Cortex - epidermis	0.133 no. μm^{-2}
Endodermis - cortex	0.258 no. μm^{-2}
Vascular - endodermis	0.300 no. μm^{-2}
Vascular - vascular	0.350 no. μm^{-2}
Plasmodesmata frequency for axial tissue interfaces	
Epidermis	0.617 no. μm^{-2}
Cortex	0.508 no. μm^{-2}
Endodermis	0.310 no. μm^{-2}
Vascular	1.108 no. μm^{-2}

In $\alpha = 5$ when living cells are present (*i.e.*, for $j < j_{DZ}$), the surface area of each plasma membrane is given by,

$$\begin{aligned}
A^{p}_{5,j<j_{DZ}} = {} & \frac{2\pi}{N^{cells}_{\alpha=5,j}} \left(R_{\alpha=5} - \frac{t_{cw}}{2}\right)^2 \\
& \times \left(1 - \frac{\theta}{360}\right) \left(1 - F^{ax}_{\alpha=5}\right) \\
& + \frac{2\pi}{N^{cells}_{\alpha=5,j}} \left(R_{\alpha=5} - \frac{t_{cw}}{2}\right) \\
& \times \left(1 - \frac{\theta}{360}\right) \left(l^{ax}_{\alpha=5,j} - t_{cw}\right) \left(1 - F^{rad}_{\alpha=5}\right) \\
& + 2\left(R_{\alpha=5} - \frac{t_{cw}}{2}\right) \left(l^{ax}_{\alpha=5,j} - t_{cw}\right). \qquad \text{(S72)}
\end{aligned}$$

The vacuole volume changes over time in response to water flow rates, and hence, $A^{t}_{\alpha,j}$ also changes over time. Therefore, an expression is required to relate the changing vacuole volume to $A^{t}_{\alpha,j}$ at each time step. The total volume of each cell is constant and in $\alpha = 1, \ldots, 4$ is given by,

$$\begin{aligned}
V^{cell}_{\alpha,j} = {} & \frac{\pi}{N^{cells}_{\alpha,j}} \left[\left(R_{\alpha} - \frac{t_{cw}}{2}\right)^2 - \left(R_{\alpha+1} + \frac{t_{cw}}{2}\right)^2\right] \\
& \times \left(1 - \frac{\theta}{360}\right) \left(l^{ax}_{\alpha,j} - t_{cw}\right), \qquad \text{(S73)}
\end{aligned}$$

while the volume of each of the cells in $\alpha = 5$ is given by,

$$\begin{aligned}
V^{cell}_{5,j<j_{DZ}} = {} & \frac{\pi}{N^{cells}_{\alpha=5,j}} \left(R_{\alpha=5} - \frac{t_{cw}}{2}\right)^2 \left(1 - \frac{\theta}{360}\right) \\
& \times \left(l^{ax}_{\alpha=5,j} - t_{cw}\right). \qquad \text{(S74)}
\end{aligned}$$

The volume of the vacuole in each cell is determined at each time step of the solution (using Eq. (4)). Since the vacuole occupies a large fraction of the total cell volume it is important that the shape of the vacuole is chosen so that none of its dimensions exceed those of the cell. Hence it was assumed that each vacuole was a similar shape to the cell, with a reduced height and shortened curved sides. Hence, the volume of each vacuole in $\alpha = 1, \ldots, 4$ is given by,

$$\begin{aligned}
V^{v}_{\alpha,j} = {} & \frac{\pi x^2}{N^{cells}_{\alpha,j}} \left[\left(R_{\alpha} - \frac{t_{cw}}{2}\right)^2 - \left(R_{\alpha+1} + \frac{t_{cw}}{2}\right)^2\right] \\
& \times \left(1 - \frac{\theta}{360}\right) \left(l^{ax}_{\alpha,j} - t_{cw}\right), \qquad \text{(S75)}
\end{aligned}$$

and similarly the volume of each vacuole in $\alpha = 5$ is given by,

$$V^{v}_{5,j<j_{DZ}} = \frac{\pi x^2}{N^{cells}_{\alpha=5,j}} \left(R_{\alpha=5} - \frac{t_{cw}}{2}\right)^2 \left(1 - \frac{\theta}{360}\right) \times \left(l^{ax}_{\alpha=5,j} - t_{cw}\right), \tag{S76}$$

where, the height and curved sides of the vacuole are reduced by a factor x compared to the cell. Since $V^{cell}_{\alpha,j}$ and $V^{v}_{\alpha,j}$ are both known at every time step, x can be determined using $x = \left(V^{v}_{\alpha,j}/V^{cell}_{\alpha,j}\right)^{\frac{1}{2}}$ and hence $A^{t}_{\alpha,j}$ can be calculated for $\alpha = 1, \ldots, 4$ using,

$$\begin{aligned} A^{t}_{\alpha,j} = {} & \frac{2\pi x}{N^{cells}_{\alpha,j}} \left[\left(R_{\alpha} - \frac{t_{cw}}{2}\right)^2 - \left(R_{\alpha+1} + \frac{t_{cw}}{2}\right)^2\right] \\ & \times \left(1 - \frac{\theta}{360}\right) \\ & + \frac{2\pi x^2}{N^{cells}_{\alpha,j}} \left(R_{\alpha} - \frac{t_{cw}}{2}\right) \left(1 - \frac{\theta}{360}\right) \left(l^{ax}_{\alpha,j} - t_{cw}\right) \\ & + \frac{2\pi x^2}{N^{cells}_{\alpha,j}} \left(R_{\alpha+1} + \frac{t_{cw}}{2}\right) \left(1 - \frac{\theta}{360}\right) \left(l^{ax}_{\alpha,j} - t_{cw}\right) \\ & + 2x \left[\left(R_{\alpha} - \frac{t_{cw}}{2}\right) - \left(R_{\alpha+1} + \frac{t_{cw}}{2}\right)\right] \left(l^{ax}_{\alpha,j} - t_{cw}\right), \end{aligned} \tag{S77}$$

and for $\alpha = 5$ using,

$$\begin{aligned} A^{t}_{5,j<j_{DZ}} = {} & \frac{2\pi x}{N^{cells}_{\alpha=5,j}} \left(R_{\alpha=5} - \frac{t_{cw}}{2}\right)^2 \left(1 - \frac{\theta}{360}\right) \\ & + \frac{2\pi x^2}{N^{cells}_{\alpha=5,j}} \left(R_{\alpha=5} - \frac{t_{cw}}{2}\right) \left(1 - \frac{\theta}{360}\right) \\ & \times \left(l^{ax}_{\alpha=5,j} - t_{cw}\right) \\ & + 2x \left(R_{\alpha=5} - \frac{t_{cw}}{2}\right) \left(l^{ax}_{\alpha=5,j} - t_{cw}\right). \end{aligned} \tag{S78}$$

We remark that a more complete model would encompass cell and root volume changes. In this paper we have not included total cell volume changes primarily to simplify the computations, with the added benefit of allowing us to focus on the effects of membrane transporters alone. Fixed cell and root volume also allows for closer comparisons with results of our previous works (Foster and Miklavcic, 2013, 2014, 2015, 2016). In future work it would be interesting to explore the ramifications of cell expansion on salt compartmentalisation. However, the self-consistent inclusion of cell and therefore root volume changes requires an appreciation of how this may influence membrane transport, which is here assumed constant although distributed. The lack of knowledge of appropriate elastic moduli, degree of anisotropic expansion possible and interdependence of cell volume and membrane transport, all of which will be different for cells in different tissues and maturation zones, are reasons to postpone adding this feature to a future endeavor.

S.4. ADDITIONAL RESULTS: THE CASPARIAN STRIP SIMULATED AT THE CORTICAL-ENDODERMAL INTERFACE

Figures S2 and S3 show the effect of simulating the Casparian strip (CS) by altering the apoplastic transport parameters at the cortical-endodermal interface rather than the endodermal-pericycle interface. The CS simulated in this manner again has a dramatic impact on the steady-state water and Na^+ uptake (see Figure S3), as well as apoplastic ion concentrations in the inner regions of the root (see Figure S2). However, in this case the apoplastic ion concentrations in the endodermis are, not surprisingly, also reduced. Again, the suberin lamellae (SL) in the endodermis have only a minor influence on ion and water uptake (see Figure S3).

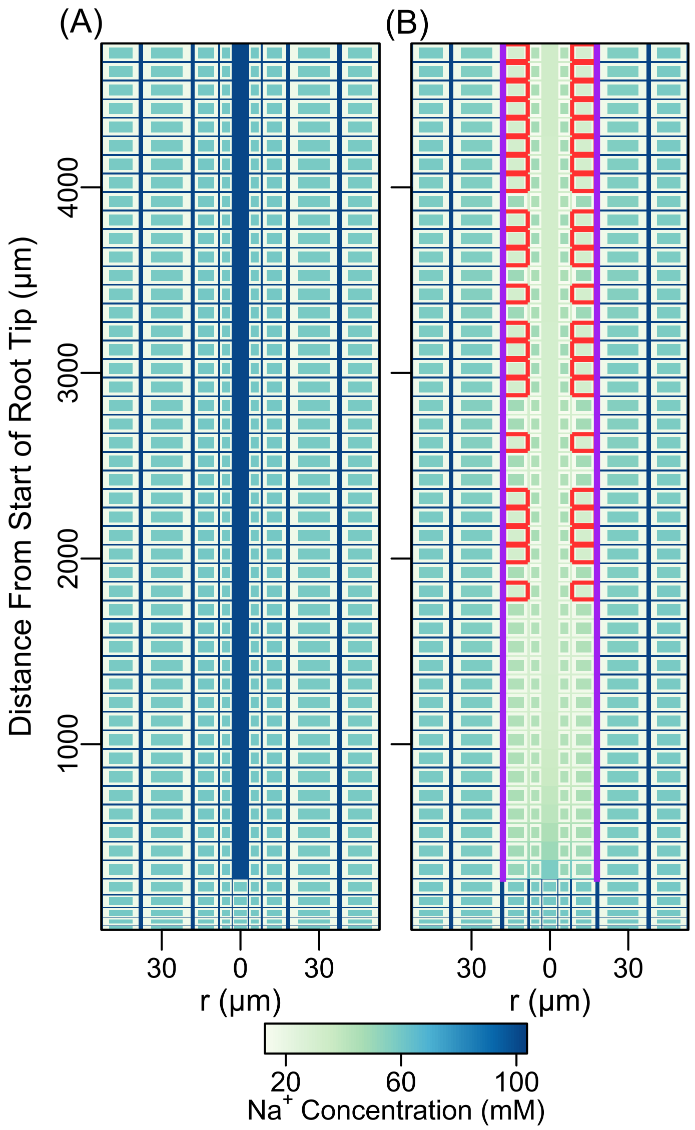

Figure S2. Plots of steady-state Na^+ concentrations for a uniform distribution of membrane transport proteins and two different root structures: **(A)** no endodermal barriers present, and **(B)** CS and SL with passage cells present. Purple (red) lines show the location of the CS (SL). Note that the effect of the CS is simulated at the cortical-endodermal interface. Results are shown for all three compartments (apoplast, cytoplasm and vacuole), although for clarity these compartments are not drawn to scale. $P_b = -0.5$ MPa and the remaining boundary conditions and transport parameters are as described in Sections 3.3 and 3.4, as well as Tables S1 and S2.

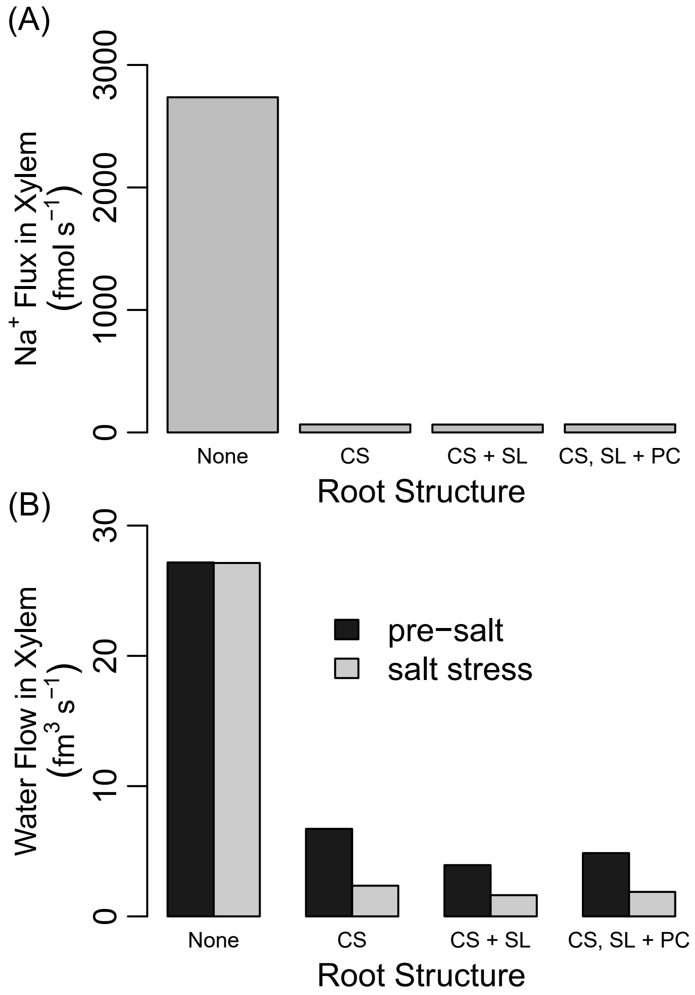

Figure S3. Plots of steady-state **(A)** Na^{+} fluxes in the xylem transpiration stream at the top of the root, and **(B)** pre-salt (black) and salt-stressed (gray) water flow rates in the transpiration stream at the top of the root, for a uniform distribution of membrane transport proteins and four different root structures: no endodermal barriers present; only the CS present; both CS and uninterrupted SL present; and CS, SL and passage cells present. Note that the effect of the CS is simulated at the cortical-endodermal interface. The simulations were conducted using $P_b = -0.5$ MPa, with the remaining boundary conditions and transport parameters are as described in Sections 3.3 and 3.4, as well as Tables S1 and S2. Note that since the model results are based on a constant difference between P_b and the hydraulic pressure in the external medium, there is equivalence between the above water fluxes and root conductances.

S.5. ADDITIONAL FIGURES

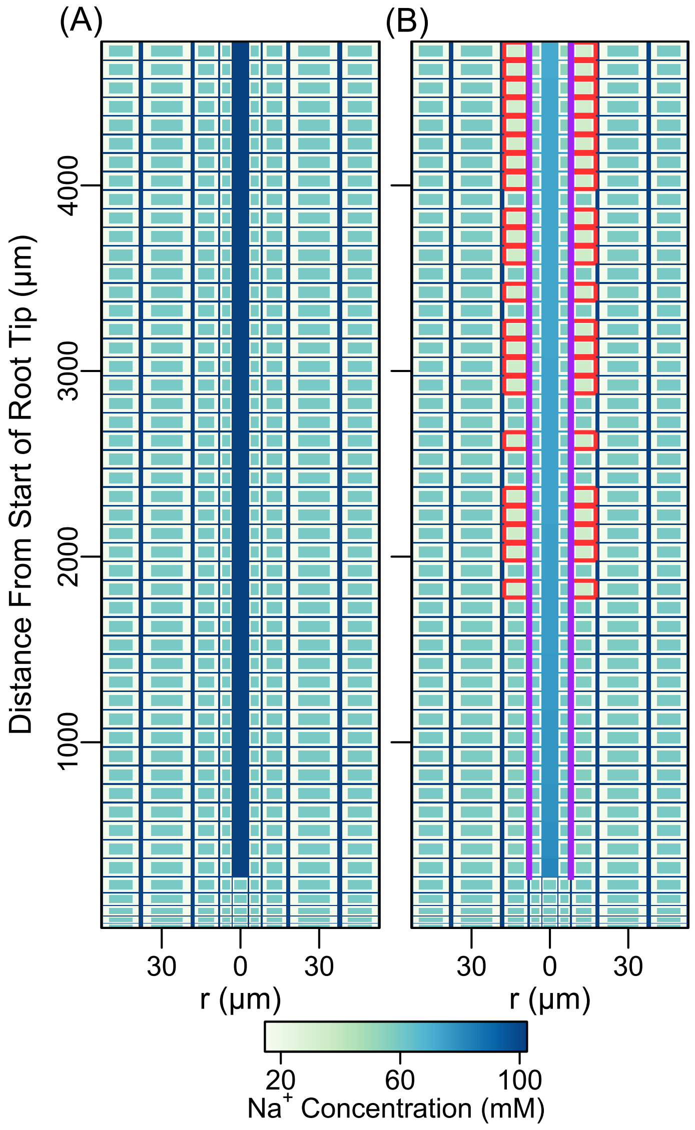

Figure S4. Plots of steady-state Na^+ concentrations for a uniform distribution of membrane transport proteins and two different root structures: **(A)** no endodermal barriers present, and **(B)** CS and SL with passage cells present. Purple (red) lines show the location of the CS (SL). Results are shown for all three compartments (apoplast, cytoplasm and vacuole), although for clarity these compartments are not drawn to scale. $P_b = -0.1$ MPa and the remaining boundary conditions and transport parameters are as described in Sections 3.3 and 3.4, as well as Tables S1 and S2.

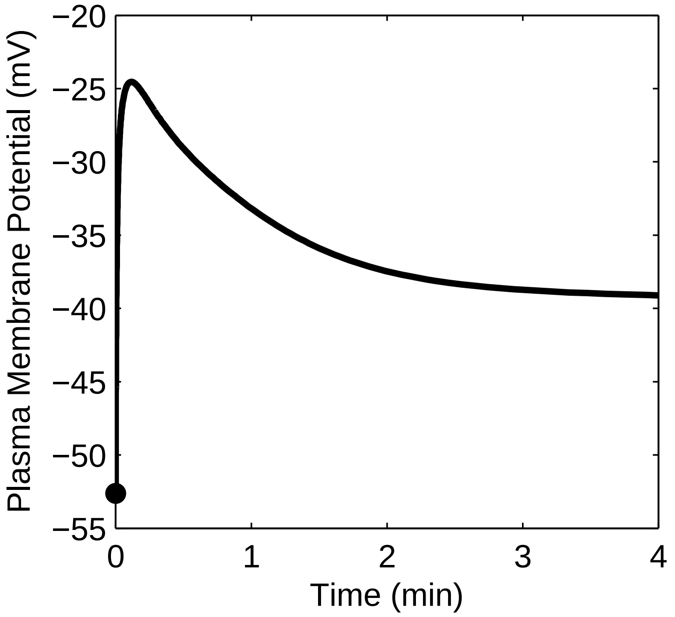

Figure S5. Plot showing the rapid depolarization of plasma membrane potentials in response to salt. The plasma membrane potential shown is for the epidermal cells halfway along the differentiation zone ($\alpha = 1, j = 28$). The simulation conditions are as described in Figure 3A.

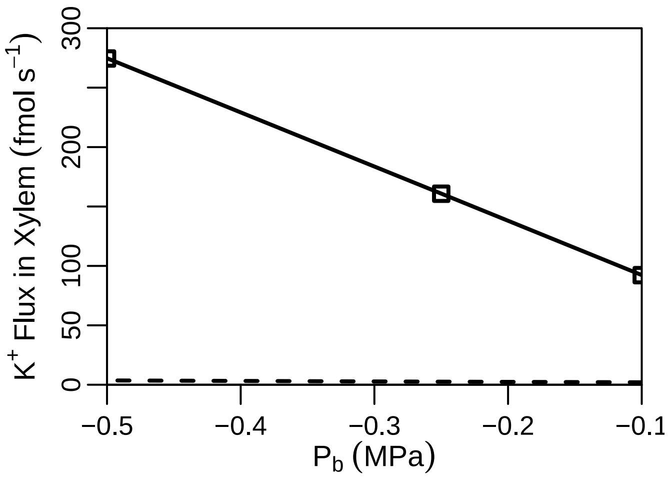

Figure S6. Plot of steady-state K^+ fluxes in the xylem transpiration stream at the top of the root as a function of P_b for three different root structures: no endodermal barriers (solid lines), CS only (dashed lines), and SL only (squares). Note that the results for simulations in which the CS and SL are both present are, using this scale, indistinguishable from the CS only results. The simulation conditions are as described in Figure 5.

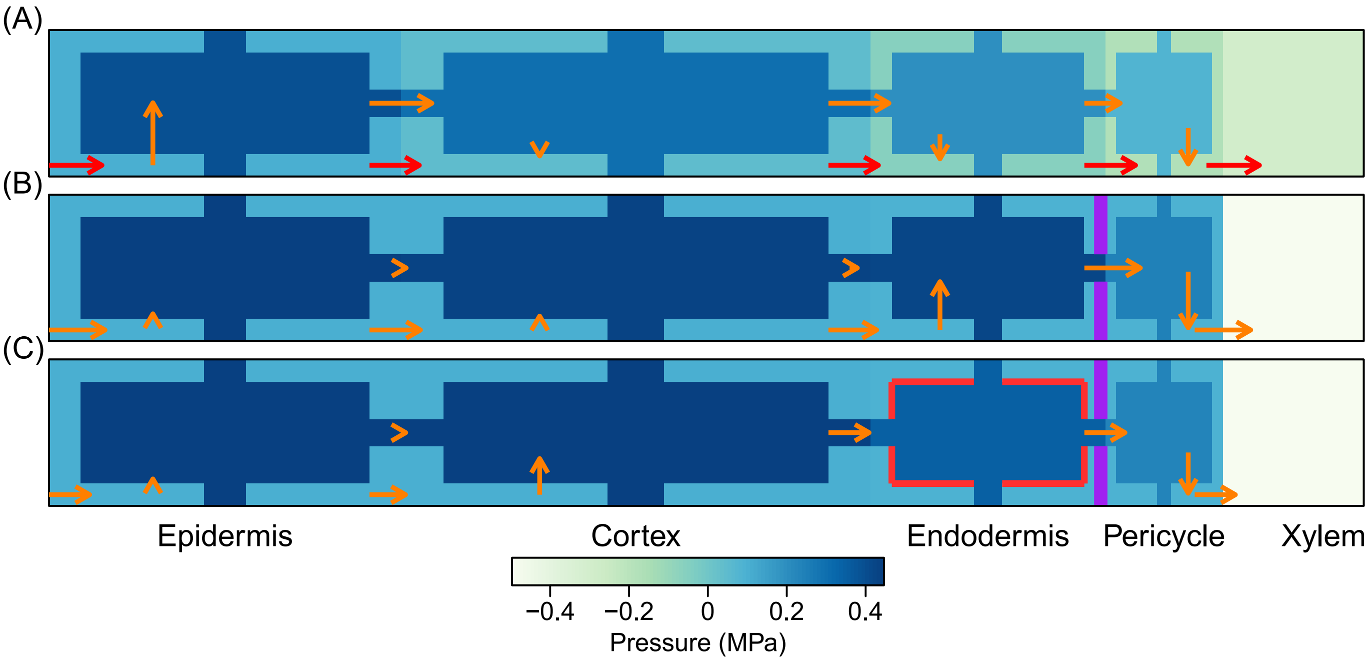

Figure S7. Plots of steady-state water flow rates (arrows) and hydraulic pressure (color maps) at a point halfway along the differentiation zone ($j = 28$), for a uniform distribution of membrane transport proteins and three different root structures: **(A)** no endodermal barriers present, **(B)** CS present, and **(C)** CS and SL present. Purple (red) lines show the location of the CS (SL). The arrows show the relative magnitudes of water flow rates via the apoplast, symplast and across the cell plasma membranes (for clarity, axial fluxes are not displayed). The orange arrows are drawn to the same scale across all three subfigures, while red arrows represent 20 times larger fluxes relative to the orange arrows. Note that hydraulic pressure results are shown for all three compartments (apoplast, cytoplasm and vacuole), although for clarity these compartments are not drawn to scale. The simulation conditions are as described in Figure 3.

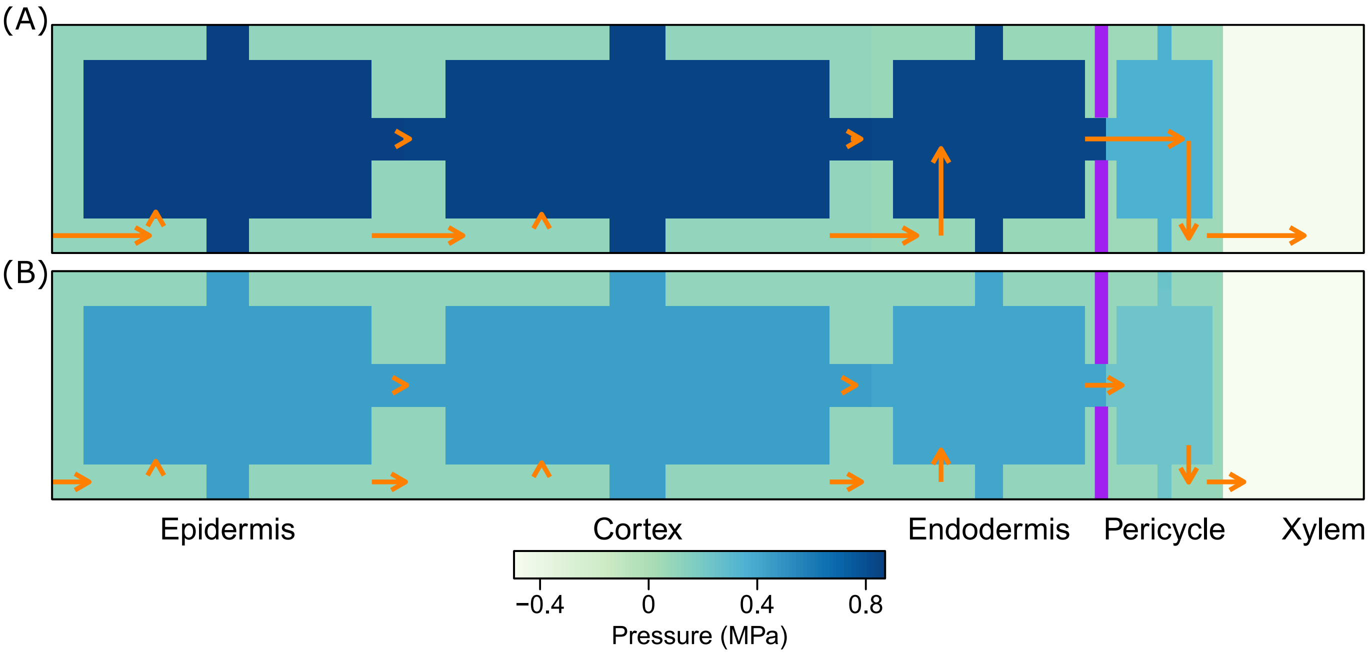

Figure S8. Plots of steady-state water flow rates (arrows) and hydraulic pressure (color maps) at a point halfway along the differentiation zone ($j = 28$), for a uniform distribution of membrane transport proteins and with the CS barrier present. The panels show water flow and hydraulic pressure before **(A)** and after **(B)** introduction of NaCl in the external medium. Purple lines show the location of the CS. The arrows show the relative magnitudes of water flow rates via the apoplast, symplast and across the cell plasma membranes (for clarity, axial fluxes are not displayed). The arrows are drawn to the same scale in both subfigures. Note that hydraulic pressure results are shown for all three compartments (apoplast, cytoplasm and vacuole), although for clarity these compartments are not drawn to scale. The simulation conditions are as described in Figure 4B.

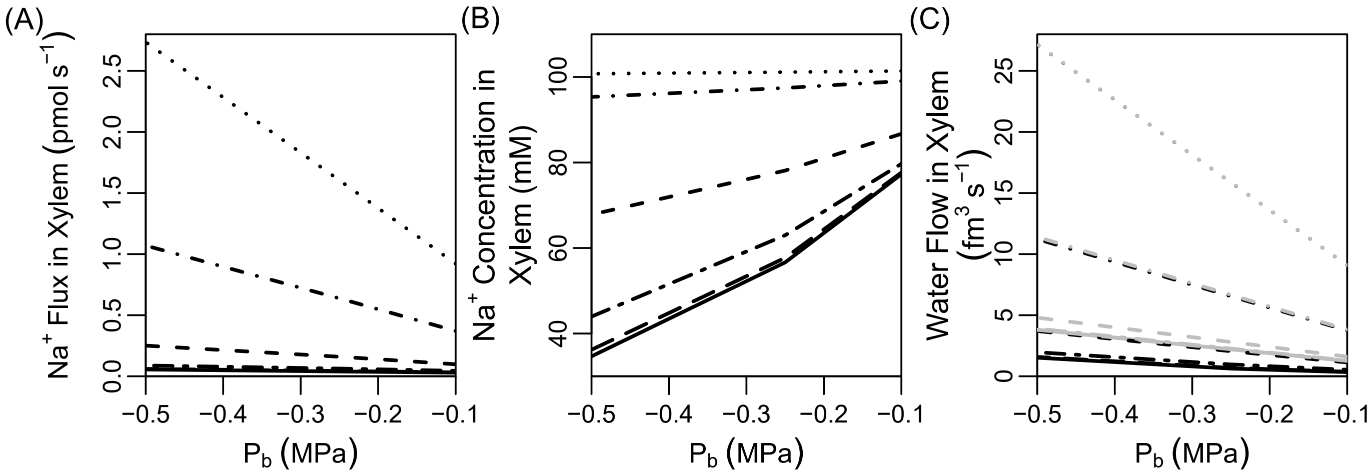

Figure S9. Plots of steady-state **(A)** Na^+ fluxes, **(B)** Na^+ concentrations, and **(C)** pre-salt (gray lines) and salt stress (black lines) water flow rates in the transpiration stream at the top of the root, as a function of P_b for CSs with a range of permeabilities. The line types indicate the effectiveness of the CS and represent the following scenarios: no CS present (dotted lines); $k^{a,rad,n}_{\alpha=4,j_{CS}}$ and $L^{a,rad}_{p:\alpha=4,j_{CS}}$ reduced by one order of magnitude compared to their values in the no CS case (short dot-dashed lines); $k^{a,rad,n}_{\alpha=4,j_{CS}}$ and $L^{a,rad}_{p:\alpha=4,j_{CS}}$ reduced by two orders of magnitude (short dashed lines); $k^{a,rad,n}_{\alpha=4,j_{CS}}$ and $L^{a,rad}_{p:\alpha=4,j_{CS}}$ reduced by three orders of magnitude (long dot-dashed lines); $k^{a,rad,n}_{\alpha=4,j_{CS}}$ and $L^{a,rad}_{p:\alpha=4,j_{CS}}$ reduced by four orders of magnitude (long dashed lines); and completely impermeable CS (solid lines). To investigate the influence of the CS only, the SL were excluded from all simulations. The remaining transport parameters and boundary conditions are as described in Sections 3.3 and 3.4, as well as Tables S1 and S2.

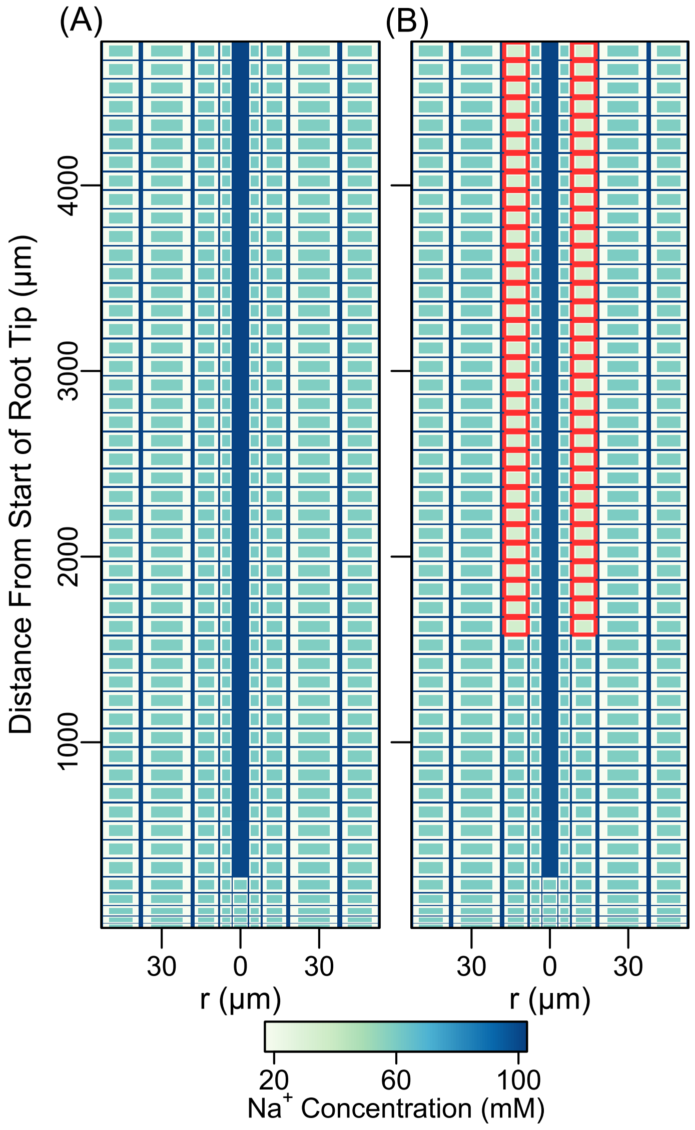

Figure S10. Plots of steady-state Na^+ concentrations for a uniform distribution of membrane transport proteins and two different root structures: **(A)** no endodermal barriers present, and **(B)** SL only present. Red lines show the location of the SL. Results are shown for all three compartments (apoplast, cytoplasm and vacuole), although for clarity these compartments are not drawn to scale. $P_b = -0.5$ MPa and the remaining boundary conditions and transport parameters are as described in Sections 3.3 and 3.4, as well as Tables S1 and S2.

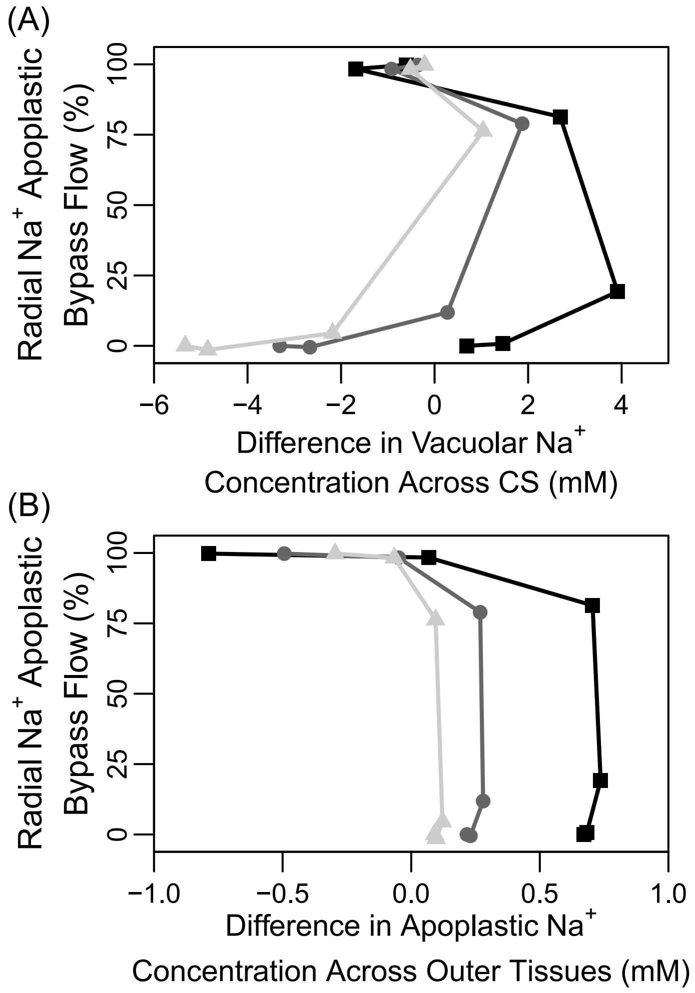

Figure S11. Plots of steady-state percentage of Na^+ flux across the endodermis-pericycle interface occurring via the apoplast (apoplastic bypass flow) versus **(A)** the difference in vacuolar Na^+ concentration across the CS (the vacuolar Na^+ concentration in the endodermis minus the vacuolar Na^+ concentration in the pericycle), and **(B)** the difference in apoplastic Na^+ concentration across the outer tissues (the apoplastic Na^+ concentration in the endodermis minus the apoplastic Na^+ concentration in the epidermis), based on the range of partially permeable CSs shown in Figure S9. The results are shown for three different levels of transpiration: $P_b = -0.1$ MPa (light gray triangles), $P_b = -0.25$ MPa (dark gray circles) and $P_b = -0.5$ MPa (black squares). The percentage of apoplastic bypass flow of Na^+ was determined using: $100\times$(radial apoplastic flux of Na^+ across the endodermis-pericycle interface)/total radial Na^+ flux across the endodermis-pericycle interface; where the total flux is the sum of the symplastic and apoplastic fluxes. All concentration values were taken at a point halfway along the DZ ($j = 28$). The simulation conditions are as described in Figure S9.

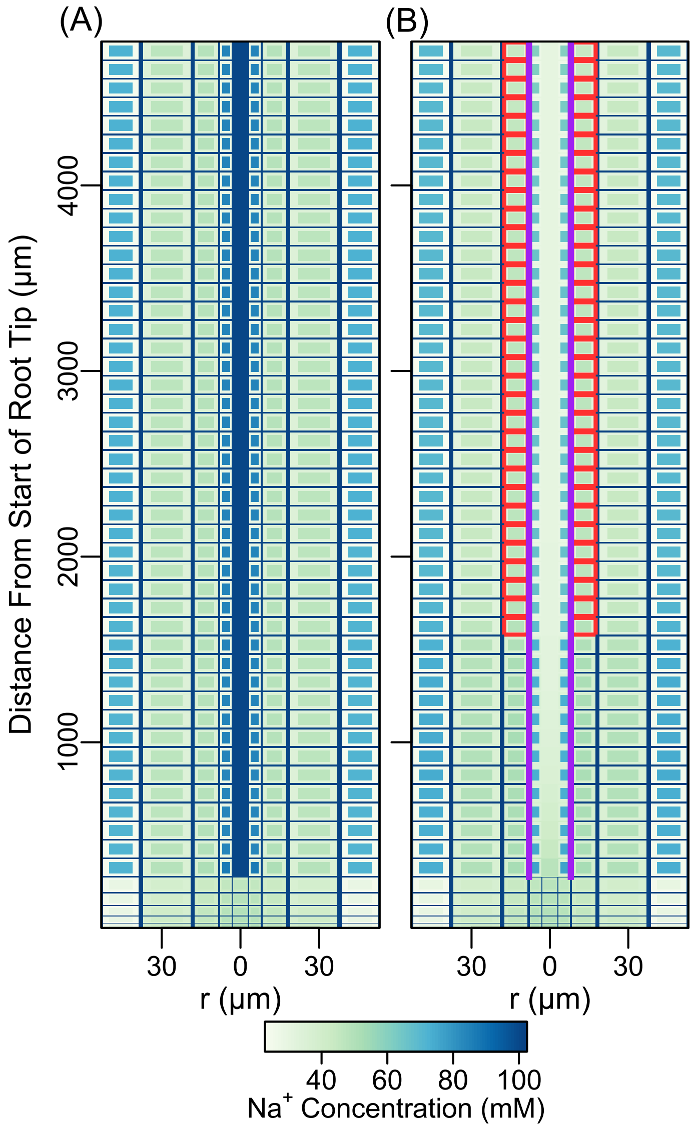

Figure S12. Plots of steady-state Na^+ concentrations for the nonuniform distribution of membrane transport proteins described in Section 3.4 and two different root structures: **(A)** no endodermal barriers present, and **(B)** CS and SL present. Purple (red) lines show the location of the CS (SL). Results are shown for all three compartments (apoplast, cytoplasm and vacuole), although for clarity these compartments are not drawn to scale. The simulations were conducted using $P_b = -0.5$ MPa, with the remaining boundary conditions and transport parameters are as described in Sections 3.3 and 3.4, as well as Tables S1 and S2.

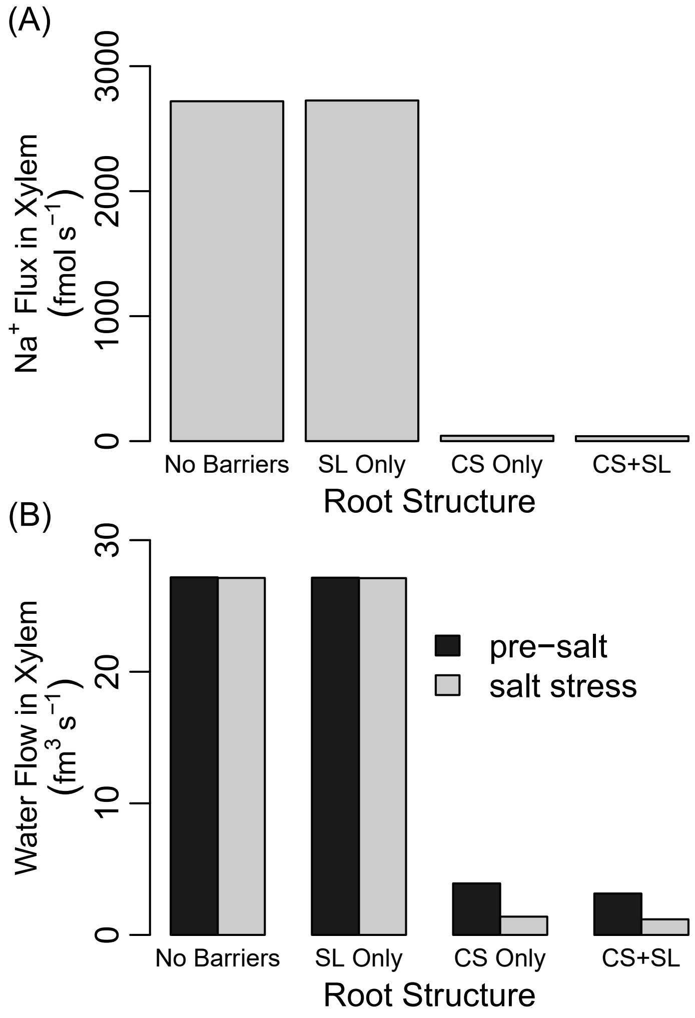

Figure S13. Plots of steady-state **(A)** Na^+ fluxes in the xylem transpiration stream at the top of the root, and **(B)** pre-salt (black) and salt-stressed (gray) water flow rates in the transpiration stream at the top of the root, for the nonuniform distribution of membrane transport proteins described in Section 3.4 and four different root structures: no endodermal barriers present; only the SL present; only the CS present; and both the CS and SL present. The simulations were conducted using $P_b = -0.5$ MPa, with the remaining boundary conditions and transport parameters are as described in Sections 3.3 and 3.4, as well as Tables S1 and S2.

REFERENCES

Amtmann, A., Jelitto, T. C., Sanders, D., 1999. K^+-selective inward-rectifying channels and apoplastic pH in barley roots. Plant Physiology 120 (1), 331–338.

Anderson, W. P., 1976. Physico-Chemical Assessment of Plasmodesmatal Transport. Springer Berlin Heidelberg, Berlin, Heidelberg, pp. 107–120.

Beilby, M. J., Walker, N. A., 1981. Chloride transport in *Chara*: I. kinetics and current-voltage curves for a probable proton symport. Journal of Experimental Botany 32 (1), 43–54.

Blatt, M., Rodriguez-Navarro, A., Slayman, C., 1987. Potassium-proton symport in *Neurospora*: kinetic control by pH and membrane potential. The Journal of Membrane Biology 98 (2), 169–189.

Brini, F., Hanin, M., Mezghani, I., Berkowitz, G. A., Masmoudi, K., 2007. Overexpression of wheat Na^+/H^+ antiporter *TNHX1* and H^+-pyrophosphatase *TVP1* improve salt- and drought-stress tolerance in *Arabidopsis thaliana* plants. Journal of Experimental Botany 58 (2), 301–308.

Casimiro, I., Beeckman, T., Graham, N., Bhalerao, R., Zhang, H., Casero, P., Sandberg, G., Bennett, M. J., 2003. Dissecting *Arabidopsis* lateral root development. Trends Plant Sci. 8 (4), 165–171.

Chen, Z., Pottosin, I. I., Cuin, T. A., Fuglsang, A. T., Tester, M., Jha, D., Zepeda-Jazo, I., Zhou, M., Palmgren, M. G., Newman, I. A., Shabala, S., 2007. Root plasma membrane transporters controlling K^+/Na^+ homeostasis in salt-stressed barley. Plant Physiology 145 (4), 1714–1725.

Chowdhury, S., Chanda, B., 2012. Estimating the voltage-dependent free energy change of ion channels using the median voltage for activation. The Journal of General Physiology 139 (1), 3–17.

Dechadilok, P., Deen, W. M., 2006. Hindrance factors for diffusion and convection in pores. Industrial & Engineering Chemistry Research 45 (21), 6953–6959.

Dolan, L., Janmaat, K., Willemsen, V., Linstead, P., Poethig, S., Roberts, K., Scheres, B., 1993. Cellular organisation of the *Arabidopsis thaliana* root. Development 119 (1), 71–84.

Ehlers, K., Bel, A. J. E., 1999. The Physiological and Developmental Consequences of Plasmodesmal Connectivity. Springer Berlin Heidelberg, Berlin, Heidelberg, pp. 243–260.

Ehlers, K., Kollmann, R., 2001. Primary and secondary plasmodesmata: structure, origin, and functioning. Protoplasma 216 (1), 1–30.

Evans, D., 2009. Osmotic and Ionic Regulation: Cells and Animals. CRC Press, Florida.

Felle, H. H., 1994. The H^+/Cl^- symporter in root-hair cells of *Sinapis alba* (an electrophysiological study using ion-selective microelectrodes). Plant Physiology 106 (3), 1131–1136.

Foster, K., Miklavcic, S. J., 2013. Mathematical modelling of the uptake and transport of salt in plant roots. Journal of Theoretical Biology 336, 132–143.

Foster, K. J., Miklavcic, S. J., 2014. On the competitive uptake and transport of ions through differentiated root tissues. Journal of Theoretical Biology 340, 1–10.

Foster, K. J., Miklavcic, S. J., 2015. Toward a biophysical understanding of the salt stress response of individual plant cells. Journal of Theoretical Biology 385, 130–142.

Foster, K. J., Miklavcic, S. J., 2016. Modeling root zone effects on preferred pathways for the passive transport of ions and water in plant roots. Frontiers in Plant Science 7, 1–14.

George, E., Hall, A., de Klerk, G., 2007. Plant Propagation by Tissue Culture: Volume 1. The Background. Springer Netherlands.

Grignon, C., Sentenac, H., 1991. pH and ionic conditions in the apoplast. Annual Review of Plant Physiology and Plant Molecular Biology 42 (1), 103–128.

Hansen, U.-P., Gradmann, D., Sanders, D., Slayman, C., 1981. Interpretation of current-voltage relationships for "active" ion transport systems: I. steady-state reaction-kinetic analysis of class-I mechanisms. The Journal of Membrane Biology 63 (3), 165–190.

Harris, D. C., 2007. Quantitative Chemical Analysis, 7th Edition. W. H. Freeman and Company, USA.

Haynes, W. M., 2015. CRC Handbook of Chemistry and Physics, 96th Edition. CRC press, Boca Raton.

Hills, A., Chen, Z.-H., Amtmann, A., Blatt, M. R., Lew, V. L., 2012. OnGuard, a computational platform for quantitative kinetic modeling of guard cell physiology. Plant Physiology 159 (3), 1026–1042.

Ivashikina, N., Becker, D., Ache, P., Meyerhoff, O., Felle, H. H., Hedrich, R., 2001. K^+ channel profile and electrical properties of *Arabidopsis* root hairs. FEBS Letters 508 (3), 463–469.

Javot, H., Lauvergeat, V., Santoni, V., Martin-Laurent, F., Güçlü, J., Vinh, J., Heyes, J., Franck, K. I., Schäffner, A. R., Bouchez, D., Maurel, C., 2003. Role of a single aquaporin isoform in root water uptake. The Plant Cell Online 15 (2), 509–522.

Katchalsky, A., Curran, P. F., 1965. Nonequilibrium Thermodynamics in Biophysics. Harvard University Press, Cambridge.

Kavitha, P. G., Miller, A. J., Mathew, M. K., Maathuis, F. J. M., 2012. Rice cultivars with differing salt tolerance contain similar cation channels in their root cells. Journal of Experimental Botany 63 (8), 3289–3296.

Keener, J., Sneyd, J., 2009. Mathematical Physiology I: Cellular Physiology, 2nd Edition. Springer, New York.

Kramer, E. M., Frazer, N. L., Baskin, T. I., 2007. Measurement of diffusion within the cell wall in living roots of *Arabidopsis thaliana*. Journal of Experimental Botany 58 (11), 3005–3015.

Kronzucker, H. J., Britto, D. T., 2011. Sodium transport in plants: a critical review. New Phytologist 189 (1), 54–81.

Liesche, J., Schulz, A., 2013. Modeling the parameters for plasmodesmal sugar filtering in active symplasmic phloem loaders. Frontiers in Plant Science 4, 1–11.

Maathuis, F. J. M., Sanders, D., Gradmann, D., 1997. Kinetics of high-affinity K^+ uptake in plants, derived from K^+-induced changes in current-voltage relationships. Planta 203 (2), 229–236.

Mattsson, J., Sung, Z., Berleth, T., 1999. Responses of plant vascular systems to auxin transport inhibition. Development 126 (13), 2979–2991.

Michael, W., Schultz, A., Meshcheryakov, A. B., Ehwald, R., 1997. Apoplasmic and protoplasmic water transport through the parenchyma of the potato storage organ. Plant Physiology 115 (3), 1089–1099.

Miklavcic, S. J., Nyden, M., Linden, J. B., Schulz, J., 2014. Mathematically modelling competititve ion absorption in a polymer matrix. RSC Advances 4, 60349.

Miklavic, S. J., Ninham, B. W., 1990. Competition for adsorption sites by hydrated ions. J. Colloid Interf. Sci. 134, 305–311.

Murphy, R., 1989. Water-flow across the sieve-tube boundary - estimating turgor and some implications for phloem loading and unloading. I. Theory. Annals of Botany 63 (5), 541–549.

Roberts, S. K., Tester, M., 1995. Inward and outward K^+-selective currents in the plasma membrane of protoplasts from maize root cortex and stele. The Plant Journal 8 (6), 811–825.

Scheres, B., Di Laurenzio, L., Willemsen, V., Hauser, M. T., Janmaat, K., Weisbeek, P., Benfey, P. N., 1995. Mutations affecting the radial organisation of the *Arabidopsis* root display specific defects throughout the embryonic axis. Development 121 (1), 53–62.

Steudle, E., 1994. Water transport across roots. Plant and Soil 167 (1), 79–90.

Sukhov, V., Vodeneev, V., 2009. A mathematical model of action potential in cells of vascular plants. Journal of Membrane Biology 232 (1-3), 59–67.

van der Horst, H. C., Timmer, J. M. K., Robbertsen, T., Leenders, J., 1995. Use of nanofiltration for concentration and demineralization in the dairy industry: Model for mass transport. J. Membrane Sci. 104 (3), 205–218.

Wegner, L. H., De Boer, A. H., 1997. Properties of two outward-rectifying channels in root xylem parenchyma cells suggest a role in K^+ homeostasis and long-distance signaling. Plant Physiology 115 (4), 1707–1719.

Wegner, L. H., De Boer, A. H., Raschke, K., 1994. Properties of the K^+ inward rectifier in the plasma membrane of xylem parenchyma cells from barley roots: Effects of TEA^+, Ca^{2+}, Ba^{2+} and La^{3+}. The Journal of Membrane Biology 142 (3), 363–379.

White, P. J., Lemtiri-Chlieh, F., 1995. Potassium currents across the plasma membrane of protoplasts derived from rye roots: a patch-clamp study. Journal of Experimental Botany 46 (5), 497–511.

Zhu, G. L., Steudle, E., 1991. Water transport across maize roots: Simultaneous measurement of flows at the cell and root level by double pressure probe technique. Plant Physiology 95 (1), 305–315.

Zhu, T., Lucas, W. J., Rost, T. L., 1998. Directional cell-to-cell communication in the *Arabidopsis* root apical meristem I. An ultrastructural and functional analysis. Protoplasma 203 (1), 35–47.
